# Socioeconomic drivers of encephalitis burden in the post-COVID era: a 204-country analysis from global burden of disease study 2021

**DOI:** 10.3389/fpubh.2025.1651734

**Published:** 2025-09-18

**Authors:** Yikang Wang, Di Wang, Yu Tian, Yilong Yao, Qi Yu

**Affiliations:** Department of Neurosurgery, Shengjing Hospital of China Medical University, Shenyang, China

**Keywords:** encephalitis, global burden of disease, systematic analysis, socioeconomic drivers, post-COVID era

## Abstract

**Background:**

Encephalitis, an inflammatory central nervous system disease causing significant morbidity and mortality, disproportionately affects low- and middle-income countries (LMICs) due to healthcare disparities. Encephalitis has diverse etiologies—viral, autoimmune, bacterial, parasitic—each with distinct clinical and epidemiological features. Despite declining global age-standardized rates since 1990, inequities in diagnostics, vaccine coverage, and critical care persist, worsened by COVID-19 pandemic, which delayed diagnoses and disrupted vaccinations.

**Methods:**

Using Global Burden of Disease (GBD) 2021 data, we analyzed age-standardized prevalence, incidence, mortality, and disability-adjusted life-years (DALYs) across 204 countries (1990–2021). We used the Bayesian Age-Period-Cohort model with integrated nested Laplace approximation to predict encephalitis’ future trends, through 2040, enhancing the study’s predictive value. Sociodemographic Index (SDI) stratification and Bayesian meta-regression models assessed trends, with significance determined via 95% uncertainty intervals and estimated annual percentage change (EAPC).

**Results:**

In 2021, 4.64 million individuals worldwide were affected by encephalitis (1.49 million new cases; 92,000 deaths), encompassing cases spanning acute, subacute, and chronic stages of the disease. Low-middle SDI regions bore 3–5 times higher burdens than high-SDI regions. South Asia had the highest burden (age-standardized prevalence rate [ASPR]: 140.9/100,000; incidence [ASIR]: 51.3/100,000), while Australasia reported the lowest (ASPR: 1.94/100,000). High-SDI countries showed distinct patterns, such as rising incidence in Australia. COVID-19 was associated with an 18% increase in DALYs in high-burden regions. National disparities were stark: Pakistan, India, and Nepal had the highest burdens; Canada, the lowest. The encephalitis burden was greater in children than in other age groups.

**Conclusion:**

This analysis advances prior GBD research by integrating post-COVID-19 insights and future burden forecasts, filling pre-pandemic study gaps. GBD dataset does not differentiate etiological subtypes, limiting our analysis granularity given encephalitis’ clinical and epidemiological heterogeneity. Socioeconomic inequities drive encephalitis burden, necessitating targeted interventions: scaling Japanese encephalitis vaccination in South Asia, strengthening African diagnostic hubs, and integrating climate-resilient surveillance. Post-pandemic recovery must prioritize healthcare infrastructure, telehealth, and policies addressing poverty and education. Global collaboration is critical to mitigate disparities and optimize region-specific strategies.

## Introduction

Encephalitis, an inflammatory condition of the central nervous system with high mortality and morbidity rates, is categorized into viral, bacterial, fungal, and autoimmune subtypes. Its symptoms typically include alterations in consciousness, behavior, or even personality as well as focal neurological deficits such as epileptic seizures; additionally, acute hyperthermia is a predominant symptom. Survivors, particularly children, may develop more severe neurological sequelae, including intellectual impairment and developmental delays (1, 2). Encephalitis imposes a substantial social and economic burden globally and remains a major public health concern worldwide (3, 4). The average mortality rate among encephalitis patients is approximately 5%, with this rate reaching around 10% in high-income countries (HICs). Over 70% of survivors experience persistent residual symptoms, such as neurological dysfunction (5). At the national level, a systematic analysis indicates that the United States records an average of 20,258 encephalitis-related hospitalizations annually, with approximately 10.1% of cases resulting in death; the total annual hospitalization costs associated with encephalitis are estimated at $2 billion (6). Although studies have shown a slight decline in the incidence and mortality rates of this disease since 1990 (7), further research is required to develop effective prevention and treatment strategies.

Advances in molecular diagnostics—including cerebrospinal fluid polymerase chain reaction (CSF PCR) and metagenomic sequencing—have revolutionized the management of encephalitis in high-resource settings (8). However, critical gaps persist in LMICs. For instance, fewer than 30% of healthcare facilities in Sub-Saharan Africa (e.g., the Congo, Nigeria) can perform routine herpes simplex virus (HSV) testing, delaying the administration of life-saving acyclovir beyond the critical 6-h window (9, 10). A 2023 WHO report emphasizes that delayed HSV diagnosis in LMICs increases mortality by 30% compared to high-income countries (HICs). Similarly, despite the 85% efficacy of the Japanese encephalitis (JE) vaccine, coverage remains below 50% in rural South Asia, perpetuating a high burden among children (11).

The COVID-19 pandemic exacerbated these inequities. In LMICs, resource diversion toward pandemic response reduced encephalitis admissions by 40% between 2020 and 2021, delaying diagnoses and increasing severe sequelae. In Pakistan, lockdowns disrupted JE vaccination campaigns, leading to a 15% coverage decline (12). Conversely, high-income regions such as Australia observed rising encephalitis incidence (+114% since 1990), attributed to the expanded use of next-generation sequencing (NGS) during the pandemic (13). These disparities underscore the interplay between socioeconomic determinants—including healthcare access, vaccine equity, and diagnostic capacity—and disease outcomes.

This study leverages data from the Global Burden of Disease (GBD) 2021 to quantify the burden of encephalitis across 204 countries from 1990 to 2021. While GBD data aggregates encephalitis across etiological subtypes (e.g., viral, autoimmune), we prioritized this approach for its unique value: it provides the most comprehensive standardized dataset for cross-country comparisons over three decades, which is critical for identifying macro-level socioeconomic disparities in disease burden. By analyzing age-standardized prevalence, incidence, mortality, and disability-adjusted life-years (DALYs), we aim to identify regions where socioeconomic factors drive disproportionate burdens, evaluate the impact of COVID-19 on encephalitis management, and propose targeted strategies to address gaps in diagnostics, vaccination, and post-pandemic recovery. We acknowledge, however, that the lack of stratification by type limits disease-specific recommendations, as etiological differences (e.g., vaccine-preventable vs. immune-mediated) require distinct interventions. We opted not to stratify by encephalitis type to prioritize the most comprehensive standardized dataset for cross-country comparisons over three decades, which was critical for identifying macro-level socioeconomic disparities in disease burden.

## Materials and methods

### Data source

The study utilized GBD 2021 data, which quantifies encephalitis burden (prevalence, incidence, mortality, DALYs) across 204 countries (1990–2021) using ICD-10 criteria (14). Publicly accessible via the Global Health Data Exchange (GHDx), the data adhere to standardized reporting guidelines (15). Ethical approval and the requirement for informed consent to access the GBD data were waived by the University of Washington Institutional Review Board as analyses involved aggregated, anonymized data. The use of the 2021 Global Burden of Disease database in this study complied with the ethical standards of its governing body. As this study analyzed publicly available, de-identified, and fully anonymized data—with no inclusion of local personal information, clinical data, or similar data—no additional ethical approval was required.

### Estimation framework

The GBD 2021 study employs appropriate modeling techniques and sophisticated processes to assess the global burden of encephalitis. From this dataset, we extracted data on prevalence, incidence, mortality, and DALYs spanning 1990 to 2021. Incidence and prevalence rates were calculated using the DisMod-MR 2.1 model—a Bayesian meta-regression framework for GBD modeling—which integrates data on location, year, age, and sex to estimate these metrics. Briefly, mortality-to-incidence ratios (MIRs) were derived from vital registration, the cancer registry, and verbal autopsy; estimated mortality rates were then calculated based on these MIRs. For our study, the Cause of Death Ensemble model (CODEm) framework was used to estimate mortality rates and counts for each location, year, age group, and sex. CODEm data were generated through covariate selection and out-of-sample validity analyses. Through these models, GBD studies can provide comprehensive disease estimates (16, 17).

DALYs are a widely used metric for measuring disease burden, encompassing years of life lost (YLLs) due to premature death and years lived with disability (YLDs). By examining DALYs in a specific year, we can assess and compare the magnitude of a disease’s health impact and its contribution to overall disease burden within that year. Furthermore, to evaluate uncertainty, GBD data undergo 500 iterations per calculation, with the average of these iterations used as the estimated value. Years lived with disability (YLDs) were computed by multiplying disability weights for each of four phases (diagnosis and primary therapy, controlled phase, metastatic phase, and terminal phase) by the prevalence of sequelae. Years of life lost (YLLs) were calculated as the number of deaths in the population multiplied by the standard life expectancy at the time of death (14).

Our projections assumed static intervention rates (e.g., vaccination, diagnostics) but accounted for population growth and urbanization; climate factors were excluded due to data limits. The model also assumed a stable etiological distribution.

### Sociodemographic index (SDI)

The sociodemographic index (SDI) is designed to quantify the development level of countries or regions. It assesses the relationship between development level and disease burden by calculating an index ranging from 0 to 1, which integrates three metrics: the total fertility rate among individuals under 25, the mean education level among individuals aged 15 and older, and lag-distributed per capita income. A higher SDI value indicates a higher level of socioeconomic development. In our study, 204 countries and territories were categorized into five SDI-based regions (low, low-middle, middle, middle-high, and high) in the GBD 2021 dataset to examine the association between socioeconomic development and the burden of encephalitis (14).

### Statistical analysis

In this study, we used the age-standardized prevalence rate (ASPR), age-standardized incidence rate (ASIR), age-standardized mortality rate (ASMR), and age-standardized DALY rate (ASDR) to analyze the burden of encephalitis. Age-standardized rates are derived from linear regression models adjusted for the global age structure, and they are widely used in research on time trends and population characteristics of disease burden (14). The formula for the age-standardized rate is expressed as *y* = *α* + *βx* + *ε*, where *y* = ln (age-standardized rate), *x* = calendar year, *α* = intercept, *β* = slope, and *ε* = random error. Additionally, we used the estimated annual percentage change (EAPC) to assess the encephalitis burden and reflect its temporal trends. The EAPC was calculated as 100 × (e*^β^* − 1), with the 95% confidence interval (CI) also computed. The EAPC value reflects the trend of changes in age-standardized rates: if the EAPC and the lower bound of the 95% CI are positive, the age-standardized rate shows an upward trend; conversely, if the EAPC and the upper bound of the 95% CI are negative, the age-standardized rate shows a downward trend. To predict future trends in encephalitis, we employed the Bayesian Age-Period-Cohort (BAPC) model, which integrates nested Laplace approximation. Previous studies have demonstrated that the BAPC model has a broader application range and higher accuracy compared to other forecasting methods (18, 19).

All the age-standardized rates were reported per 100,000 population each year. The 95% uncertainty interval (UI) is defined by the 2.5th and 97.5th percentiles, which represent the 25th and 975th values of an ordered set of 1,000 estimates, respectively. A 95% UI excluding zero was considered statistically significant. *p* < 0.05 (2-sided) was used as the level of statistical significance. All data were analyzed and visualized using R software (version 4.4.1).

## Results

### Global level

**Figure 1:**
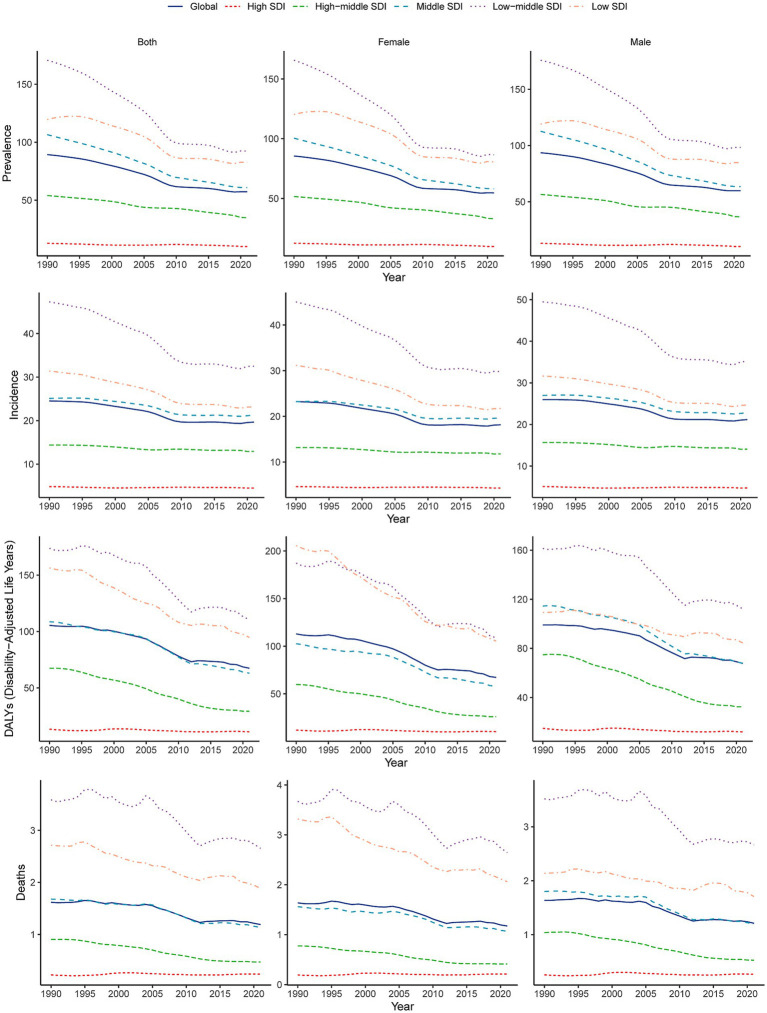
Temporal trends in age-standardized incidence, prevalence, DALYs, and mortality rates of encephalitis by sex (males and females) and SDI (high-SDI, high-middle-SDI, middle-SDI, low-middle-SDI, and low-SDI categories) from 1990 to 2021. SDI, sociodemographic index.

In 2021, the global burden of encephalitis improved but was still considered severe, with a total of 4,643,564.3 cases reported (95% UI: 3,115,878.4–5,999,959.1), an increase of 0.4% since 1990 (4,625,610.2 cases; 95% UI: 3,115,878.4–6,093,198.0). Despite a slight increase in the absolute number of cases, the ASPR decreased from 89.3 cases per 100,000 population in 1990 (95% UI: 59.4–117.9) to 57.3 cases per 100,000 population in 2021 (95% UI: 40.2–73.9). The EAPC for ASPR was −1.69 (95% CI: −1.58– −1.8) from 1990 to 2021 (Figure 1; Table 1). The global incidence of encephalitis in 2021 was estimated to be 1,492,767.7 cases (95% UI: 1,364,664.3–1,654,928.6), an increase of 10.06% from 1990 (1,356,348 cases; 95% UI: 1,228,368.4–1,525,339.8). The ASIR of encephalitis decreased from 24.5 cases per 100,000 population (95% UI: 22.6–27.3) in 1990 to 19.7 cases per 100,000 population (95% UI: 18.0–21.8) in 2021. The EAPC in the ASIR was −0.96 (95% CI: −1.06– −0.86) during this period of our study (Figure 1; Table 1). The estimated number of deaths from encephalitis was 84,188.9 (95% UI: 66,553.7–95,321.8) in 1990 and 91,947.7 (95% UI: 78,328.7–106,091.3) in 2021; in addition, despite the relatively small number of deaths overall, the number of deaths increased by 9.22%. The ASMR for encephalitis was estimated to be 1.19 per 100,000 population (95% UI: 1.01–1.37) in 2021 and 1.62 per 100,000 population (95% UI: 1.29–1.81) in 1990 (Supplementary Table S1; Figure 1). The global DALYs was 6,065,443.4 (95% UI: 4,742,546.8–6,962,738.7) in 1990 and 4,952,818.5 (95% UI: 4,095,518.3–5,697,350.5) in 2021. Furthermore, the global ASDR of encephalitis showed a significant downward trend from 105.5 cases per 100,000 population (95% UI: 83.4–120) in 1990 to 67.4 cases per 100,000 persons (95% UI: 54.9–78.4) in 2021. The EAPC of ASDR was −1.65 (95% CI: −1.79– −1.49) within the same period (Supplementary Table S1; Figure 1).

**Table 1:** The ASPR and ASIR of encephalitis in 1990 and 2021, and changes from 1990 to 2021 at the global level and different regions.

Characteristics	ASPR per 100,000 (95% UI)		EAPC (95% CI)	ASIR per 100,000 (95% UI)		EAPC (95% CI)
	1990	2021		1990	2021	
Global	89.3 (59.4–117.9)	57.3 (40.2–73.9)	−1.69 (−1.80 to −1.58)	24.5 (22.3–27.3)	19.7 (18.0–21.8)	−0.96 (−1.06 to −0.86)
Sex						
Male	93.6 (61.7–123.7)	59.9 (41.7–77.7)	−1.11 (−1.13 to −1.09)	26.0 (23.7–28.8)	21.2 (19.4–23.5)	−0.16 (−0.17 to −0.16)
Female	85.5 (57.4–112.8)	54.7 (38.7–70.3)	−1.01 (−1.03 to −0.99)	23.2 (21.1–25.9)	18.2 (16.5–20.3)	−0.17 (−0.17 to −0.16)
SDI						
High SDI	12.8 (9.1–16.4)	10 (7.3–12.7)	−0.53 (−0.66 to −0.40)	4.9 (4.2–5.7)	4.5 (4.0–5.2)	−0.10 (−0.17 to −0.04)
High-middle SDI	54.0 (37.2–70.3)	34.9 (24.9–44.3)	−1.37 (−1.43 to −1.30)	14.4 (12.7–16.5)	12.9 (11.5–14.8)	−0.36 (−0.41 to −0.32)
Middle SDI	106.5 (71.6–139.9)	60.8 (43.0–78.0)	−2.00 (−2.09 to −1.92)	25.1 (22.7–28.1)	21.2 (19.4–23.7)	−0.75 (−0.84 to −0.67)
Low-middle SDI	170.7 (109.5–227.6)	92.4 (63.2–120.6)	−2.37 (−2.54 to −2.19)	47.3 (43.7–51.4)	32.5 (30.2–35.3)	−1.52 (−1.65 to −1.38)
Low SDI	119.6 (75.0–160.9)	82.6 (55.6–107.4)	−1.62 (−1.79 to −1.46)	31.4 (28.8–34.3)	23.2 (21.4–25.2)	−1.19 (−1.28 to −1.10)
Regions						
Andean Latin America	36.1 (24.3–47.6)	26 (18.5–33.1)	−1.31 (−1.45 to −1.17)	11.4 (10.4–12.6)	9.6 (8.7–10.6)	−0.72 (−0.80 to −0.65)
Australasia	2.1 (1.5–2.7)	1.9 (1.4–2.4)	−0.29 (−0.36 to −0.22)	1.1 (0.9–1.2)	1.4 (1.2–1.5)	0.91 (0.79 to 1.04)
Caribbean	48.5 (33.2–63.4)	37.7 (26.2–48.9)	−0.96 (−1.11 to −0.81)	14.4 (12.8–16.2)	11.8 (10.5–13.4)	−0.56 (−0.68 to −0.44)
Central Asia	43.3 (29.9–56.0)	36.4 (25.7–46.4)	−1.16 (−1.42 to −0.91)	14.7 (13.1–16.2)	13.8 (12.6–15.2)	−0.28 (−0.37 to −0.18)
Central Europe	17.9 (12.5–22.9)	10.6 (7.6–13.4)	−1.86 (−1.98 to −1.75)	6.9 (6.1–7.8)	5.1 (4.5–5.8)	−0.99 (−1.06 to −0.93)
Central Latin America	51.1 (35.2–66.7)	44.6 (31.6–57.3)	−0.80 (−0.91 to −0.69)	15.7 (13.7–18.2)	14.6 (12.7–16.9)	−0.53 (−0.61 to −0.44)
Central Sub-Saharan Africa	36.4 (22.6–49.2)	38.5 (25.4–52)	−0.10 (0.042 to 0.06)	9.9 (8.6–11.3)	9.1 (7.9–10.4)	−0.26 (−0.30 to −0.23)
East Asia	106 (71.7–139.5)	59.4 (42.5–75.6)	−1.65 (−1.74 to −1.55)	20.8 (18.1–24.2)	18.9 (16.6–22.0)	−0.11 (−0.22 to −0.01)
Eastern Europe	26.9 (18.6–34.6)	22.9 (16–29.4)	−0.96 (−1.15 to −0.78)	11.8 (10.6–13.1)	10.7 (9.6–11.8)	−0.38 (−0.44 to −0.33)
Eastern Sub-Saharan Africa	47.6 (29.3–64.1)	41.6 (27.6–54.6)	−0.48 (−0.52 to −0.44)	12.5 (11.2–13.9)	11.5 (10.3–12.8)	−0.27 (−0.29 to −0.25)
High-income Asia Pacific	19.1 (13.6–24.6)	15.4 (11.1–19.7)	−0.21 (−0.46 to 0.03)	6.9 (5.8–8.3)	6.6 (5.6–7.9)	0.13 (−0.03 to 0.29)
High-income North America	2.8 (2.0–3.7)	2 (1.4–2.6)	−1.52 (−1.73 to −1.30)	1.8 (1.5–2.1)	1.7 (1.5–1.8)	−0.40 (−0.49 to −0.31)
North Africa and Middle East	24.5 (16.5–32.3)	21.3 (15–27.3)	−0.72 (−0.81 to −0.63)	8.6 (7.6–9.7)	7.8 (6.9–8.9)	−0.38 (−0.41 to −0.35)
Oceania	42.3 (27.4–56.5)	38.6 (25.8–50.9)	−0.24 (−0.31 to −0.17)	11.5 (10.1–13.4)	11.1 (9.6–12.9)	−0.13 (−0.15 to −0.11)
South Asia	263.9 (170.7–351.2)	140.8 (97.2–183.0)	−2.52 (−2.74 to −2.30)	73.3 (67.9–79.5)	51.3 (47.8–55.7)	−1.53 (−1.70 to −1.36)
Southeast Asia	53.4 (35.6–70.6)	34 (23.6–44)	−1.67 (−1.83 to −1.50)	15.1 (13.4–17.1)	13.0 (11.7–14.6)	−0.65 (−0.73 to −0.56)
Southern Latin America	15.4 (10.7–20.0)	14.8 (10.5–19.0)	0.16 (−0.13 to 0.45)	5.1 (4.4–5.9)	5.8 (5.1–6.6)	0.70 (0.40 to 0.99)
Southern Sub-Saharan Africa	29.4 (19.3–39.1)	25.6 (16.6–34.2)	−0.50 (−0.54 to −0.46)	10.5 (9.2–12.0)	10.0 (8.8–11.3)	−0.21 (−0.33 to −0.09)
Tropical Latin America	20.3 (13.8–26.3)	14.5 (10.2–18.5)	−2.00 (−2.38 to −1.62)	7.0 (6.3–7.8)	5.6 (5.1–6.3)	−1.53 (−1.82 to −1.24)
Western Europe	10 (7.2–12.8)	9.7 (7.1–12.1)	−0.02 (−0.16 to 0.13)	4.5 (3.9–5.1)	4.9 (4.3–5.5)	0.44 (0.34 to 0.53)
Western Sub-Saharan Africa	46.3 (29.6–61.8)	42.6 (28.5–55.9)	−0.45 (−0.51 to −0.38)	15.0 (13.4–16.7)	13.9 (12.5–15.5)	−0.20 (−0.23 to −0.16)

ASIR age-standardized incidence rate, ASPR age-standardized prevalence rate, EAPC estimated annual percentage change, CI confidence interval, UI uncertainty interval.

### Regional level

**Figure 2:**
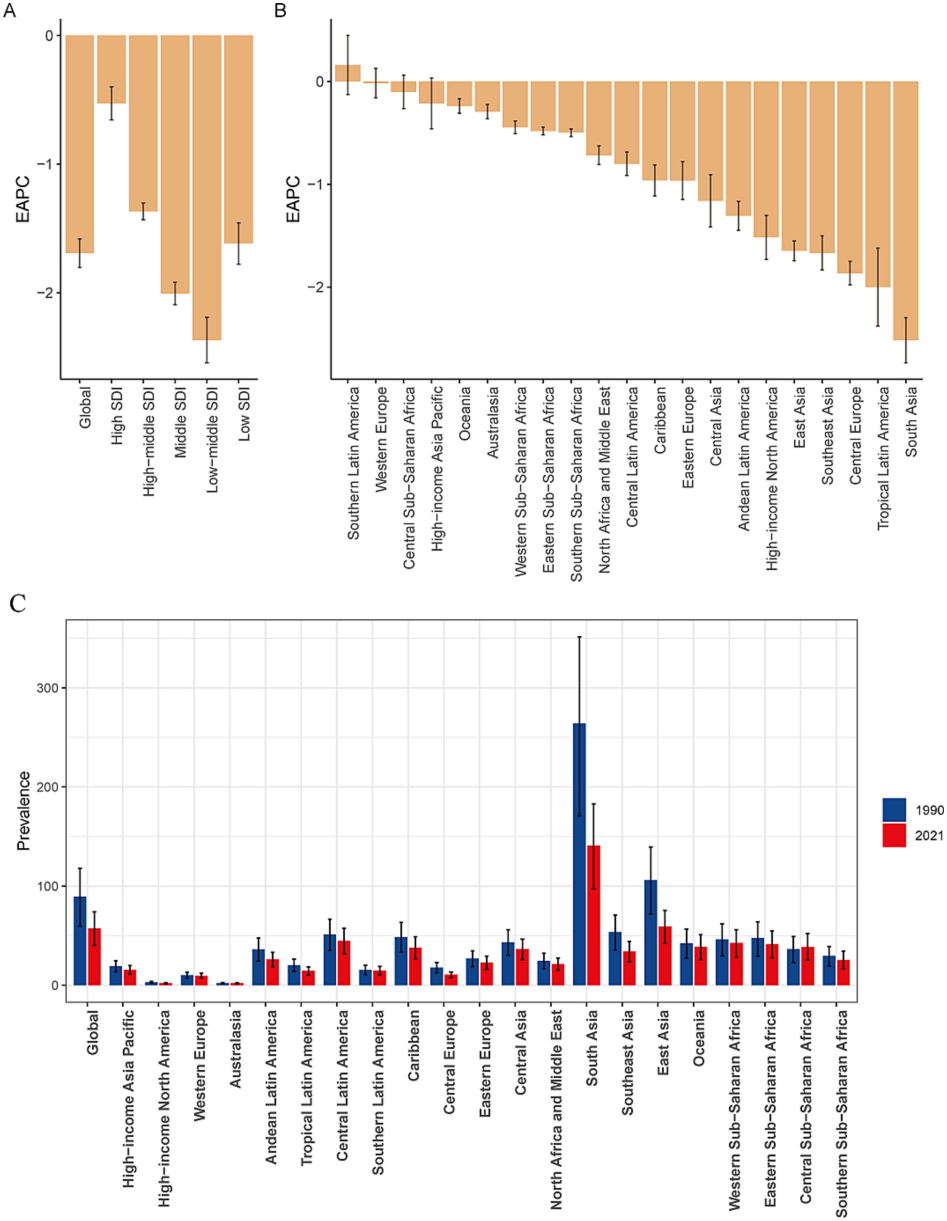
EAPCs in the age-standardized prevalence rates for encephalitis in the SDI quintile **(A)** and in 21 regions **(B)** and the age-standardized prevalence rates of encephalitis for 21 regions in 1990 and 2021 **(C)**. EAPC, estimated annual percentage change; SDI, sociodemographic index.

The global burden of encephalitis showed significant regional differences, which were closely related to the SDI levels. In high-SDI regions, the encephalitis ASPR in 2021 was lowest, at 12.8 cases per 100,000 population (95% UI: 9.1–16.4), while the highest was at 119.6 cases per 100,000 population (95% UI: 75.1–160.9) during the same period. The encephalitis ASPR showed a decreasing trend from 1990 to 2021, and the low-middle-SDI areas showed the largest decrease from 1990 to 2021 (EAPC = −2.37 [95% CI: −2.54– −2.19]), indicating that the encephalitis burden in these regions is decreasing (Figures 1, 2; Table 1). The ASIR similarly demonstrated these regional differences in encephalitis burden. The burden was highest in low-middle-SDI regions, with an ASIR of 32.5 per 100,000 (95% UI: 30.2–35.3) in 2021, whereas the burden was lowest in high-SDI regions, at 4.5 per 100,000 (95% UI: 4.0–5.2). Consistent variations in ASIRs were also observed between SDI regions in 1990, with the largest decreases in low-middle-SDI regions (EAPC = −1.52 [95% CI: −1.65– −1.38]) (Table 1; Figure 1; Supplementary Figure S1). For the ASMR, a decreasing trend was noted in the low-, low-middle-, middle-, and middle-high-SDI regions but increasing trend was only found in the high-SDI regions (EAPC = 0.16 [95% CI: −0.09–0.43]) from 1990 to 2021 (Supplementary Table S1; Figure 1; Supplementary Figure S2). The SDI regions with the highest encephalitis ASDR in 2021 were the low-middle SDI regions (110.5, 95% UI: 89.3–134.0), while the high-SDI regions had the lowest ASDR at 11.1 per 100,000 (95% UI: 10.4–11.9). Across the 5 SDI regions, the encephalitis ASDR decreased in all the regions, with the largest decrease in the high-SDI region (EAPC = −3.1 [95% CI: −3.27– −2.93]) (Supplementary Table S1; Figure 1; Supplementary Figure S3). The higher the SDI, the lower the encephalitis burden, with values observed in the lowest two SDI quintiles being higher than the global rate and values observed in the three highest SDI quintiles being lower than the global rate. The only exception to this result is that the ASPR of the middle-SDI region was higher than the global rate.

Our findings suggest that Asia is the region with the highest prevalence and incidence of encephalitis worldwide. Specifically, within Asia, South Asia had the highest ASPR, at 140.9 per 100,000 (95% UI: 97.2–182.9), followed by East Asia, with an ASPR of 59.4 per 100,000 (95% UI: 42.5–75.6). Central Latin America also showed a high ASPR at 44.6 per 100,000 (95% UI: 31.6–57.3). South Asia and East Asia were also among the regions with particularly high ASIRs worldwide. The ASIR in South Asia was 51.3 per 100,000 (95% UI: 47.8–55.7), while the ASIR in East Asia was 18.9 per 100,000 (95% UI: 16.6–22). In contrast, Australasia spontaneously showed the lowest ASPR at 1.94 per 100,000 (95% UI: 1.40–2.35) and the lowest ASIR at 1.07 per 100,000 (95% UI: 0.93–1.23) (Table 1; Figure 2; Supplementary Figure S4A). Among the 21 GBD regions, the majority (20 GBD regions) experienced a decrease in the encephalitis ASPR, with 81.0% (17 regions) experiencing a decrease in the encephalitis ASIR between 1990 and 2021, with the largest decrease in ASIR occurring in South Asia (EAPC = −1.53 [95% CI: −1.70– −1.36]). The regions with the largest decreases in ASPR were in South Asia (EAPC = −2.52 [95% CI: −2.74– −2.29]), Tropical Latin America (EAPC = −2.00 [95% CI: −2.38– −1.62]), and Central Europe (EAPC = −1.86 [95% CI: −1.98– −1.75]). The ASPR increased substantially in Southern Latin America, with an EAPC of 0.16 (95% CI: −0.13–0.45), and Australasia had the largest increase in the ASIR, with an EAPC of 0.91 (95% CI: 0.79–1.04) (Table 1; Supplementary Figures S1, S5A). The high incidence and prevalence of diseases in South Asia may be related to genetic susceptibility, geographical and climatic factors, and limited access to high-quality health care opportunities.

**Figure 3:**
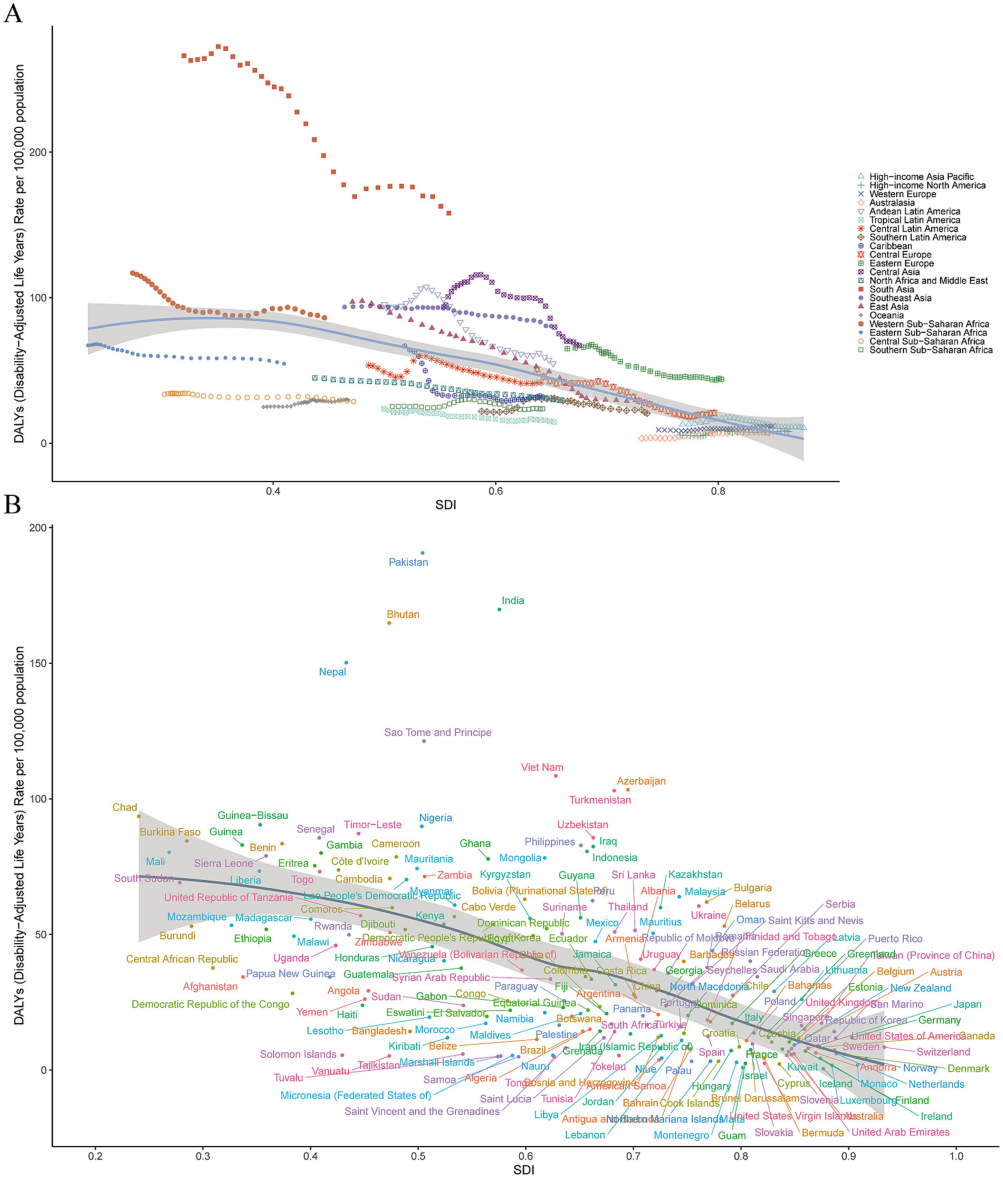
**(A)** Age-standardized DALYs rates of encephalitis for 21 regions by SDI from 1990 to 2021. The expected values based on the SDI and disease rates at all of the locations are shown as black lines. **(B)** Age-standardized DALYs rates for encephalitis in 204 countries and territories by SDI in 2021. Expected values based on the sociodemographic index and disease rate at all of the locations are shown as black lines. SDI, sociodemographic index.

In 2021, South Asia (158.1 [95% UI: 127.9–196.7]), Western Sub-Saharan Africa (86.1 [95% UI: 61.2–107.8]), and Southeast Asia (80.4 [95% UI: 52.0–99.5]) had the highest ASDRs of encephalitis per 100,000 people, whereas Australasia (7.20 [95% UI: 6.56–7.87]), high-income North America (8.04 [95% UI: 7.65–8.45]), and high-income Asia Pacific (10.43 [95% UI: 9.54–11.42]) had the lowest rates. From 1990 to 2021, the encephalitis ASDR decreased in 17 of 21 regions, with the largest decrease in East Asia (EAPC = −4.39 [95% CI: −4.71–−4.06]), Central Europe (EAPC = −3.46 [95% CI: −3.85– −3.07]), and the Caribbean (EAPC = −3.10 [95% CI: −3.27– −2.93]) (Supplementary Table S1; Supplementary Figure S3; Figure 3A). South Asia remained the super-region with the highest ASMR at 3.86 per 100,000 (95% UI: 3.18–4.71) in 2021. The largest increase in ASMRs by region from 1990 to 2021 was in Australasia (EAPC = 3.56 [95% CI: 2.99–4.13]), followed by high-income North America, Western Europe and Oceania (Supplementary Table S1; Supplementary Figures S2, S6A). These findings highlight the complexity and diversity of the encephalitis epidemiology in different regions.

### National level

**Figure 4:**
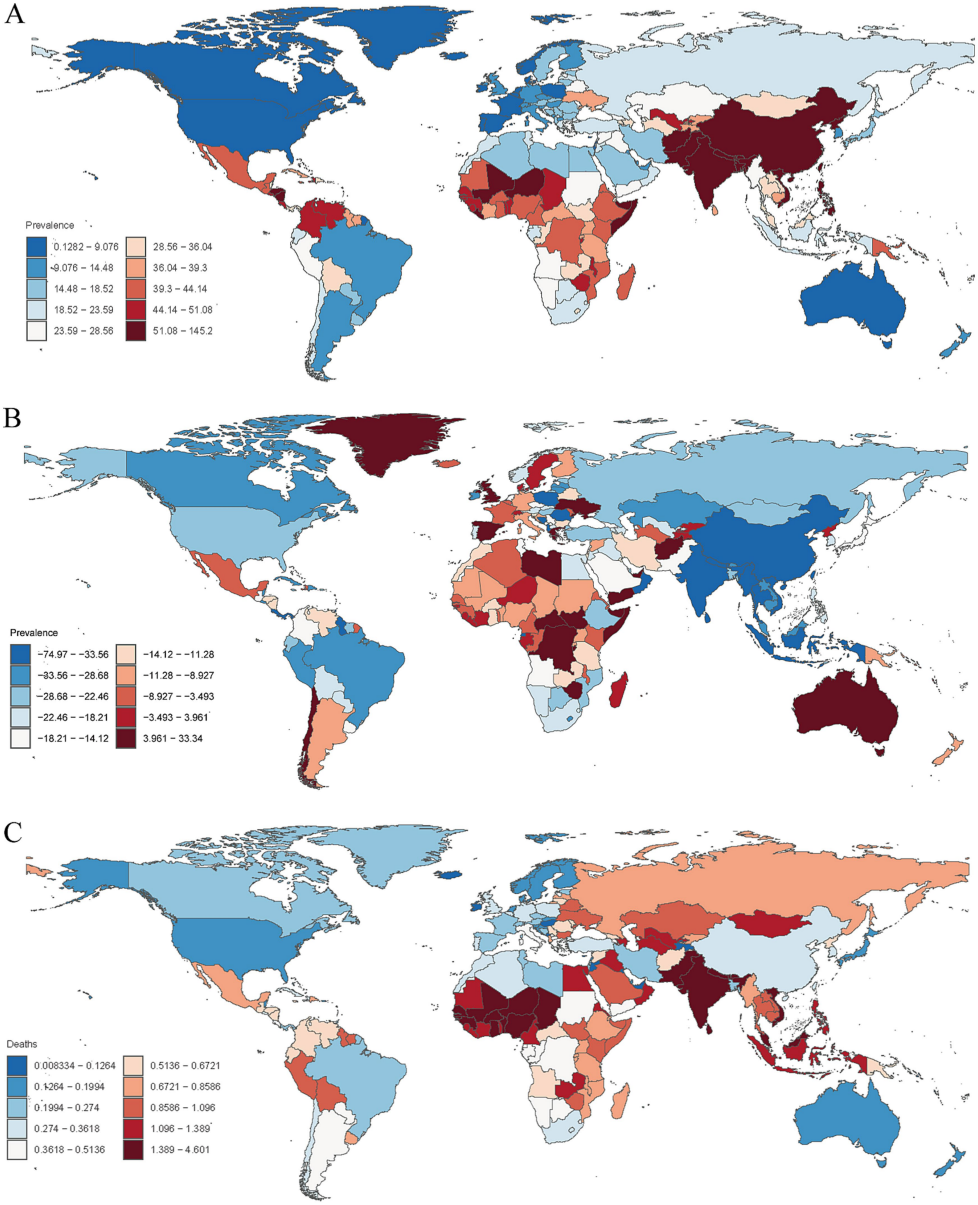
**(A)** Age-standardized prevalence rates of encephalitis per 100,000 people in 2021 by country. **(B)** Percentage change in prevalent cases across 204 countries and territories in 1990 and 2021. **(C)** Age-standardized mortality rates of encephalitis per 100,000 people in 2021 by country.

**Figure 5:**
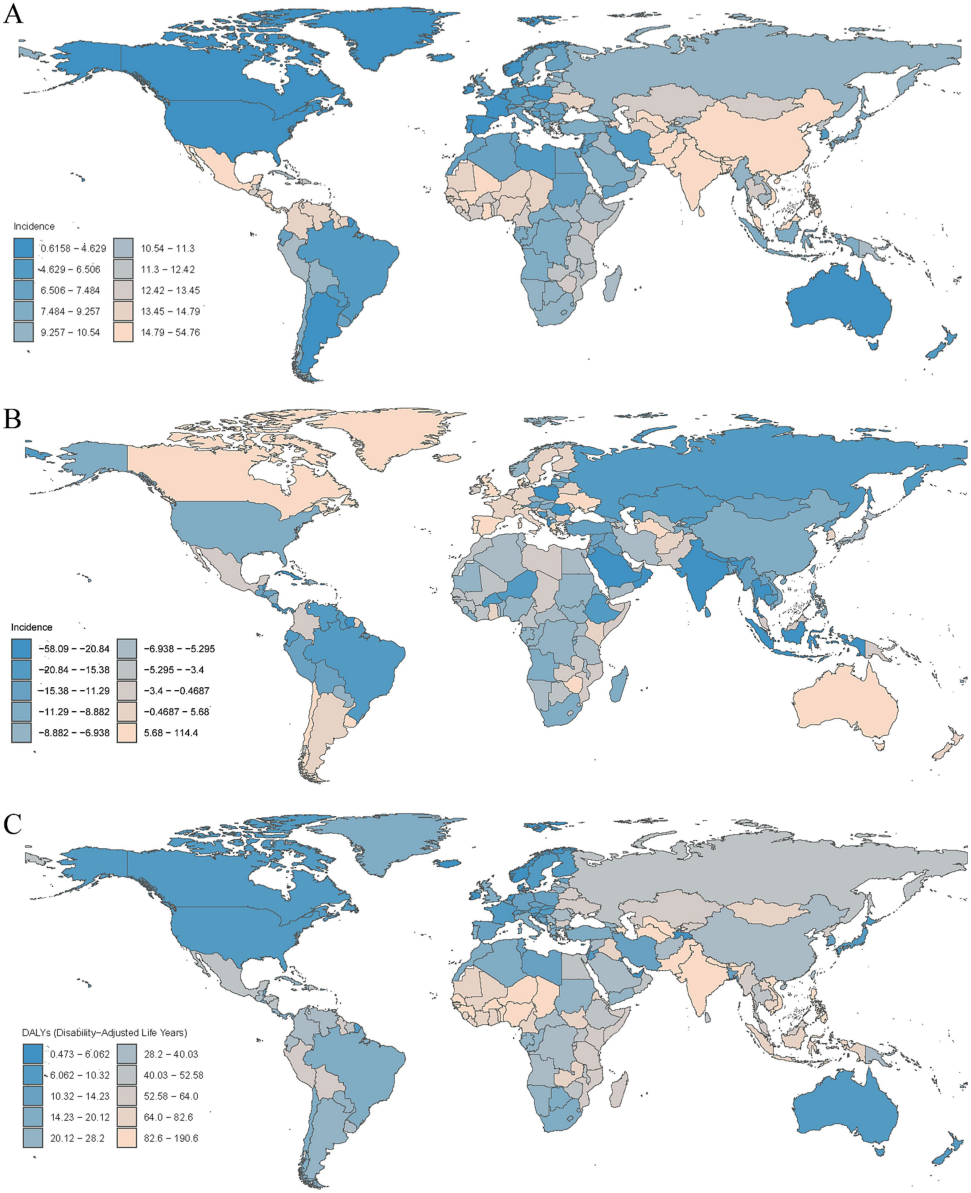
**(A)** Age-standardized incidence rates of encephalitis per 100,000 people in 2021 by country. **(B)** Percentage change in incidence cases across 204 countries and territories in 1990 and 2021. **(C)** Age-standardized DALYs rates of encephalitis per 100,000 people in 2021 by country.

In 2021, the encephalitis ASPR varied widely across countries. The countries with highest incidence were Pakistan (145.2 cases per 100,000 population [95% UI: 97.9–190.5]), India (143.7 cases per 100,000 population [95% UI: 99.3–186.3]), and Nepal (138.2 cases per 100,000 population [95% UI: 96.9–177.6]), and the countries with the lowest rates were Greenland (0.49 cases per 100,000 population [95% UI: 0.35–0.63]), Australia (0.30 cases per 100,000 population [95% UI: 0.21–0.37]), and Canada (0.13 cases per 100,000 population [95% UI: 0.09–0.17]) (Supplementary Table S2; Figure 4A; Supplementary Figure S4B). Notably, the three countries with the highest ASPR were all located in South Asia. This finding is consistent with our regional analysis, which identified South Asian as the region with the highest prevalence of encephalitis worldwide. The consistency between country-level and regional data highlights the enormous challenge that ischemic stroke poses to South Asian countries. Analyzing the 2021 ASIR data due to encephalitis, we observed clear consistency in the most affected countries. For example, Pakistan (54.8 per 100,000 [95% UI: 550.7–59.6]), India (53.5 per 100,000 [95% UI: 49.8–57.8]), and Nepal (52.4 per 100,000 [95% UI: 48.5–56.8]) were significantly affected in terms of ASIR. Among all countries, the lowest ASIRs were found in Canada (0.62 per 100,000 [95% UI: 0.54–0.71]), Australia (0.63 per 100,000 [95% UI: 0.55–0.72]), and Iceland (0.72 per 100,000 [95% UI: 0.58–0.87]) (Supplementary Table S3; Figure 5A; Supplementary Figure S5B). The ASDR for encephalitis ranged from approximately 0.47 to 190.6 per 100,000 individuals. Among all countries, the highest ASDRs were in Pakistan (190.6 per 100,000 [95% UI: 100.9–268.2]), India (169.8 per 100,000 [95% UI: 136.3–216.9]), and Nepal (164.8 per 100,000 [95% UI: 89.5–237.7]), whereas the lowest rates were in Luxembourg (1.73 per 100,000 [95% UI: 1.47–2.02]), Malta (0.89 per 100,000 [95% UI: 0.75–1.05]) and Iceland (0.47 per 100,000 [95% UI: 0.42–0.54]) (Supplementary Table S4; Figures 3B, 5C). The high overlap between the ASPR and ASIR analysis results indicates a high burden of encephalitis in South Asia. Notably, Pakistan had the highest ASPR, ASIR, and ASDR, reflecting the high burden of encephalitis in Pakistan and its impact on overall health-related quality of life.

Encephalitis burden and disease trends varied widely across the world. The countries with the largest decreases in ASPR for encephalitis were Poland (EAPC = −4.75 [95% CI: −5.22– −4.27]), India (EAPC = −2.85 [95% CI: −3.13–−2.58]), and Equatorial Guinea (EAPC = −2.79 [95% CI: −3.51–−2.07]), whereas the largest increases in ASPR for encephalitis were Spain (EAPC = 1.23 [95% CI: 0.74–1.73]), Zimbabwe (EAPC = 1.11 [95% CI: 0.92–1.31]), and Libya (EAPC = 0.99 [95% CI: 0.69–1.28]) (Supplementary Table S2; Supplementary Figure S7). Among all countries, 37 countries or regions showed an increase in encephalitis ASIR, with the largest increase in Australia (EAPC = 2.86 [95% CI: 2.42–3.30]), followed by Bahrain (EAPC = 2.24 [95% CI: 1.82–2.66]) and Spain (EAPC = 1.54 [95% CI: 1.14–1.94]). Among countries with an increase in encephalitis ASIR, the largest decreases were in Poland (EAPC = −2.99 [95% CI: −3.37–−2.62]), Oman (EAPC = −2.89 [95% CI: −3.24–−2.54]), and India (EAPC = −1.81 [95% CI: −2.03–−1.59]) (Supplementary Table S3; Supplementary Figure S8). Among the top three countries or regions, ASDRs increased significantly from 1990 to 2021 in New Zealand (EAPC = 5.77 [95% CI: 4.53–7.02]), Greece (EAPC = 4.75 [95% CI: 3.35–6.17]), and the United Kingdom (EAPC = 4.31 [95% CI: 3.99–4.62]), whereas the lowest rates were observed in Belize (EAPC = −7.13 [95% CI: −8.04–−6.21]), Trinidad and Tobago (EAPC = −6.58 [95% CI: −7.06–−6.10]) and Malta (EAPC = −6.08 [95% CI: −7.89–−4.24]) (Supplementary Table S4; Supplementary Figure S9).

The trends of the ASMRs varied widely across the 204 countries and territories. In 2021, ASMR was highest in Pakistan (4.60 per 100,000 [95% UI: 2.25–6.48]), and the lowest in the United States Virgin Islands (0.008 per 100,000 [95% UI: 0.005–0.010]). The largest decrease in ASMR due to encephalitis was in Belize (EAPC = −7.75 [95% CI: −8.80–−6.78]), while the largest increase was in New Zealand (EAPC = 8.23 [95% CI: 6.44–10.06]) (Supplementary Table S5; Figure 4C; Supplementary Figures S6B, S10).

The changes in encephalitis incidence varied significantly among 204 countries and territories. From 1990 to 2021, the largest increases in the incidence of encephalitis were observed in Australia (114.4%), Canada (98.1%), and Spain (49.9%), whereas the largest decreases were observed in Poland (−58.1%), Oman (−52.6%), and Saint Kitts and Nevis (−35.2%) (Supplementary Table S3; Figure 5B). Similarly, the most pronounced increases in the prevalence of encephalitis were observed in Libya (33.34%), followed by Zimbabwe (26.67%) and Spain (24.42%), whereas the most pronounced decreases were detected in Poland (−74.97%), Equatorial Guinea (−51.06%) and India (−50.39%) (Supplementary Table S2; Figure 4B).

### Age and sex patterns

**Figure 6:**
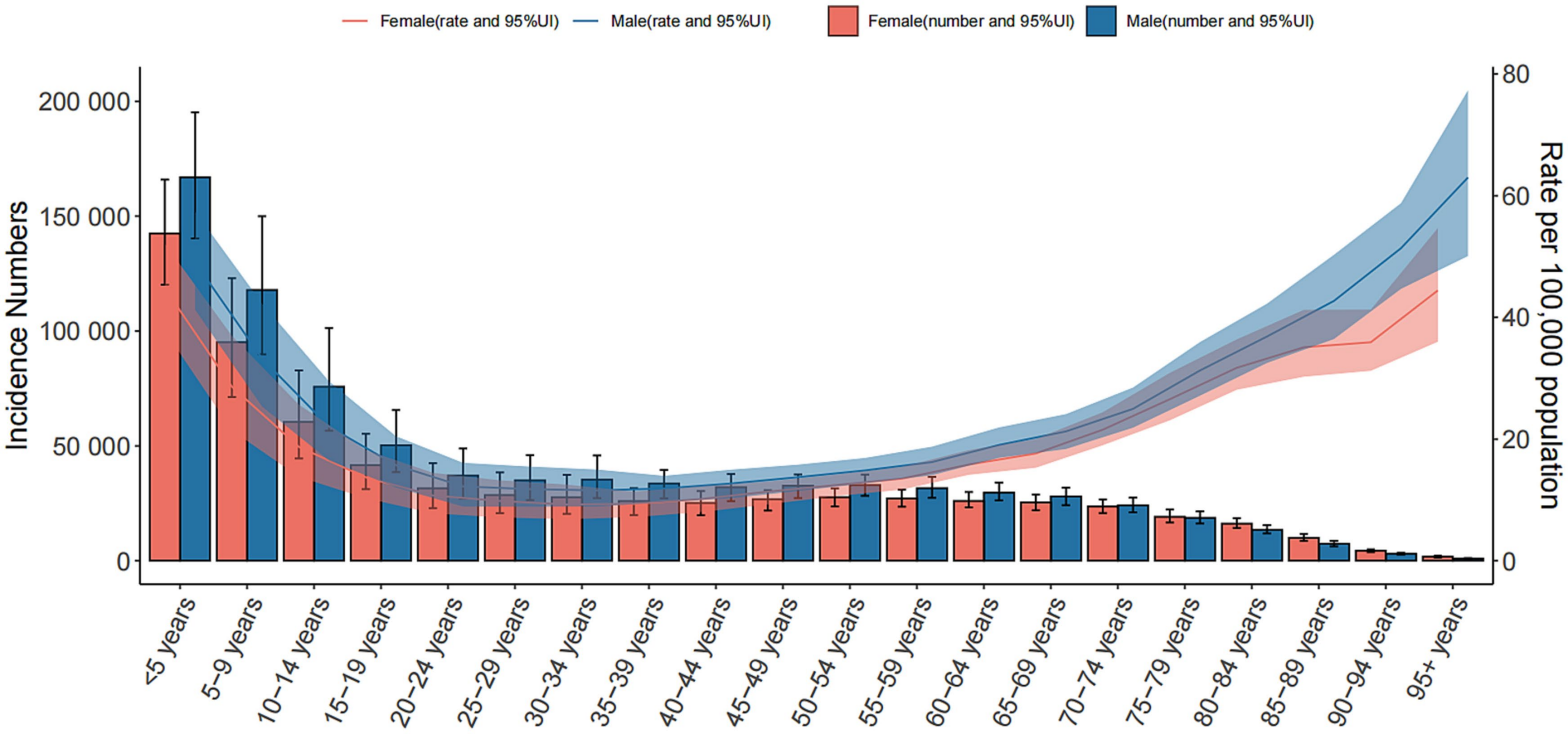
Global cases and age-standardized incidence rates of encephalitis per 100,000 people by age and sex in 2021. Shading indicates the upper and lower limits of the 95% uncertainty intervals.

In 2021, the prevalence of encephalitis increased with age, peaked at 30–34 years old, and then declined. The incidence rate in males aged 5–59 years old was higher than that in females, but as age increased, the incidence rate in females in the same age group was always higher than that in males. The incidence rate of encephalitis decreased with age, and the highest incidence was found in people under 5 years old. The most encephalitis related deaths occurred in older individuals and children under 5 years old, followed by people aged 70–74 years old. Among all age groups, people under 5 years old had the highest DALYs and ASDRs. There was no significant difference in ASDR and ASMR between females and males, while the ASIR and incident cases in males of all age groups were higher than those in females. Furthermore, males under 54 years old had higher DALYs than females, while males in the age group of 55 years old and above had lower DALYs than females (Figure 6; Supplementary Figure S11).

### Future forecasts of the global burden of encephalitis

**Figure 7:**
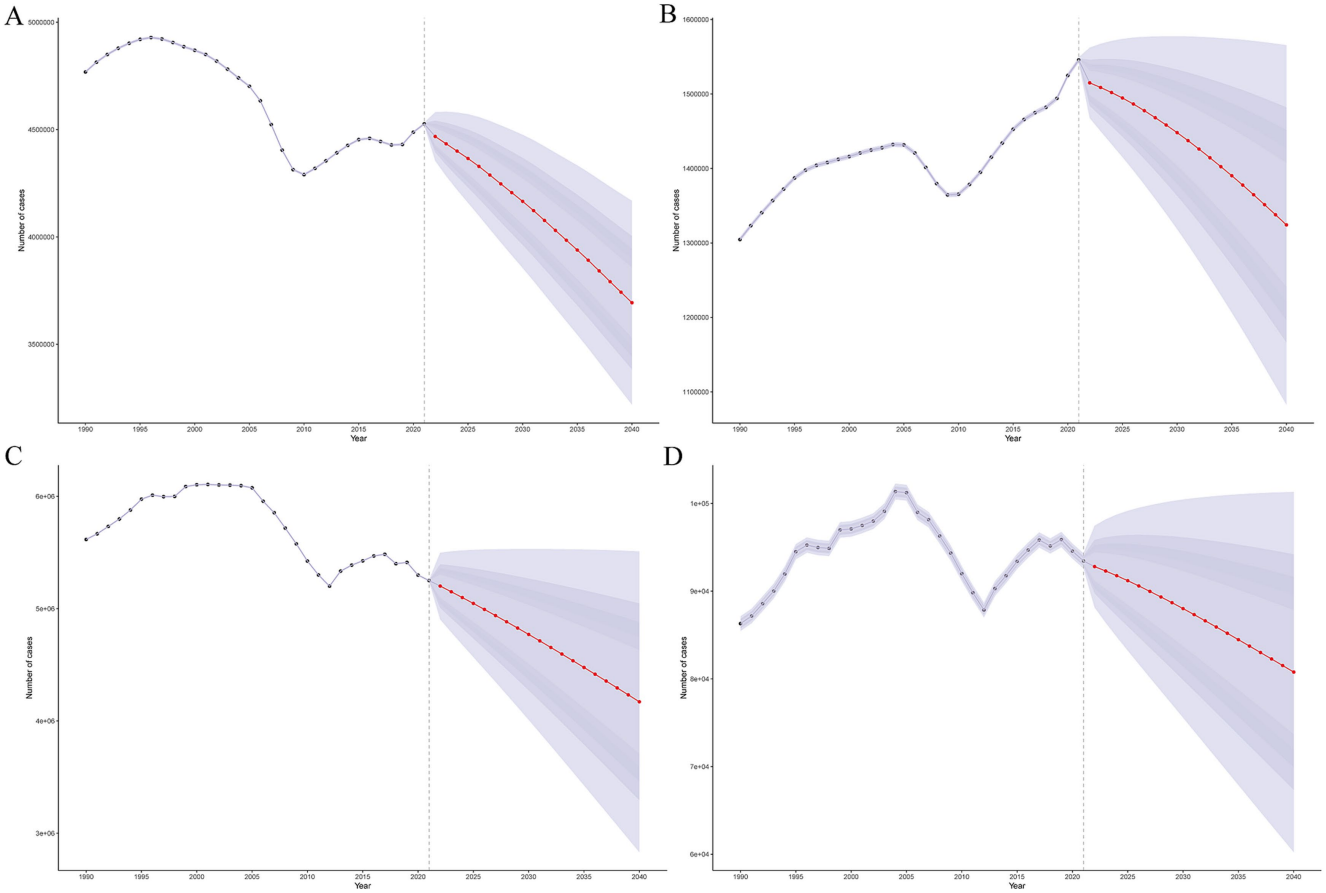
Future forecasts of the global burden of encephalitis. **(A)** Prevalence cases. **(B)** Incidence cases. **(C)** DALYs. **(D)** Cases of death.

The global burden of encephalitis is expected to undergo significant changes from 2021 to 2040, with different trends in different age groups. The ASPR for encephalitis is expected to decrease in all age groups, from approximately 55 cases per 100,000 population in 2021 to approximately 43 cases per 100,000 in 2040, an approximately 20% decrease over two decades (Supplementary Figure S12A). The prevalence of encephalitis is also expected to decrease from approximately 4.4 million in 2021 to approximately 3.7 million in 2040 (Figure 7A). The global encephalitis ASIR is expected to show a slightly decreasing trend, with the ASIR projected to decrease from approximately 18 cases per 100,000 population in 2021 to approximately 15 cases per 100,000 in 2040 (Supplementary Figure S12B). Additionally, the number of encephalitis cases is also projected to decrease from approximately 1.5 million in 2021 to approximately 1.3 million in 2040 (Figure 7B). From 2021 to 2040, the encephalitis ASMR is projected to decrease from approximately 1.2 cases per 100,000 population to approximately 0.9 cases per 100,000 population, and the number of deaths is projected to decrease from 9.1 million to approximately 1.3 million during the same period (Figure 7D; Supplementary Figure S12D). For encephalitis DALY, both the numbers and age-standardized rates are expected to show a sharp decline globally, with DALYs decreasing from approximately 5.2 per million in 2021 to 4.2 per million in 2040 (Figure 7C) and the ASDR decreasing from approximately 61 per 100,000 in 2021 to approximately 42 per 100,000 in 2040 (Supplementary Figure S12C). The trends in the projected future burden of disease varied by age group but are generally downward (Supplementary Figures S13–S20).

### Burden differences within each continent

To visualize the differences in encephalitis burden among countries within each continent, a scatter plot is used. First, define the x-axis as a metric of national economic status (e.g., SDI) and the y-axis as the encephalitis burden indicator (e.g., DALYs in 2021). Next, group countries by their respective continents (Asia, Europe, Africa, Oceania, North America, South America) (Supplementary Figures S21–S26) and plot each country as a data point, enhancing the interpretability of cross-country variations within continents.

## Discussion

### Persistent disparities and systemic drivers

The 2021 GBD study underscores persistent global inequities in encephalitis burden, despite declining age-standardized rates. While high-SDI regions benefit from advanced diagnostics (e.g., metagenomic sequencing) and robust immunization programs, low-middle SDI regions—particularly South Asia, Western Sub-Saharan Africa, and Central Latin America—remain disproportionately affected (20, 21).

Consistent with the decreasing trend in the global encephalitis burden between 1990 and 2021, the current study revealed a reduced burden in most regions. However, at the regional level, the burden of encephalitis continues to exist with socioeconomic disparities, and our analysis indicated that the highest ASPR, ASIR, ASDR, and ASMR of encephalitis were observed in South Asia, even though a substantial reduction in all these rates was observed. This disparity can be primarily attributed to a combination of factors, including limited public health policies, inadequate public health awareness, insufficient secondary prevention and primary care, climate change, genetic predispositions, and the following:

Diagnostic Gaps: In South Asia, over 70% of healthcare facilities lack capacity for autoantibody testing or HSV PCR, delaying pathogen-specific treatment initiation (22–24). A 2023 WHO guideline emphasizes that delayed HSV detection (>6 h) increases mortality by 30% in LMICs, which aligns with our finding that regions with limited laboratory infrastructure have higher DALYs.

Vaccine Accessibility: Despite JE vaccine efficacy exceeding 85%, coverage in rural India and Nepal remains below 50% due to logistical barriers (25), perpetuating a heavy burden among children. Similarly, measles and varicella vaccination gaps persist in conflict-affected African regions (26, 27).

Critical Care Deficits: LMICs report 2.5-fold higher encephalitis mortality than HICs (28), driven by limited ICU beds (e.g., Nigeria: 0.3 ICU beds/100,000 population) and scarce post-discharge rehabilitation services (29–31).

After analyzing the association between SDI and encephalitis burden, we observed that age-standardized rates of prevalence, incidence, DALYs, and deaths were significantly higher in low-middle- and low-SDI regions than in higher-SDI regions, with particularly pronounced disparities in South Asia, Western Sub-Saharan Africa, and Central Latin America. Several factors may explain this trend in the lower-SDI regions. In higher-SDI regions, lower ASDR and ASMR levels are mainly driven by improved specific treatments and preventive initiatives via vaccination (32). One previous study reported that coverage of intravenous (IV) acyclovir for HSV encephalitis and varicella zoster virus encephalitis within 6 h of admission remains low in LMICs (33). Low-middle- and low-SDI regions often face issues such as inadequate basic health facilities, insufficient drug production capacity, lack of proper drug transportation and storage conditions, and excessive or inappropriate medication prescriptions (34–36). In LMICs, the higher ASPR and ASIR were partially due to the administration of steroids, which reactogenically inactivated mouse brain-derived vaccines, in contrast to the effects of newer Japanese encephalitis (JE) vaccines (35). In Asia and Africa, where high ASMRs and ASDRs were observed, varicella zoster vaccination and measles-containing vaccines are only available for HICs or large tertiary referral centers, which may represent a major opportunity to reduce the prevalence rate and incidence rate of subacute sclerosing panencephalitis (21). Finally, improvements in monitoring and reporting vector-borne disease cases have also helped reduce encephalitis mortality in HICs (20).

### Climate-ecological drivers of vector-borne encephalitis

Our analysis acknowledges the need to expand on climate-ecological drivers of vector-borne encephalitis. Rising temperatures in South Asia, for instance, have extended the geographic range of Culex mosquitoes—vectors for Japanese encephalitis—by 15% since 2010, correlating with a 22% increase in rural cases (37). Similarly, dengue-associated encephalitis outbreaks in Central America now align with El Niño-induced rainfall patterns, with 60% of annual cases clustering in post-flood regions. Deforestation in sub-Saharan Africa has disrupted ecological balances, bringing human settlements closer to zoonotic reservoirs like fruit bats (hosts for Nipah virus), increasing spillover events by 37% in deforested zones (38). These linkages highlight that climate-sensitive encephalitis transmission depends on interconnected factors: temperature thresholds for vector survival, land-use changes, and extreme weather frequency. Integrating such data into predictive models could enhance regional preparedness, making policy recommendations more responsive to climate-driven epidemiological shifts.

### Known/possible factors associated with encephalitis or infectious diseases

Pathogens drive encephalitis and other infectious diseases through direct invasion: they adhere to host cells, proliferate, and spread to target tissues, inducing inflammation and tissue damage via cytotoxic effects or immune activation. Beyond such direct pathogenic invasion, encephalitis and infectious diseases are shaped by a complex interplay of host, pathogen, and environmental factors that modulate susceptibility, severity, and outcomes. Host immune status is a pivotal determinant: individuals with immunodeficiency—whether congenital, iatrogenic (e.g., due to immunosuppressive therapy), or acquired (e.g., HIV/AIDS)—exhibit heightened vulnerability, as impaired innate and adaptive responses fail to contain pathogens, increasing the risk of severe encephalitic complications. Genetic predispositions further influence disease progression; polymorphisms in genes encoding pattern recognition receptors or cytokine regulators can alter immune signaling, rendering certain populations more susceptible to hyperinflammation or inadequate pathogen clearance.

Pathogen-specific characteristics also play critical roles. Neurotropic viruses (e.g., herpes simplex virus, West Nile virus) exploit neural pathways for dissemination, while bacterial pathogens (e.g., *Streptococcus pneumoniae*) can induce secondary encephalitis via toxin-mediated neuroinflammation or immune cross-reactivity. Antimicrobial resistance exacerbates these challenges, limiting therapeutic options and prolonging the course of infection. In resource-constrained settings, limited access to timely diagnostic tools and healthcare delays therapeutic intervention, elevating the risk of chronic sequelae or mortality.

Collectively, these interconnected factors highlight the need for multidisciplinary strategies to mitigate infectious disease burdens.

### COVID-19 exacerbation of inequities

The pandemic intensified pre-existing gaps. In South Asia and Latin America, healthcare diversion reduced encephalitis admissions by 40% in 2020–2021, delaying diagnoses and increasing severe sequelae (12). Lockdowns disrupted JE vaccination campaigns in Pakistan, leading to a 15% coverage drop, while delayed empiric acyclovir use in LMICs worsened outcomes. Conversely, high-SDI regions like Australia saw increased encephalitis detection (+114% incidence since 1990) due to expanded next-generation sequencing (NGS) during the pandemic, highlighting detection bias rather than true epidemiological shifts (39, 40).

### Targeted interventions for equity

Additionally, India, the world’s most populous country, exhibits significant regional disparities in access to effective medical interventions and protection against infectious diseases. Socioeconomic gap and inadequate access to essential health services and care may exacerbate India’s encephalitis burden (41). Effective medical interventions can reduce DALYs and mortality among encephalitis patients, underscoring the need to expand coverage of first-line immunotherapies in LMICs. In South Asia, future public health policies should address critical deficiencies in encephalitis prevention and management, while emphasizing the importance of environmental governance. At the national level, 2021 data showed that South Asia, Western Sub-Saharan Africa, Central Asia, and Andean Latin America bore the highest encephalitis burdens, whereas high-income North America and high-income Asia Pacific had the lowest (41). These disparities reflect not only variations in antibacterial and antiviral therapies but also the impact of post-hospitalization care. Countries in these high-burden regions often face challenges such as scarce medical resources and inadequate health service coverage.

To address these challenges, context-specific strategies are critical:

South Asia: Scale JE vaccination via mobile clinics in endemic rural areas (targeting 80% coverage by 2025) (35), coupled with syndromic PCR panels for cost-effective pathogen identification.

Sub-Saharan Africa: Establish regional diagnostic hubs (e.g., Nigeria, DRC) with WHO support to improve HSV and arbovirus testing capacity, addressing zoonotic risks (23).

Global Collaboration: Integrate encephalitis surveillance into climate-resilient health systems to monitor vector-borne disease shifts linked to warming, particularly in South Asia and Africa where JE and dengue are climate-sensitive.

### Future directions in a post-pandemic era

Post-COVID recovery efforts must prioritize building resilience against neuroinfectious diseases. Strengthening LMIC vaccine supply chains and training frontline workers in empiric acyclovir use could avert 23% of pediatric deaths. Telehealth platforms may help bridge gaps in post-discharge care in remote areas, while antimicrobial stewardship programs can mitigate misuse in empiric treatment. Critically, 78% of encephalitis-related health inequalities correlate with SDI parameters, underscoring the need for cross-sectoral policies addressing poverty, education, and healthcare access (32). In the post-COVID era, health systems have adapted to enhance responses to neuroinfectious diseases. In India, upgraded regional laboratory networks have reduced the diagnosis time for HSV encephalitis from 72 to 24 h (42). Kenya’s mobile health units have increased pediatric vaccine coverage by 18% in remote areas (43). Brazil’s tele-neurology platforms now reach 80% of primary hospitals, boosting post-discharge follow-up rates to 65% and reducing sequelae (44). These advancements—driven by pandemic-era resource pooling and technological integration—demonstrate growing resilience in global encephalitis management.

### Limitations

This research has several limitations. First, the methodological limitations of the GBD 2021 study may have introduced bias, and the accuracy of our results depended on the quality of GBD data. However, data imperfections—including poor quality and incomplete registration in some regions (particularly less developed ones)—could affect the accuracy of our findings; thus, the analysis results should be interpreted with caution. Second, although our prediction model incorporated future events, it may not have accounted for multiple risk factors associated with encephalitis risk. Third, as previously noted, encephalitis can present with severe symptoms over a short period and has a high mortality rate. Consequently, patients unable to access timely, effective medical care may go undiagnosed, potentially leading to an underestimation of the encephalitis burden. Finally, subtyping encephalitis based on bacteriological, mycological, and parasitological examinations is a critical parameter. Due to data scarcity, these clinical pathogenic characteristics were not included in the current study. To further expand the analysis of encephalitis burden, additional health surveys and follow-up studies are needed in the future.

## Conclusion

Despite declining global age-standardized rates, encephalitis remains a critical public health challenge, with marked regional disparities in mortality, prevalence, and DALYs. These inequities underscore the need for sustained surveillance systems, research into modifiable risk factors, and multilevel interventions (policy and research-driven) to optimize region-specific prevention and treatment strategies. Furthermore, improvements in curative and preventative interventions for encephalitis are needed across all regions and countries—at both the administrative and academic levels—based on specific pathogens responsible for outbreaks.

## Data Availability

Publicly available datasets were analyzed in this study. This data can be found here: the datasets analysed during the current study are available in the [Global Burden of Disease Study] repository, (https://ghdx.healthdata.org/gbd-2021/sources).

## Supplementary material

SUPPLEMENTARY FIGURE S1 EAPCs in the age-standardized incidence rates for encephalitis in the SDI quintile **(A)** and in 21 regions **(B)** and the age-standardized incidence rates for encephalitis in 21 regions in 1990 and 2021 **(C)**. EAPC, estimated annual percentage change; SDI, sociodemographic index.

SUPPLEMENTARY FIGURE S2 EAPCs in the age-standardized mortality rates for encephalitis in the SDI quintile **(A)** and in 21 regions **(B)** and the age-standardized mortality rates for encephalitis in 21 regions in 1990 and 2021 **(C)**. EAPC, estimated annual percentage change; SDI, sociodemographic index.

SUPPLEMENTARY FIGURE S3 EAPCs in the age-standardized DALYs rates for encephalitis in the SDI quintile **(A)** and in 21 regions **(B)** and the age-standardized DALYs rates for encephalitis in 21 regions in 1990 and 2021 **(C)**. EAPC, estimated annual percentage change; SDI, sociodemographic index.

SUPPLEMENTARY FIGURE S4 **(A)** Age-standardized prevalence rates of encephalitis for 21 regions by SDI from 1990–2021. The expected values based on the SDI and disease rates at all of the locations are shown as black lines. **(B)** Age-standardized prevalence rates for encephalitis in 204 countries and territories by SDI in 2021. Expected values based on the sociodemographic index and disease rate at all of the locations are shown as black lines. SDI, sociodemographic index.

SUPPLEMENTARY FIGURE S5 **(A)** Age-standardized incidence rates of encephalitis for 21 regions by SDI from 1990–2021. The expected values based on the SDI and disease rates at all of the locations are shown as black lines. **(B)** Age-standardized incidence rates for encephalitis in 204 countries and territories by SDI in 2021. Expected values based on the sociodemographic index and disease rate at all of the locations are shown as black lines. SDI, sociodemographic index.

SUPPLEMENTARY FIGURE S6 **(A)** Age-standardized mortality rates of encephalitis for 21 regions by SDI from 1990–2021. The expected values based on the SDI and disease rates at all of the locations are shown as black lines. **(B)** Age-standardized mortality rates for encephalitis in 204 countries and territories by SDI in 2021. Expected values based on the sociodemographic index and disease rate at all of the locations are shown as black lines. SDI, sociodemographic index.

SUPPLEMENTARY FIGURE S7 EAPCs in the age-standardized prevalence rates of encephalitis in 204 countries and territories from 1990 to 2021. EAPC, estimated annual percentage change.

SUPPLEMENTARY FIGURE S8 EAPCs in the age-standardized incidence rates of encephalitis in 204 countries and territories from 1990 to 2021. EAPC, estimated annual percentage change.

SUPPLEMENTARY FIGURE S9 EAPCs in the age-standardized DALYs rates for encephalitis in 204 countries and territories from 1990 to 2021. EAPC, estimated annual percentage change.

SUPPLEMENTARY FIGURE S10 EAPCs in the age-standardized mortality rates for encephalitis in 204 countries and territories from 1990 to 2021. EAPC, estimated annual percentage change.

SUPPLEMENTARY FIGURE S11 **(A)** Global cases and age-standardized incidence rates of encephalitis per 100,000 people by age and sex in 2021. **(B)** DALYs rates and age-standardized DALYs rates of encephalitis per 100,000 people by age and sex in 2021. **(C)** Global cases and age-standardized mortality rates of encephalitis per 100,000 people by age and sex in 2021. Shading indicates the upper and lower limits of the 95% uncertainty intervals.

SUPPLEMENTARY FIGURE S12 Future forecasts of the global burden of encephalitis. **(A)** Age-standardized prevalence rates. **(B)** Age-standardized incidence rates. **(C)** Age-standardized DALYs rates. **(D)** Age-standardized mortality rates.

SUPPLEMENTARY FIGURE S13 Future forecasts of age-specific prevalence cases of encephalitis.

SUPPLEMENTARY FIGURE S14 Future forecasts of the age-specific age-standardized prevalence rate of encephalitis.

SUPPLEMENTARY FIGURE S15 Future forecasts of the age-specific incidence cases of encephalitis.

SUPPLEMENTARY FIGURE S16 Future forecasts of the age-specific age-standardized incidence rate of encephalitis.

SUPPLEMENTARY FIGURE S17 Future forecasts of age-specific DALYs of encephalitis patients.

SUPPLEMENTARY FIGURE S18 Future forecasts of the age-specific age-standardized DALYs rate of encephalitis.

SUPPLEMENTARY FIGURE S19 Future forecasts of the age-specific cases of encephalitis mortality.

SUPPLEMENTARY FIGURE S20 Future forecasts of the age-specific age-standardized mortality rate of encephalitis patients.

SUPPLEMENTARY FIGURE S21 Age-standardized DALYs rates for encephalitis in Asia countries by SDI in 2021. Expected values based on the sociodemographic index and disease rate at all of the locations are shown as black lines. Note: SDI, sociodemographic index; DALYs, Disability-Adjusted Life Years.

SUPPLEMENTARY FIGURE S22 Age-standardized DALYs rates for encephalitis in Europe countries by SDI in 2021. Expected values based on the sociodemographic index and disease rate at all of the locations are shown as black lines. Note: SDI, sociodemographic index; DALYs, Disability-Adjusted Life Years.

SUPPLEMENTARY FIGURE S23 Age-standardized DALYs rates for encephalitis in Africa countries by SDI in 2021. Expected values based on the sociodemographic index and disease rate at all of the locations are shown as black lines. Note: SDI, sociodemographic index; DALYs, Disability-Adjusted Life Years.

SUPPLEMENTARY FIGURE S24 Age-standardized DALYs rates for encephalitis in Oceania countries by SDI in 2021. Expected values based on the sociodemographic index and disease rate at all of the locations are shown as black lines. Note: SDI, sociodemographic index; DALYs, Disability-Adjusted Life Years.

SUPPLEMENTARY FIGURE S25 Age-standardized DALYs rates for encephalitis in North America countries by SDI in 2021. Expected values based on the sociodemographic index and disease rate at all of the locations are shown as black lines. Note: SDI, sociodemographic index; DALYs, Disability-Adjusted Life Years.

SUPPLEMENTARY FIGURE S26 Age-standardized DALYs rates for encephalitis in South America countries by SDI in 2021. Expected values based on the sociodemographic index and disease rate at all of the locations are shown as black lines. Note: SDI, sociodemographic index; DALYs, Disability-Adjusted Life Years.
