# Supplementary material for: Socioeconomic drivers of encephalitis burden in the post-COVID era: a 204-country analysis from global burden of disease study 2021
Source: Front Public Health. 2025 Sep 18;13:1651734. doi: 10.3389/fpubh.2025.1651734 (PMC12488571; doi:10.3389/fpubh.2025.1651734)
Supplement: SUPPLEMENTARY FIGURE S1 — EAPCs in the age-standardized incidence rates for encephalitis in the SDI quintile (A) and in 21 regions (B) and the age-standardized incidence rates for encephalitis in 21 regions in 1990 and 2021 (C). EAPC, estimated annual percentage change; SDI, sociodemographic index. [file Data_Sheet_1.PDF]

Table S1: The ASDR and ASMR of encephalitis in 1990 and 2021, and changes from 1990 to 2021 at the global level and different regions.

	**ASDR per 100,000 (95% UI)**		**EAPC (95% CI)**	**ASDR per 100,000 (95% UI)**		**EAPC (95% CI)**
Characteristics	1990	2021		1990	2021	
Global	105.5 (83.4 - 120.0)	67.4 (54.9 - 78.4)	-0.96 (-1.06 to -0.86)	1.62 (1.29 - 1.81)	1.19 (1.00 - 1.37)	-1.18 (-1.34 to -1.02)
**sex**						
Male	99.1 (64.3 - 122.2)	67.7 (49.2 - 84.1)	-0.98 (-1.01 to -0.94)	1.64 (1.04 - 1.92)	1.21 (0.89 - 1.53)	-1.15 (-1.32 to -0.98)
Female	112.9 (87.6 - 133.2)	67.2 (58.2 - 78.0)	-1.41 (-1.45 to -1.36)	1.64 (1.33 - 1.88)	1.17 (1.01 - 1.35)	-1.19 (-1.35 to -1.04)
**SDI**						
High SDI	13.4 (12.6 - 14.6)	11.1 (10.4 - 11.9)	-0.57 (-0.77 to -0.37)	0.22 (0.21 - 0.24)	0.24 (0.22 - 0.25)	0.16 (-0.09 to 0.43)
High-middle SDI	67.4 (55.0 - 78.2)	29.3 (26.1 - 34.6)	-3.1 (-3.27 to -2.93)	0.90 (0.75 - 1.03)	0.47 (0.43 - 0.54)	-2.52 (-2.68 to -2.37)
Middle SDI	108.6 (84.1 - 123.4)	63.0 (51.5 - 72.5)	-1.94 (-2.10 to -1.78)	1.68 (1.32 - 1.88)	1.13 (0.95 - 1.32)	-1.42 (-1.57 to -1.26)
Low-middle SDI	173.8 (138.3 - 202.1)	110.5 (89.3 - 134.0)	-1.69 (-1.88 to -1.50)	3.57 (2.85 - 4.18)	2.65 (2.16 - 3.20)	-1.17 (-1.38 to -0.98)
Low SDI	156.4 (115.7 - 193.1)	94.8 (78.4 - 111.6)	-1.74 (-1.85 to -1.64)	2.71 (2.16 - 3.26)	1.88 (1.59 - 2.55)	-1.22 (-1.33 to -1.12)
**Regions**						
Andean Latin America	95.4 (77.2 - 115.3)	54.8 (41.6 - 71.3)	-2.11 (-2.50 to -1.73)	1.32 (1.10 - 1.58)	0.86 (0.66 - 1.11)	-1.31 (1.10 to 1.58)
Australasia	3.3 (3.1 - 3.6)	7.2 (6.6 - 7.9)	3.07 (2.50 to 3.64)	0.07 (0.06 - 0.08)	0.18 (0.16 - 0.19)	-0.29 (-0.36 to -0.22)
Caribbean	66.7 (59.2 - 76.9)	31.8 (25.4 - 40.0)	-2.12 (-2.75 to -1.48)	0.98 (0.89 - 1.10)	0.45 (0.36 - 0.55)	-0.96 (-1.11 to -0.81)
Central Asia	93.7 (81.3 - 108.8)	67.1 (56.0 - 80.1)	-1.49 (-1.91 to -1.06)	1.28 (1.11 - 1.48)	0.98 (0.82 - 1.15)	-1.16 (-1.42 to -0.91)
Central Europe	50.7 (47.7 - 53.8)	20.9 (18.8 - 23.2)	-3.46 (-3.85 to -3.07)	0.72 (0.68 - 0.76)	0.39 (0.36 - 0.43)	-1.86 (-1.98 to -1.75)
Central Latin America	53.5 (50.4 - 57.4)	41.6 (36.0 - 48.8)	-0.84 (-1.18 to -0.50)	0.73 (0.70 - 0.77)	0.39 (0.58 - 0.76)	-0.80 (-0.91 to -0.69)
Central Sub-Saharan Africa	33.7 (24.3 - 48.5)	28.7 (21.9 - 36.7)	-0.5 (-0.59 to -0.42)	0.58 (0.42 - 0.75)	0.52 (0.39 - 0.69)	-0.10 (-0.26 to 0.06)
East Asia	97.1 (68.0 - 119.3)	29.6 (24.1 - 38.9)	-4.39 (-4.71 to -4.06)	1.09 (0.76 - 1.33)	0.33 (0.26 - 0.45)	-1.65 (-1.74 to -1.55)
Eastern Europe	65.0 (62.2 - 67.9)	43.9 (40.6 - 46.8)	-1.67 (-1.85 to -1.50)	1.06 (1.02 - 1.09)	0.86 (0.79 - 0.92)	-0.96 (-1.15 to -0.78)
Eastern Sub-Saharan Africa	67.2 (48.1 - 80.7)	54.6 (40.5 - 67.4)	-0.68 (-0.74 to -0.61)	1.01 (0.74 - 1.20)	0.86 (0.65 - 1.06)	-0.48 (-0.52 to -0.44)
High-income Asia Pacific	13.0 (12.0 - 15.6)	10.4 (9.5 - 11.4)	-1.37 (-1.79 to -0.94)	0.23 (0.22 - 0.27)	0.20 (0.18 - 0.22)	-0.21 (-0.46 to 0.03)
High-income North America	5.8 (5.7 - 5.9)	8.0 (7.6 - 8.5)	1.51 (0.98 to 2.05)	0.12 (0.11 - 0.13)	0.19 (0.18 - 0.20)	-1.52 (-1.73 to -1.30)
North Africa and Middle East	44.9 (36.3 - 54.8)	29.7 (24.6 - 36.1)	-1.35 (-1.41 to -1.28)	0.75 (0.62 - 0.92)	0.53 (0.44 - 0.64)	-0.72 (-0.81 to -0.63)
Oceania	24.8 (18.1 - 34.2)	29.7 (18.9 - 46.2)	0.62 (0.49 to 0.74)	0.39 (0.26 - 0.78)	0.45 (0.26 - 0.92)	-0.24 (-0.31 to -0.17)
South Asia	266.2 (213.5 - 308.8)	158.1 (128.0 - 196.7)	-1.96 (-2.19 to -1.73)	5.65 (4.53 - 6.47)	5.65 (4.53 - 6.47)	-2.52 (-2.74 to -2.30)
Southeast Asia	93.6 (67.2 - 119.1)	80.4 (52.0 - 99.5)	-0.47 (-0.54 to -0.40)	1.40 (1.10 - 1.70)	1.31 (0.91 - 1.59)	-1.67 (-1.83 to -1.50)
Southern Latin America	22.0 (20.8 - 23.2)	20.6 (18.9 - 22.3)	-0.18 (-0.73 to 0.38)	0.40 (0.38 - 0.42)	0.41 (0.38 - 0.44)	0.16 (-0.13 to 0.45)
Southern Sub-Saharan Africa	25.3 (18.7 - 29.1)	23.7 (17.8 - 29.0)	-0.23 (-0.56 to 0.10)	0.46 (0.33 - 0.53)	0.43 (0.32 - 0.51)	-0.50 (-0.54 to -0.46)
Tropical Latin America	23.6 (21.8 - 25.7)	14.7 (13.1 - 16.3)	-1.48 (-1.64 to -1.32)	0.33 (0.30 - 0.35)	0.26 (0.24 - 0.29)	-2.00 (-2.38 to -1.62)
Western Europe	9.2 (8.8 - 9.6)	11.7 (11.2 - 12.3)	0.90 (0.76 to 1.05)	0.17 (0.16 - 0.18)	0.28 (0.26 - 0.29)	-0.02 (-0.16 to 0.13)
Western Sub-Saharan Africa	116.9 (82.4 - 157.9)	86.1 (61.2 - 107.8)	-0.87 (-1.07 to -0.67)	1.86 (1.41 - 2.34)	1.47 (1.04 - 1.82)	-0.45 (-0.51 to -0.38)

ASDR age-standardized DALYs rate, *ASMR* age-standardized mortality rate, *EAPC* estimated annual percentage change, *CI* confidence interval, *UI* uncertainty interval
