# Supplementary material for: Socioeconomic drivers of encephalitis burden in the post-COVID era: a 204-country analysis from global burden of disease study 2021
Source: Front Public Health. 2025 Sep 18;13:1651734. doi: 10.3389/fpubh.2025.1651734 (PMC12488571; doi:10.3389/fpubh.2025.1651734)
Supplement: SUPPLEMENTARY FIGURE S2 — EAPCs in the age-standardized mortality rates for encephalitis in the SDI quintile (A) and in 21 regions (B) and the age-standardized mortality rates for encephalitis in 21 regions in 1990 and 2021 (C). EAPC, estimated annual percentage change; SDI, sociodemographic index. [file Data_Sheet_2.PDF]

Table S2: The ASPR of encephalitis in 204 countries and territorie in 1990 and 2021 and EAPC of ASPR for encephalitis in 204 countries and territories from 1990 to 2021

age_name	measure_name	metric_name	location_name	sex_name	cause_name	1990 ASPR	1990 ASPR 95%UI upper	1990 ASPR 95%UI lower	2021 ASPR	2021 ASPR 95%UI upper	2021 ASPR 95%UI lower	EAPC (95%CI)	percentage change (100%)
Age-standardized	Prevalence	Rate	Afghanistan	Both	Encephalitis	64.1	86.4	40.3	69.4	91.2	45.4	-0.228(-0.454,-0.002)	8.32
Age-standardized	Prevalence	Rate	Albania	Both	Encephalitis	31.9	40.8	22.5	18.1	22.8	13.1	-2.181(-2.327,-2.036)	-43.09
Age-standardized	Prevalence	Rate	Algeria	Both	Encephalitis	19.3	25.4	13.3	18.1	23.4	12.9	-0.374(-0.430,-0.318)	-6.11
Age-standardized	Prevalence	Rate	American Samoa	Both	Encephalitis	26.7	35.3	18.3	29.2	38.3	20.1	0.223(0.175,0.271)	9.32
Age-standardized	Prevalence	Rate	Andorra	Both	Encephalitis	6.8	8.7	4.9	6.5	8.3	4.8	-0.193(-0.322,-0.065)	-4.61
Age-standardized	Prevalence	Rate	Angola	Both	Encephalitis	31.8	43.2	19.8	26.9	35.4	17.9	-0.940(-1.074,-0.806)	-15.21
Age-standardized	Prevalence	Rate	Antigua and Barbuda	Both	Encephalitis	34.3	44.3	24.3	26.9	34.6	19.1	-0.696(-0.773,-0.619)	-21.63
Age-standardized	Prevalence	Rate	Argentina	Both	Encephalitis	12.5	16.3	8.8	11.3	14.5	8	0.444(-0.118,1.009)	-10.07
Age-standardized	Prevalence	Rate	Armenia	Both	Encephalitis	37.9	49.7	26.5	30.3	38.7	21.6	-1.342(-1.581,-1.103)	-20.09
Age-standardized	Prevalence	Rate	Australia	Both	Encephalitis	0.3	0.3	0.2	0.3	0.4	0.2	0.403(0.357,0.449)	13.03
Age-standardized	Prevalence	Rate	Austria	Both	Encephalitis	22.1	27.9	15.8	17.8	22.5	13	-0.620(-0.696,-0.544)	-19.52
Age-standardized	Prevalence	Rate	Azerbaijan	Both	Encephalitis	41.6	54	28.2	34	43.2	24	-1.719(-2.109,-1.328)	-18.21
Age-standardized	Prevalence	Rate	Bahamas	Both	Encephalitis	30.7	40	21.3	25.7	33.2	18	-0.527(-0.712,-0.343)	-16.12
Age-standardized	Prevalence	Rate	Bahrain	Both	Encephalitis	15.3	20.1	10.8	13.5	17.2	9.8	-0.355(-0.421,-0.289)	-11.37
Age-standardized	Prevalence	Rate	Bangladesh	Both	Encephalitis	153.8	210	96.3	113.5	148.6	78.6	-0.965(-1.122,-0.808)	-26.17
Age-standardized	Prevalence	Rate	Barbados	Both	Encephalitis	46.1	58.9	32.3	36.6	47.1	25.6	-0.606(-0.861,-0.351)	-20.43
Age-standardized	Prevalence	Rate	Belarus	Both	Encephalitis	29.6	38.2	20.6	25.6	32.9	18.2	-0.895(-1.039,-0.750)	-13.58
Age-standardized	Prevalence	Rate	Belgium	Both	Encephalitis	4.9	6.4	3.6	4.8	6	3.5	0.050(-0.080,0.181)	-3.57
Age-standardized	Prevalence	Rate	Belize	Both	Encephalitis	50.5	65.9	34.8	34.9	45.5	24	-1.056(-1.236,-0.876)	-30.86
Age-standardized	Prevalence	Rate	Benin	Both	Encephalitis	50.2	68.1	31.8	45.2	59.3	30.3	-0.346(-0.393,-0.299)	-9.93
Age-standardized	Prevalence	Rate	Bermuda	Both	Encephalitis	32.8	41.9	23.1	22.8	29.2	16.7	-1.157(-1.307,-1.006)	-30.25
Age-standardized	Prevalence	Rate	Bhutan	Both	Encephalitis	187.7	249.3	122.1	132.4	170.3	92.9	-1.279(-1.382,-1.177)	-29.47
Age-standardized	Prevalence	Rate	Bolivia (Plurinational State of)	Both	Encephalitis	36.4	49	23.6	28.7	37.2	20.1	-0.860(-0.923,-0.797)	-21.25
Age-standardized	Prevalence	Rate	Bosnia and Herzegovina	Both	Encephalitis	29.4	37.8	20.8	15.3	19.4	11.1	-2.062(-2.232,-1.891)	-47.91
Age-standardized	Prevalence	Rate	Botswana	Both	Encephalitis	27.5	36.9	17.8	20.4	27	13.2	-0.792(-0.938,-0.646)	-25.80
Age-standardized	Prevalence	Rate	Brazil	Both	Encephalitis	20.2	26.2	13.7	14.4	18.4	10.1	-2.041(-2.430,-1.650)	-28.68
Age-standardized	Prevalence	Rate	Brunei Darussalam	Both	Encephalitis	8.7	11.5	6	10	13	7.1	0.494(0.446,0.543)	15.49
Age-standardized	Prevalence	Rate	Bulgaria	Both	Encephalitis	17.2	22.2	12	15.1	19.3	10.7	-0.645(-0.789,-0.501)	-12.29
Age-standardized	Prevalence	Rate	Burkina Faso	Both	Encephalitis	50.3	68.6	30.5	44.1	59.1	28.9	-0.475(-0.529,-0.421)	-12.22
Age-standardized	Prevalence	Rate	Burundi	Both	Encephalitis	42.2	58.9	25.6	50.6	67.8	33.2	0.707(0.645,0.770)	20.04
Age-standardized	Prevalence	Rate	Cabo Verde	Both	Encephalitis	55.8	73.4	37.2	41	52.7	28.5	-0.963(-1.060,-0.867)	-26.52
Age-standardized	Prevalence	Rate	Cambodia	Both	Encephalitis	54.1	73.8	34	36.2	47.1	24.6	-1.453(-1.546,-1.360)	-33.06
Age-standardized	Prevalence	Rate	Cameroon	Both	Encephalitis	42.2	56.9	27	40	53	26.5	-0.275(-0.333,-0.217)	-5.24
Age-standardized	Prevalence	Rate	Canada	Both	Encephalitis	0.2	0.3	0.1	0.1	0.2	0.1	-1.596(-2.120,-1.069)	-33.34
Age-standardized	Prevalence	Rate	Central African Republic	Both	Encephalitis	36.6	51.3	21.7	39.1	54.7	24.3	0.351(0.289,0.413)	6.82
Age-standardized	Prevalence	Rate	Chad	Both	Encephalitis	51.8	70.4	32.3	46.4	62.4	29.8	-0.560(-0.682,-0.437)	-10.39
Age-standardized	Prevalence	Rate	Chile	Both	Encephalitis	21.7	28.2	15.1	22.8	28.8	16.3	-0.121(-0.283,0.041)	5.10
Age-standardized	Prevalence	Rate	China	Both	Encephalitis	108.1	142.4	73.1	59.9	76.3	42.9	-1.678(-1.775,-1.581)	-44.57
Age-standardized	Prevalence	Rate	Colombia	Both	Encephalitis	51.8	68.1	35.5	44.2	56.6	31.6	-0.511(-0.674,-0.348)	-14.73
Age-standardized	Prevalence	Rate	Comoros	Both	Encephalitis	38.5	51.6	24.6	39.8	52.5	27.3	0.100(0.023,0.177)	3.20
Age-standardized	Prevalence	Rate	Congo	Both	Encephalitis	30.2	40.7	19.3	28.9	38.2	19.4	-0.303(-0.355,-0.252)	-4.46
Age-standardized	Prevalence	Rate	Cook Islands	Both	Encephalitis	27	35.4	18.7	21.8	28.6	15.5	-0.675(-0.763,-0.588)	-19.32
Age-standardized	Prevalence	Rate	Costa Rica	Both	Encephalitis	74.1	95.3	52.7	46.1	59	33.1	-1.460(-1.567,-1.353)	-37.77
Age-standardized	Prevalence	Rate	Croatia	Both	Encephalitis	21.1	27.1	14.9	18.8	23.6	13.7	-0.343(-0.473,-0.213)	-10.85
Age-standardized	Prevalence	Rate	Cuba	Both	Encephalitis	53.6	69.1	37.6	38.2	49.1	27.5	-1.388(-1.499,-1.277)	-28.72
Age-standardized	Prevalence	Rate	Cyprus	Both	Encephalitis	5.3	6.9	3.8	4.6	5.9	3.4	-0.191(-0.565,0.185)	-13.96
Age-standardized	Prevalence	Rate	Czechia	Both	Encephalitis	13.6	17.7	9.6	11.4	14.5	8.3	-0.647(-0.727,-0.566)	-16.71
Age-standardized	Prevalence	Rate	Cote d'Ivoire	Both	Encephalitis	39.8	54.8	24.6	38.9	50.9	25.9	0.021(-0.074,0.115)	-2.41
Age-standardized	Prevalence	Rate	Democratic People's Republic of Korea	Both	Encephalitis	67.1	88.7	45.7	67.6	87.5	47.3	0.242(0.148,0.336)	0.69
Age-standardized	Prevalence	Rate	Democratic Republic of the Congo	Both	Encephalitis	38.5	52.4	23.8	43.7	59.5	28.7	0.126(-0.083,0.335)	13.46
Age-standardized	Prevalence	Rate	Denmark	Both	Encephalitis	5.6	7.2	4	5.4	6.9	4	-0.162(-0.831,0.512)	-3.13
Age-standardized	Prevalence	Rate	Djibouti	Both	Encephalitis	36.7	49.5	23.9	34.6	46	23.1	-0.233(-0.342,-0.124)	-5.65
Age-standardized	Prevalence	Rate	Dominica	Both	Encephalitis	52.1	67.3	36.3	42.1	53.7	29.7	-0.634(-0.751,-0.518)	-19.21
Age-standardized	Prevalence	Rate	Dominican Republic	Both	Encephalitis	43.8	57.5	30	29.7	38.5	20.7	-1.204(-1.276,-1.133)	-32.15
Age-standardized	Prevalence	Rate	Ecuador	Both	Encephalitis	27.8	36.1	19.2	20.4	26.2	14.6	-1.416(-1.617,-1.216)	-26.58
Age-standardized	Prevalence	Rate	Egypt	Both	Encephalitis	21.5	28.6	14.4	17.4	22.6	12.2	-0.786(-0.868,-0.704)	-19.35
Age-standardized	Prevalence	Rate	El Salvador	Both	Encephalitis	67	88.7	45.5	47	61.4	32.7	-1.068(-1.142,-0.995)	-29.87
Age-standardized	Prevalence	Rate	Equatorial Guinea	Both	Encephalitis	38.3	51.8	24	18.7	24.6	12.4	-2.794(-3.512,-2.070)	-51.06
Age-standardized	Prevalence	Rate	Eritrea	Both	Encephalitis	44	61	26.3	41.1	54.9	27	0.089(-0.029,0.207)	-6.58
Age-standardized	Prevalence	Rate	Estonia	Both	Encephalitis	23.4	30.2	16.2	17.3	22.2	12.3	-1.047(-1.121,-0.973)	-25.92
Age-standardized	Prevalence	Rate	Eswatini	Both	Encephalitis	29.1	38.7	19.1	23.6	31.9	15.1	-0.724(-0.856,-0.593)	-18.84
Age-standardized	Prevalence	Rate	Ethiopia	Both	Encephalitis	55.6	76.2	33.1	42	55.1	28.3	-1.062(-1.220,-0.904)	-24.46
Age-standardized	Prevalence	Rate	Fiji	Both	Encephalitis	42.7	56.7	28.4	35.5	46.8	24.3	-0.686(-0.738,-0.633)	-16.98
Age-standardized	Prevalence	Rate	Finland	Both	Encephalitis	14.7	18.8	10.5	13.3	16.9	9.6	0.137(-0.078,0.351)	-9.29
Age-standardized	Prevalence	Rate	France	Both	Encephalitis	3.6	4.6	2.5	3.4	4.3	2.5	-0.938(-1.217,-0.659)	-5.72
Age-standardized	Prevalence	Rate	Gabon	Both	Encephalitis	21.9	29.2	14.4	21.5	28.2	14.4	-0.027(-0.090,0.037)	-1.73
Age-standardized	Prevalence	Rate	Gambia	Both	Encephalitis	46.9	63.2	29.7	48.2	63.9	31.6	0.085(0.047,0.123)	2.80
Age-standardized	Prevalence	Rate	Georgia	Both	Encephalitis	30.9	40.2	21.2	25.9	32.9	18.4	-1.722(-2.145,-1.297)	-16.15
Age-standardized	Prevalence	Rate	Germany	Both	Encephalitis	10.2	13.1	7.2	9.2	11.7	6.8	0.316(-0.151,0.785)	-9.11
Age-standardized	Prevalence	Rate	Ghana	Both	Encephalitis	50.3	66.5	32	43.3	56.1	29.1	-0.565(-0.608,-0.521)	-13.91
Age-standardized	Prevalence	Rate	Greece	Both	Encephalitis	21.4	27.5	15.3	22.4	28.3	16.3	0.765(0.463,1.069)	4.66
Age-standardized	Prevalence	Rate	Greenland	Both	Encephalitis	0.4	0.6	0.3	0.5	0.6	0.3	0.431(0.353,0.509)	15.06
Age-standardized	Prevalence	Rate	Grenada	Both	Encephalitis	51.4	67	35.3	32.2	41.6	22.6	-1.548(-1.739,-1.357)	-37.42
Age-standardized	Prevalence	Rate	Guam	Both	Encephalitis	20.5	26.8	14.2	18.2	24.3	12.7	-0.436(-0.474,-0.397)	-11.14
Age-standardized	Prevalence	Rate	Guatemala	Both	Encephalitis	50.8	68.6	32.8	43.5	57	30.2	-0.531(-0.576,-0.485)	-14.28
Age-standardized	Prevalence	Rate	Guinea	Both	Encephalitis	46.6	63.9	29.3	44.5	59	29.2	-0.091(-0.205,0.023)	-4.51
Age-standardized	Prevalence	Rate	Guinea-Bissau	Both	Encephalitis	46.9	63.6	29.1	45.6	60.5	29.3	-0.001(-0.076,0.074)	-2.75
Age-standardized	Prevalence	Rate	Guyana	Both	Encephalitis	65.3	86.1	42.8	36.2	46.8	24.7	-1.427(-1.620,-1.234)	-44.63
Age-standardized	Prevalence	Rate	Haiti	Both	Encephalitis	52.5	72.7	32.6	50.6	67.1	33.1	-0.331(-0.627,-0.033)	-3.56
Age-standardized	Prevalence	Rate	Honduras	Both	Encephalitis	62.6	84	41.6	55.5	72.4	38.8	-0.383(-0.463,-0.302)	-11.34
Age-standardized	Prevalence	Rate	Hungary	Both	Encephalitis	14.7	18.9	10.1	11.3	14.5	8.1	-0.862(-0.922,-0.802)	-22.99
Age-standardized	Prevalence	Rate	Iceland	Both	Encephalitis	1.1	1.4	0.8	1	1.3	0.8	0.117(-0.082,0.317)	-5.01
Age-standardized	Prevalence	Rate	India	Both	Encephalitis	289.7	385.6	187.5	143.7	186.3	99.3	-2.854(-3.128,-2.580)	-50.40
Age-standardized	Prevalence	Rate	Indonesia	Both	Encephalitis	35.5	48.9	23.1	19.2	25.6	13.3	-2.705(-3.141,-2.267)	-45.81
Age-standardized	Prevalence	Rate	Iran (Islamic Republic of)	Both	Encephalitis	21.1	27.6	14.5	18.2	23.2	13.1	-0.639(-0.764,-0.513)	-13.94
Age-standardized	Prevalence	Rate	Iraq	Both	Encephalitis	33	43.6	22.4	26.6	34.1	18.8	-1.406(-1.612,-1.199)	-19.32
Age-standardized	Prevalence	Rate	Ireland	Both	Encephalitis	7.8	10	5.6	5.5	7	4	-1.006(-1.146,-0.867)	-29.81
Age-standardized	Prevalence	Rate	Israel	Both	Encephalitis	8	10.3	5.8	7	8.9	5.1	-0.383(-0.426,-0.340)	-12.54
Age-standardized	Prevalence	Rate	Italy	Both	Encephalitis	16.3	20.7	11.9	14.5	18	10.7	-0.470(-1.063,0.126)	-11.20
Age-standardized	Prevalence	Rate	Jamaica	Both	Encephalitis	55	70.7	38.8	41.2	52.9	29.3	-0.785(-0.949,-0.622)	-25.04
Age-standardized	Prevalence	Rate	Japan	Both	Encephalitis	21.4	27.5	15.3	18	23.1	13	0.062(-0.267,0.393)	-15.60
Age-standardized	Prevalence	Rate	Jordan	Both	Encephalitis	25.9	33.9	18.1	20.6	26.4	15	-0.940(-1.025,-0.854)	-20.81
Age-standardized	Prevalence	Rate	Kazakhstan	Both	Encephalitis	40.2	51.8	27.9	26.9	34.2	18.8	-2.186(-2.517,-1.853)	-32.90
Age-standardized	Prevalence	Rate	Kenya	Both	Encephalitis	42.7	56.4	27.7	39.8	52.2	26.6	0.238(0.062,0.415)	-6.61
Age-standardized	Prevalence	Rate	Kiribati	Both	Encephalitis	45.8	62.9	29.2	47.3	62.9	30.9	0.159(0.105,0.214)	3.26
Age-standardized	Prevalence	Rate	Kuwait	Both	Encephalitis	14.7	19.3	10.3	12.8	16.3	9.3	-0.188(-0.347,-0.029)	-12.96
Age-standardized	Prevalence	Rate	Kyrgyzstan	Both	Encephalitis	39.7	51.4	27	39.2	50.2	27.6	-0.679(-1.031,-0.326)	-1.30
Age-standardized	Prevalence	Rate	Lao People's Democratic Republic	Both	Encephalitis	45.9	63.5	28.3	30.6	40.4	20.8	-1.425(-1.562,-1.288)	-33.30
Age-standardized	Prevalence	Rate	Latvia	Both	Encephalitis	27.6	35.5	19.1	19	24.3	13.4	-1.690(-1.862,-1.517)	-31.22
Age-standardized	Prevalence	Rate	Lebanon	Both	Encephalitis	23.7	31	16.5	19.7	25.2	14.1	-0.441(-0.586,-0.296)	-16.92
Age-standardized	Prevalence	Rate	Lesotho	Both	Encephalitis	41.9	56.5	27.7	29.5	41.1	18.3	-1.123(-1.226,-1.021)	-29.55
Age-standardized	Prevalence	Rate	Liberia	Both	Encephalitis	54.2	73.7	34.1	55.2	73.5	36.4	-0.450(-0.655,-0.244)	1.73
Age-standardized	Prevalence	Rate	Libya	Both	Encephalitis	11.3	14.7	7.9	15.1	19.5	10.6	0.987(0.694,1.281)	33.34
Age-standardized	Prevalence	Rate	Lithuania	Both	Encephalitis	24.2	31.2	16.6	17.2	22	12.1	-1.461(-1.601,-1.321)	-28.88
Age-standardized	Prevalence	Rate	Luxembourg	Both	Encephalitis	8.6	11.2	6.1	7.4	9.3	5.4	-0.719(-0.834,-0.603)	-14.61
Age-standardized	Prevalence	Rate	Madagascar	Both	Encephalitis	42.3	56.9	26.7	41.7	55.7	27.5	-0.183(-0.263,-0.103)	-1.40
Age-standardized	Prevalence	Rate	Malawi	Both	Encephalitis	46.6	63.4	29	44.7	59.1	29.2	0.078(-0.004,0.159)	-4.05
Age-standardized	Prevalence	Rate	Malaysia	Both	Encephalitis	43	55.8	29.9	30.2	38.9	21	-1.020(-1.060,-0.981)	-29.63
Age-standardized	Prevalence	Rate	Maldives	Both	Encephalitis	40.2	52.8	26.9	27.5	35.3	19.8	-1.288(-1.345,-1.231)	-31.48
Age-standardized	Prevalence	Rate	Mali	Both	Encephalitis	57.8	78.8	36	51.5	68.1	34.1	-0.429(-0.509,-0.349)	-10.82
Age-standardized	Prevalence	Rate	Malta	Both	Encephalitis	4	5.1	2.9	3.3	4.3	2.4	-0.201(-0.501,0.099)	-17.52
Age-standardized	Prevalence	Rate	Marshall Islands	Both	Encephalitis	40.9	55.1	26.8	37.1	49.8	24.8	-0.215(-0.282,-0.148)	-9.35
Age-standardized	Prevalence	Rate	Mauritania	Both	Encephalitis	48.3	65	31.5	43.6	56.9	29.9	-0.383(-0.436,-0.329)	-9.78
Age-standardized	Prevalence	Rate	Mauritius	Both	Encephalitis	32.4	42	22.6	28.5	36.6	20.1	-0.210(-0.298,-0.123)	-12.03
Age-standardized	Prevalence	Rate	Mexico	Both	Encephalitis	46.6	60.8	32.3	42.7	54.8	30.4	-0.965(-1.180,-0.750)	-8.43
Age-standardized	Prevalence	Rate	Micronesia (Federated States of)	Both	Encephalitis	40.7	55.5	26.6	38	50.4	25.1	-0.130(-0.161,-0.100)	-6.69
Age-standardized	Prevalence	Rate	Monaco	Both	Encephalitis	3.6	4.7	2.6	3.1	4	2.3	-0.513(-0.575,-0.452)	-13.84
Age-standardized	Prevalence	Rate	Mongolia	Both	Encephalitis	42	56.1	27.7	30.4	39	21	-1.361(-1.527,-1.194)	-27.70
Age-standardized	Prevalence	Rate	Montenegro	Both	Encephalitis	15.9	20.5	11.2	13.2	17	9.3	-1.040(-1.194,-0.886)	-17.31
Age-standardized	Prevalence	Rate	Morocco	Both	Encephalitis	23.3	31.1	15.5	20.2	26.4	14.3	-0.496(-0.589,-0.403)	-12.97
Age-standardized	Prevalence	Rate	Mozambique	Both	Encephalitis	57.2	78.1	35.3	43.5	58.5	27.2	-1.132(-1.239,-1.024)	-24.05
Age-standardized	Prevalence	Rate	Myanmar	Both	Encephalitis	48.3	67.8	29.9	27.8	36.7	18.8	-1.991(-2.112,-1.870)	-42.46
Age-standardized	Prevalence	Rate	Namibia	Both	Encephalitis	30.8	40.7	20.2	24.8	32.8	16.5	-0.822(-0.913,-0.730)	-19.54
Age-standardized	Prevalence	Rate	Nauru	Both	Encephalitis	23.8	32.1	15.7	25.5	34.1	16.8	-0.227(-0.636,0.184)	7.04
Age-standardized	Prevalence	Rate	Nepal	Both	Encephalitis	208	282.4	134.1	138.2	177.6	97	-1.596(-1.806,-1.386)	-33.57
Age-standardized	Prevalence	Rate	Netherlands	Both	Encephalitis	5.2	6.7	3.8	4.4	5.6	3.3	-0.543(-0.593,-0.493)	-15.65
Age-standardized	Prevalence	Rate	New Zealand	Both	Encephalitis	11.2	14.4	8	10.2	12.8	7.3	-0.348(-0.403,-0.293)	-9.10
Age-standardized	Prevalence	Rate	Nicaragua	Both	Encephalitis	73.3	96.3	50.4	63.3	81.6	44.5	-0.598(-0.638,-0.558)	-13.66
Age-standardized	Prevalence	Rate	Niger	Both	Encephalitis	54.9	75.5	34.3	55.1	73.6	35.8	-0.072(-0.148,0.004)	0.34
Age-standardized	Prevalence	Rate	Nigeria	Both	Encephalitis	43.6	57.9	28.3	39.5	51.7	26.6	-0.664(-0.802,-0.526)	-9.36
Age-standardized	Prevalence	Rate	Niue	Both	Encephalitis	33.9	45.5	23	28.8	37.8	19.9	-0.615(-0.693,-0.537)	-15.11
Age-standardized	Prevalence	Rate	North Macedonia	Both	Encephalitis	17.1	22.2	12.1	13.8	17.7	9.9	-0.833(-0.915,-0.751)	-19.25
Age-standardized	Prevalence	Rate	Northern Mariana Islands	Both	Encephalitis	21.5	28.4	14.8	22.9	30.1	16.1	0.486(0.253,0.720)	6.89
Age-standardized	Prevalence	Rate	Norway	Both	Encephalitis	7.3	9.4	5.3	5.8	7.3	4.3	-0.599(-0.751,-0.446)	-21.48
Age-standardized	Prevalence	Rate	Oman	Both	Encephalitis	38.1	49.2	26.2	21.4	27.1	15.5	-2.354(-2.549,-2.158)	-43.87
Age-standardized	Prevalence	Rate	Pakistan	Both	Encephalitis	171.6	224.7	113.1	145.2	190.5	97.9	-0.559(-0.629,-0.488)	-15.37
Age-standardized	Prevalence	Rate	Palau	Both	Encephalitis	28.3	38	19	25.6	33.5	17.5	-0.414(-0.489,-0.339)	-9.83
Age-standardized	Prevalence	Rate	Palestine	Both	Encephalitis	29	37.9	19.9	25.7	33.3	18.1	-0.274(-0.340,-0.208)	-11.28
Age-standardized	Prevalence	Rate	Panama	Both	Encephalitis	61.2	79.5	42.7	35.9	46.4	25.8	-1.698(-1.732,-1.663)	-41.28
Age-standardized	Prevalence	Rate	Papua New Guinea	Both	Encephalitis	43.9	58.7	28.2	39.7	52.5	26.4	-0.246(-0.333,-0.160)	-9.61
Age-standardized	Prevalence	Rate	Paraguay	Both	Encephalitis	23.3	30.1	16.3	18.3	23.7	13	-0.835(-0.923,-0.748)	-21.48
Age-standardized	Prevalence	Rate	Peru	Both	Encephalitis	39.8	52.7	26.7	27.9	35.7	19.8	-1.385(-1.574,-1.194)	-30.01
Age-standardized	Prevalence	Rate	Philippines	Both	Encephalitis	69.6	91.8	46.5	54.3	70.6	37.5	-0.807(-0.994,-0.619)	-21.94
Age-standardized	Prevalence	Rate	Poland	Both	Encephalitis	12.3	16.1	8.6	3.1	3.9	2.2	-4.748(-5.221,-4.272)	-74.97
Age-standardized	Prevalence	Rate	Portugal	Both	Encephalitis	1.8	2.3	1.3	1.4	1.8	1.1	-0.600(-0.967,-0.231)	-21.65
Age-standardized	Prevalence	Rate	Puerto Rico	Both	Encephalitis	33.2	42.7	23.4	24.6	31.2	17.6	-0.757(-0.936,-0.578)	-25.97
Age-standardized	Prevalence	Rate	Qatar	Both	Encephalitis	13.5	17.5	9.4	9.8	12.6	7.1	-1.209(-1.398,-1.020)	-27.04
Age-standardized	Prevalence	Rate	Republic of Korea	Both	Encephalitis	13.6	17.8	9.4	10.8	14	7.9	-0.637(-0.680,-0.594)	-20.42
Age-standardized	Prevalence	Rate	Republic of Moldova	Both	Encephalitis	28.9	37.6	19.9	27.4	35.2	19.1	-0.657(-0.974,-0.339)	-5.34
Age-standardized	Prevalence	Rate	Romania	Both	Encephalitis	25.5	32.8	17.8	15	19.2	10.5	-1.931(-2.082,-1.781)	-41.27
Age-standardized	Prevalence	Rate	Russian Federation	Both	Encephalitis	25.2	32.6	17.4	18.9	24.2	13.1	-1.430(-1.624,-1.236)	-25.19
Age-standardized	Prevalence	Rate	Rwanda	Both	Encephalitis	44.1	61.3	27.6	39.2	51.3	26.6	-0.499(-0.611,-0.387)	-11.07
Age-standardized	Prevalence	Rate	Saint Kitts and Nevis	Both	Encephalitis	50.9	65.3	34.9	28.9	37.5	20.5	-1.746(-2.015,-1.476)	-43.14
Age-standardized	Prevalence	Rate	Saint Lucia	Both	Encephalitis	53.7	68.5	37.4	36.6	46.8	25.6	-1.187(-1.355,-1.019)	-31.92
Age-standardized	Prevalence	Rate	Saint Vincent and the Grenadines	Both	Encephalitis	48.6	63	33.7	32.3	41.8	22.5	-1.216(-1.341,-1.091)	-33.55
Age-standardized	Prevalence	Rate	Samoa	Both	Encephalitis	40.2	53.2	26.8	34.6	45.8	23.7	-0.523(-0.579,-0.466)	-13.86
Age-standardized	Prevalence	Rate	San Marino	Both	Encephalitis	7.6	9.7	5.6	8.3	10.5	6	0.455(0.218,0.693)	8.79
Age-standardized	Prevalence	Rate	Sao Tome and Principe	Both	Encephalitis	66.5	89	43.1	65.3	83	45.4	-0.142(-0.240,-0.044)	-1.84
Age-standardized	Prevalence	Rate	Saudi Arabia	Both	Encephalitis	17.4	22.5	12.1	14.5	18.8	10.5	-0.857(-0.952,-0.762)	-16.80
Age-standardized	Prevalence	Rate	Senegal	Both	Encephalitis	48.1	64.1	31.4	46.2	60.3	31.3	-0.118(-0.169,-0.067)	-4.04
Age-standardized	Prevalence	Rate	Serbia	Both	Encephalitis	18.9	24.4	13.2	16.1	20.5	11.5	-1.070(-1.260,-0.880)	-14.66
Age-standardized	Prevalence	Rate	Seychelles	Both	Encephalitis	27.4	36.1	18.8	23	29.9	16	-0.575(-0.684,-0.466)	-16.11
Age-standardized	Prevalence	Rate	Sierra Leone	Both	Encephalitis	48.2	64.8	30.4	49	65.3	32.2	0.011(-0.120,0.142)	1.71
Age-standardized	Prevalence	Rate	Singapore	Both	Encephalitis	6.2	7.9	4.4	4.7	6.1	3.5	-1.133(-1.718,-0.545)	-23.50
Age-standardized	Prevalence	Rate	Slovakia	Both	Encephalitis	19.2	24.9	13.4	15	19.6	10.6	-0.881(-1.248,-0.513)	-21.85
Age-standardized	Prevalence	Rate	Slovenia	Both	Encephalitis	15.5	19.6	11	13.4	17.1	9.8	-0.483(-0.530,-0.435)	-13.54
Age-standardized	Prevalence	Rate	Solomon Islands	Both	Encephalitis	40.6	56.1	25.3	38.5	52.3	24.7	-0.057(-0.186,0.073)	-5.13
Age-standardized	Prevalence	Rate	Somalia	Both	Encephalitis	58.9	82	35.3	66.1	90.6	41.7	0.172(0.085,0.259)	12.34
Age-standardized	Prevalence	Rate	South Africa	Both	Encephalitis	27.4	36.2	18.1	21.6	28.7	13.8	-0.933(-1.001,-0.866)	-21.31
Age-standardized	Prevalence	Rate	South Sudan	Both	Encephalitis	29.6	39.8	18.3	36	48.7	22.8	0.555(0.342,0.769)	21.63
Age-standardized	Prevalence	Rate	Spain	Both	Encephalitis	7.2	9.1	5.2	8.9	11.3	6.6	1.234(0.740,1.730)	24.42
Age-standardized	Prevalence	Rate	Sri Lanka	Both	Encephalitis	59.3	77.5	40.3	37.6	48	26.8	-1.531(-1.581,-1.480)	-36.51
Age-standardized	Prevalence	Rate	Sudan	Both	Encephalitis	27	36.8	17.4	24	31.4	16.8	-0.600(-0.679,-0.522)	-11.17
Age-standardized	Prevalence	Rate	Suriname	Both	Encephalitis	51.9	66.9	35.7	37	48	25.9	-1.274(-1.462,-1.087)	-28.61
Age-standardized	Prevalence	Rate	Sweden	Both	Encephalitis	17.4	22.3	12.5	17.2	22.1	12.3	0.178(-0.008,0.365)	-0.98
Age-standardized	Prevalence	Rate	Switzerland	Both	Encephalitis	9.6	12.3	7	9.3	11.9	6.9	-0.120(-0.356,0.116)	-2.46
Age-standardized	Prevalence	Rate	Syrian Arab Republic	Both	Encephalitis	29.9	39.2	20.3	27	34.5	19.1	-0.610(-0.773,-0.446)	-9.70
Age-standardized	Prevalence	Rate	Taiwan (Province of China)	Both	Encephalitis	24.5	31.9	17	14.3	18.5	10.4	-1.734(-1.993,-1.474)	-41.53
Age-standardized	Prevalence	Rate	Tajikistan	Both	Encephalitis	36	47.3	24.5	37.1	48.5	25.9	-0.530(-0.906,-0.153)	3.23
Age-standardized	Prevalence	Rate	Thailand	Both	Encephalitis	52.2	67.6	36.2	30.7	38.9	21.9	-1.756(-1.870,-1.641)	-41.23
Age-standardized	Prevalence	Rate	Timor-Leste	Both	Encephalitis	49.2	66.4	32.1	37.8	48.8	25.9	-1.129(-1.373,-0.884)	-23.06
Age-standardized	Prevalence	Rate	Togo	Both	Encephalitis	50.1	68.1	32.2	48.4	64	31.9	-0.160(-0.217,-0.103)	-3.49
Age-standardized	Prevalence	Rate	Tokelau	Both	Encephalitis	40.5	53	27.2	32.8	43.2	22.5	-0.778(-0.826,-0.730)	-19.10
Age-standardized	Prevalence	Rate	Tonga	Both	Encephalitis	44.6	59.3	30.5	39.7	51.9	27.3	-0.299(-0.336,-0.261)	-11.11
Age-standardized	Prevalence	Rate	Trinidad and Tobago	Both	Encephalitis	47.3	61.4	32.8	23.9	30.9	16.7	-2.682(-2.976,-2.387)	-49.57
Age-standardized	Prevalence	Rate	Tunisia	Both	Encephalitis	23.9	31.4	16.5	19.8	25.3	14.2	-0.578(-0.607,-0.548)	-17.07
Age-standardized	Prevalence	Rate	Turkey	Both	Encephalitis	24.9	32.8	17.2	19.2	24.6	13.8	-1.223(-1.379,-1.066)	-23.17
Age-standardized	Prevalence	Rate	Turkmenistan	Both	Encephalitis	31.4	40.9	21.4	28.6	36.6	19.9	-0.852(-1.142,-0.562)	-8.75
Age-standardized	Prevalence	Rate	Tuvalu	Both	Encephalitis	40	54.1	25.7	36.3	48.5	24.7	-0.182(-0.296,-0.068)	-9.13
Age-standardized	Prevalence	Rate	Uganda	Both	Encephalitis	42.6	59.2	26.1	38.5	50.7	25.7	-0.233(-0.276,-0.190)	-9.68
Age-standardized	Prevalence	Rate	Ukraine	Both	Encephalitis	31	40.1	21.3	36.1	46.4	25.1	0.090(-0.100,0.282)	16.36
Age-standardized	Prevalence	Rate	United Arab Emirates	Both	Encephalitis	10.1	13.2	7	12.1	15.5	8.7	0.713(0.552,0.874)	19.31
Age-standardized	Prevalence	Rate	United Kingdom	Both	Encephalitis	11.5	15.2	8.2	13.2	16.9	9.6	0.041(-0.236,0.319)	14.25
Age-standardized	Prevalence	Rate	United Republic of Tanzania	Both	Encephalitis	43.7	58.7	27.5	38	50	25.5	-0.541(-0.578,-0.504)	-13.12
Age-standardized	Prevalence	Rate	United States of America	Both	Encephalitis	3.1	4.1	2.2	2.2	2.8	1.6	-1.498(-1.714,-1.282)	-27.72
Age-standardized	Prevalence	Rate	United States Virgin Islands	Both	Encephalitis	23.8	31.3	16.6	21.3	27.6	15.2	-0.466(-0.648,-0.284)	-10.69
Age-standardized	Prevalence	Rate	Uruguay	Both	Encephalitis	19.1	24.7	13.5	16.2	20.8	11.6	-0.703(-0.887,-0.519)	-15.55
Age-standardized	Prevalence	Rate	Uzbekistan	Both	Encephalitis	57.9	74.8	40	45.7	58.6	32.1	-1.001(-1.167,-0.835)	-20.99
Age-standardized	Prevalence	Rate	Vanuatu	Both	Encephalitis	40.8	55	26.5	39.4	53	25.8	-0.091(-0.124,-0.058)	-3.43
Age-standardized	Prevalence	Rate	Venezuela (Bolivarian Republic of)	Both	Encephalitis	53.6	69.9	37.4	46.8	60.7	32.8	-0.631(-0.835,-0.427)	-12.73
Age-standardized	Prevalence	Rate	Viet Nam	Both	Encephalitis	94.7	123.9	63.6	57.8	74	40.5	-1.728(-1.814,-1.641)	-38.92
Age-standardized	Prevalence	Rate	Yemen	Both	Encephalitis	23.6	32	14.9	27.2	35.7	18.4	0.373(0.199,0.547)	15.28
Age-standardized	Prevalence	Rate	Zambia	Both	Encephalitis	38	51.8	23.8	33.2	43.6	21.6	-0.676(-0.745,-0.608)	-12.84
Age-standardized	Prevalence	Rate	Zimbabwe	Both	Encephalitis	36.5	49.2	23.8	46.2	61.8	30.2	1.112(0.917,1.308)	26.67

ASPR age-standardized prevalence rate, *EAPC* estimated annual percentage change, *CI* confidence interval, *UI* uncertainty interval
