# Supplementary material for: Socioeconomic drivers of encephalitis burden in the post-COVID era: a 204-country analysis from global burden of disease study 2021
Source: Front Public Health. 2025 Sep 18;13:1651734. doi: 10.3389/fpubh.2025.1651734 (PMC12488571; doi:10.3389/fpubh.2025.1651734)
Supplement: SUPPLEMENTARY FIGURE S3 — EAPCs in the age-standardized DALYs rates for encephalitis in the SDI quintile (A) and in 21 regions (B) and the age-standardized DALYs rates for encephalitis in 21 regions in 1990 and 2021 (C). EAPC, estimated annual percentage change; SDI, sociodemographic index. [file Data_Sheet_3.PDF]

**Table S3: The ASIR of encephalitis in 204 countries and territories in 1990 and 2021 and EAPC of ASIR for encephalitis in 204 countries and territories from 1990 to 2021**

| age_name | measure_name | metric_nam | location_name | sex_name | cause_name | 1990 ASIR | 1990 ASIR 95%UI upper | 1990 ASIR 95%UI lower | 2021 ASIR | 2021 ASIR 95%UI upper | 2021 ASIR 95%UI lower | EAPC (95%CI) | percentage change (100%) |
|---|---|---|---|---|---|---|---|---|---|---|---|---|---|
| Age-standardized | Incidence | Rate | Afghanistan | Both | Encephalitis | 21.3 | 23.2 | 19.8 | 22 | 23.6 | 20.6 | 0.074(-0.011,0.160) | 3.31 |
| Age-standardized | Incidence | Rate | Albania | Both | Encephalitis | 10.4 | 11.4 | 9.3 | 7.5 | 8.3 | 6.7 | -1.357(-1.511,-1.203) | -27.91 |
| Age-standardized | Incidence | Rate | Algeria | Both | Encephalitis | 7.1 | 8.1 | 6.2 | 6.6 | 7.6 | 5.7 | -0.214(-0.242,-0.185) | -6.67 |
| Age-standardized | Incidence | Rate | American Samoa | Both | Encephalitis | 11 | 12.9 | 9.5 | 10.7 | 12.6 | 9.3 | -0.084(-0.144,-0.024) | -2.33 |
| Age-standardized | Incidence | Rate | Andorra | Both | Encephalitis | 4.1 | 4.7 | 3.7 | 4.1 | 4.7 | 3.7 | 0.042(-0.026,0.110) | 1.04 |
| Age-standardized | Incidence | Rate | Angola | Both | Encephalitis | 10.2 | 11.7 | 9 | 9.3 | 10.5 | 8.1 | -0.315(-0.343,-0.287) | -9.19 |
| Age-standardized | Incidence | Rate | Antigua and Barbuda | Both | Encephalitis | 12.4 | 14.3 | 10.9 | 10.9 | 12.5 | 9.4 | -0.417(-0.453,-0.381) | -12.61 |
| Age-standardized | Incidence | Rate | Argentina | Both | Encephalitis | 4.3 | 4.9 | 3.7 | 4.5 | 5.1 | 3.9 | 0.794(0.245,1.345) | 4.27 |
| Age-standardized | Incidence | Rate | Armenia | Both | Encephalitis | 11 | 12.7 | 9.4 | 10.5 | 11.9 | 9.1 | -0.153(-0.205,-0.101) | -4.86 |
| Age-standardized | Incidence | Rate | Australia | Both | Encephalitis | 0.3 | 0.3 | 0.2 | 0.6 | 0.7 | 0.5 | 2.863(2.424,3.303) | 114.42 |
| Age-standardized | Incidence | Rate | Austria | Both | Encephalitis | 10.8 | 11.9 | 9.9 | 9.6 | 10.7 | 8.6 | -0.364(-0.445,-0.283) | -11.8 |
| Age-standardized | Incidence | Rate | Azerbaijan | Both | Encephalitis | 15.1 | 16.9 | 13.5 | 14.5 | 16 | 13.1 | -0.194(-0.255,-0.132) | -4.26 |
| Age-standardized | Incidence | Rate | Bahamas | Both | Encephalitis | 14.3 | 16.2 | 12.7 | 11.6 | 13.4 | 10.1 | -0.627(-0.747,-0.508) | -19.18 |
| Age-standardized | Incidence | Rate | Bahrain | Both | Encephalitis | 6.8 | 7.9 | 5.9 | 6.4 | 7.3 | 5.5 | -0.226(-0.304,-0.148) | -6.64 |
| Age-standardized | Incidence | Rate | Bangladesh | Both | Encephalitis | 38.7 | 44 | 33.9 | 33 | 37.5 | 29 | -0.476(-0.531,-0.421) | -14.91 |
| Age-standardized | Incidence | Rate | Barbados | Both | Encephalitis | 17.6 | 19.8 | 15.7 | 13.3 | 15.1 | 11.9 | -0.912(-1.098,-0.725) | -24.33 |
| Age-standardized | Incidence | Rate | Belarus | Both | Encephalitis | 11.7 | 13.1 | 10.5 | 11.6 | 13 | 10.5 | 0.050(0.011,0.088) | -0.45 |
| Age-standardized | Incidence | Rate | Belgium | Both | Encephalitis | 2.3 | 2.7 | 2 | 2.7 | 3 | 2.3 | 0.607(0.391,0.824) | 13.4 |
| Age-standardized | Incidence | Rate | Belize | Both | Encephalitis | 14.9 | 16.8 | 13.4 | 11.1 | 12.7 | 9.7 | -0.898(-1.018,-0.777) | -25.74 |
| Age-standardized | Incidence | Rate | Benin | Both | Encephalitis | 14.7 | 16.5 | 13.1 | 13.5 | 15.1 | 12.1 | -0.283(-0.319,-0.246) | -8.8 |
| Age-standardized | Incidence | Rate | Bermuda | Both | Encephalitis | 14.9 | 16.8 | 13.3 | 11.4 | 13.3 | 9.9 | -0.800(-0.912,-0.688) | -23.19 |
| Age-standardized | Incidence | Rate | Bhutan | Both | Encephalitis | 63.6 | 69.7 | 58.6 | 52.4 | 56.8 | 48.5 | -0.768(-0.818,-0.719) | -17.55 |
| Age-standardized | Incidence | Rate | Bolivia (Plurinational State of) | Both | Encephalitis | 10.9 | 12.1 | 9.8 | 9.7 | 10.7 | 8.7 | -0.445(-0.460,-0.430) | -11.56 |
| Age-standardized | Incidence | Rate | Bosnia and Herzegovina | Both | Encephalitis | 7 | 7.9 | 6.2 | 5.9 | 6.7 | 5.1 | -0.591(-0.615,-0.567) | -15.5 |
| Age-standardized | Incidence | Rate | Botswana | Both | Encephalitis | 10.3 | 11.8 | 8.9 | 9.8 | 11.2 | 8.5 | -0.152(-0.189,-0.114) | -4.28 |
| Age-standardized | Incidence | Rate | Brazil | Both | Encephalitis | 7 | 7.8 | 6.3 | 5.6 | 6.3 | 5 | -1.570(-1.867,-1.272) | -20.55 |
| Age-standardized | Incidence | Rate | Brunei Darussalam | Both | Encephalitis | 5.4 | 6.3 | 4.7 | 5.9 | 6.8 | 5.2 | 0.351(0.334,0.369) | 9.38 |
| Age-standardized | Incidence | Rate | Bulgaria | Both | Encephalitis | 6.9 | 7.8 | 6.1 | 7.2 | 8.1 | 6.4 | 0.113(0.093,0.132) | 4.32 |
| Age-standardized | Incidence | Rate | Burkina Faso | Both | Encephalitis | 15.2 | 17 | 13.5 | 12.5 | 14.2 | 11.2 | -0.634(-0.666,-0.602) | -17.35 |
| Age-standardized | Incidence | Rate | Burundi | Both | Encephalitis | 12.2 | 13.6 | 11 | 11 | 12.3 | 9.9 | -0.350(-0.390,-0.309) | -9.58 |
| Age-standardized | Incidence | Rate | Cabo Verde | Both | Encephalitis | 14.8 | 16.6 | 13.2 | 14.6 | 16.2 | 13.1 | -0.048(-0.066,-0.031) | -1.76 |
| Age-standardized | Incidence | Rate | Cambodia | Both | Encephalitis | 13.2 | 14.8 | 11.8 | 11 | 12.4 | 9.8 | -0.686(-0.726,-0.646) | -16.54 |
| Age-standardized | Incidence | Rate | Cameroon | Both | Encephalitis | 14 | 15.8 | 12.5 | 13.3 | 14.9 | 12 | -0.134(-0.155,-0.112) | -4.77 |
| Age-standardized | Incidence | Rate | Canada | Both | Encephalitis | 0.4 | 0.4 | 0.3 | 0.7 | 0.9 | 0.6 | 2.238(1.819,2.660) | 98.06 |
| Age-standardized | Incidence | Rate | Central African Republic | Both | Encephalitis | 9.5 | 10.8 | 8.4 | 9 | 10.1 | 7.8 | -0.208(-0.249,-0.166) | -6.21 |
| Age-standardized | Incidence | Rate | Chad | Both | Encephalitis | 14.5 | 16.4 | 13 | 14.1 | 15.7 | 12.7 | -0.034(-0.055,-0.013) | -2.86 |
| Age-standardized | Incidence | Rate | Chile | Both | Encephalitis | 6.7 | 7.9 | 5.7 | 9 | 10.1 | 7.9 | 0.719(0.602,0.836) | 32.7 |
| Age-standardized | Incidence | Rate | China | Both | Encephalitis | 21.2 | 24.6 | 18.4 | 19.2 | 22.3 | 16.8 | -0.107(-0.219,0.005) | -9.36 |
| Age-standardized | Incidence | Rate | Colombia | Both | Encephalitis | 15.1 | 17.6 | 13 | 14.6 | 16.9 | 12.8 | -0.097(-0.178,-0.016) | -3.16 |
| Age-standardized | Incidence | Rate | Comoros | Both | Encephalitis | 12.5 | 14 | 11.3 | 11.3 | 12.7 | 10.1 | -0.378(-0.402,-0.355) | -9.38 |
| Age-standardized | Incidence | Rate | Congo | Both | Encephalitis | 9.8 | 11.2 | 8.6 | 9.1 | 10.3 | 7.9 | -0.251(-0.290,-0.213) | -7.46 |
| Age-standardized | Incidence | Rate | Cook Islands | Both | Encephalitis | 10.1 | 11.9 | 8.6 | 9.6 | 11.3 | 8.2 | -0.160(-0.202,-0.118) | -4.44 |
| Age-standardized | Incidence | Rate | Costa Rica | Both | Encephalitis | 20 | 23.2 | 17.6 | 15.1 | 17.5 | 13.1 | -0.861(-0.999,-0.722) | -24.38 |
| Age-standardized | Incidence | Rate | Croatia | Both | Encephalitis | 8.5 | 9.7 | 7.4 | 8.7 | 9.6 | 7.7 | 0.170(0.046,0.293) | 1.63 |
| Age-standardized | Incidence | Rate | Cuba | Both | Encephalitis | 14.9 | 17 | 13.1 | 11 | 12.8 | 9.5 | -0.848(-1.008,-0.687) | -26.18 |
| Age-standardized | Incidence | Rate | Cyprus | Both | Encephalitis | 2.7 | 3.1 | 2.4 | 2.3 | 2.6 | 2 | -0.389(-0.764,-0.011) | -14.47 |
| Age-standardized | Incidence | Rate | Czechia | Both | Encephalitis | 6.2 | 7.1 | 5.3 | 5.7 | 6.6 | 5 | -0.210(-0.248,-0.172) | -7.39 |
| Age-standardized | Incidence | Rate | Cote d'Ivoire | Both | Encephalitis | 14 | 15.8 | 12.5 | 13.4 | 15 | 12 | -0.083(-0.114,-0.051) | -4.19 |
| Age-standardized | Incidence | Rate | Democratic People's Republic of Korea | Both | Encephalitis | 13.7 | 16.1 | 11.8 | 12.1 | 14.3 | 10.4 | -0.431(-0.439,-0.422) | -11.8 |
| Age-standardized | Incidence | Rate | Democratic Republic of the Congo | Both | Encephalitis | 9.8 | 11.2 | 8.5 | 9 | 10.3 | 7.9 | -0.261(-0.299,-0.222) | -7.87 |
| Age-standardized | Incidence | Rate | Denmark | Both | Encephalitis | 2.7 | 3.1 | 2.3 | 2.9 | 3.3 | 2.5 | 0.114(-0.518,0.750) | 7 |
| Age-standardized | Incidence | Rate | Djibouti | Both | Encephalitis | 12.2 | 13.6 | 10.9 | 11.7 | 13.1 | 10.5 | -0.189(-0.226,-0.151) | -4.07 |
| Age-standardized | Incidence | Rate | Dominica | Both | Encephalitis | 16.6 | 18.7 | 14.9 | 14.5 | 16.3 | 12.9 | -0.427(-0.569,-0.285) | -13.18 |
| Age-standardized | Incidence | Rate | Dominican Republic | Both | Encephalitis | 12.9 | 14.7 | 11.4 | 12 | 13.5 | 10.6 | -0.175(-0.256,-0.093) | -6.98 |
| Age-standardized | Incidence | Rate | Ecuador | Both | Encephalitis | 9.4 | 10.4 | 8.5 | 7.4 | 8.1 | 6.9 | -0.965(-1.075,-0.855) | -20.49 |
| Age-standardized | Incidence | Rate | Egypt | Both | Encephalitis | 7.9 | 9 | 7 | 7.5 | 8.5 | 6.6 | -0.039(-0.110,0.031) | -5.37 |
| Age-standardized | Incidence | Rate | El Salvador | Both | Encephalitis | 16.8 | 19.4 | 14.8 | 13.2 | 15.7 | 11.4 | -0.677(-0.759,-0.596) | -21.4 |
| Age-standardized | Incidence | Rate | Equatorial Guinea | Both | Encephalitis | 10 | 11.4 | 8.7 | 9.1 | 10.4 | 7.8 | -0.297(-0.316,-0.278) | -8.88 |
| Age-standardized | Incidence | Rate | Eritrea | Both | Encephalitis | 13 | 14.4 | 11.6 | 11.5 | 12.9 | 10.3 | -0.431(-0.454,-0.408) | -11.21 |
| Age-standardized | Incidence | Rate | Estonia | Both | Encephalitis | 9.3 | 10.7 | 8.1 | 8.7 | 10 | 7.6 | -0.230(-0.290,-0.169) | -6.45 |
| Age-standardized | Incidence | Rate | Eswatini | Both | Encephalitis | 10.2 | 11.6 | 8.9 | 9.6 | 11.1 | 8.4 | -0.177(-0.229,-0.126) | -5.32 |
| Age-standardized | Incidence | Rate | Ethiopia | Both | Encephalitis | 13.5 | 15.1 | 12.1 | 11.2 | 12.5 | 10 | -0.675(-0.701,-0.648) | -17.03 |
| Age-standardized | Incidence | Rate | Fiji | Both | Encephalitis | 13.5 | 15.4 | 11.9 | 12.5 | 14.1 | 11.1 | -0.396(-0.450,-0.341) | -7.48 |
| Age-standardized | Incidence | Rate | Finland | Both | Encephalitis | 6.8 | 7.8 | 5.9 | 6.8 | 7.7 | 5.9 | 0.614(0.294,0.935) | -0.47 |
| Age-standardized | Incidence | Rate | France | Both | Encephalitis | 1.8 | 2 | 1.6 | 1.9 | 2.1 | 1.6 | -0.252(-0.429,-0.074) | 4.73 |
| Age-standardized | Incidence | Rate | Gabon | Both | Encephalitis | 10 | 11.3 | 8.6 | 9.2 | 10.5 | 8 | -0.252(-0.288,-0.216) | -7.19 |
| Age-standardized | Incidence | Rate | Gambia | Both | Encephalitis | 13.8 | 15.6 | 12.3 | 13.7 | 15.4 | 12.3 | -0.051(-0.072,-0.030) | -1.22 |
| Age-standardized | Incidence | Rate | Georgia | Both | Encephalitis | 11 | 12.5 | 9.5 | 9.8 | 10.8 | 8.9 | -0.450(-0.559,-0.340) | -10.84 |
| Age-standardized | Incidence | Rate | Germany | Both | Encephalitis | 4.8 | 5.5 | 4.1 | 5 | 5.7 | 4.4 | 0.804(0.315,1.295) | 5.11 |
| Age-standardized | Incidence | Rate | Ghana | Both | Encephalitis | 15.8 | 17.6 | 14.1 | 15.7 | 17.4 | 14.2 | 0.000(-0.008,0.008) | -0.37 |
| Age-standardized | Incidence | Rate | Greece | Both | Encephalitis | 8.7 | 9.9 | 7.6 | 10 | 11.3 | 8.8 | 0.975(0.680,1.271) | 14.6 |
| Age-standardized | Incidence | Rate | Greenland | Both | Encephalitis | 0.7 | 0.8 | 0.6 | 1 | 1.1 | 0.9 | 2.076(1.458,2.698) | 49.98 |
| Age-standardized | Incidence | Rate | Grenada | Both | Encephalitis | 15.7 | 17.6 | 14 | 11.8 | 13.4 | 10.4 | -0.933(-1.087,-0.778) | -25.01 |
| Age-standardized | Incidence | Rate | Guam | Both | Encephalitis | 10.1 | 11.8 | 8.7 | 9.7 | 11.4 | 8.3 | -0.144(-0.184,-0.104) | -4.59 |
| Age-standardized | Incidence | Rate | Guatemala | Both | Encephalitis | 15.3 | 17.7 | 13.4 | 13.1 | 15.4 | 11.4 | -0.530(-0.551,-0.509) | -14.36 |
| Age-standardized | Incidence | Rate | Guinea | Both | Encephalitis | 14.5 | 16.2 | 13 | 13.8 | 15.4 | 12.5 | -0.102(-0.122,-0.081) | -4.59 |
| Age-standardized | Incidence | Rate | Guinea-Bissau | Both | Encephalitis | 14.5 | 16.2 | 13 | 13.5 | 15.1 | 12.2 | -0.165(-0.206,-0.123) | -6.89 |
| Age-standardized | Incidence | Rate | Guyana | Both | Encephalitis | 18 | 19.9 | 16.4 | 14.6 | 16.2 | 13.2 | -0.643(-0.816,-0.470) | -18.75 |
| Age-standardized | Incidence | Rate | Haiti | Both | Encephalitis | 14.4 | 16.1 | 12.9 | 12.4 | 13.9 | 11.1 | -0.480(-0.550,-0.410) | -13.56 |
| Age-standardized | Incidence | Rate | Honduras | Both | Encephalitis | 15.7 | 18 | 13.8 | 13.8 | 16.1 | 11.9 | -0.379(-0.418,-0.340) | -11.62 |
| Age-standardized | Incidence | Rate | Hungary | Both | Encephalitis | 6.3 | 7.2 | 5.5 | 5.7 | 6.5 | 4.9 | -0.327(-0.358,-0.297) | -10.25 |
| Age-standardized | Incidence | Rate | Iceland | Both | Encephalitis | 0.6 | 0.7 | 0.5 | 0.6 | 0.7 | 0.5 | 0.621(0.137,1.106) | 6.25 |
| Age-standardized | Incidence | Rate | India | Both | Encephalitis | 80.9 | 87.6 | 75.1 | 53.5 | 57.8 | 49.8 | -1.813(-2.032,-1.593) | -33.93 |
| Age-standardized | Incidence | Rate | Indonesia | Both | Encephalitis | 10.8 | 12.9 | 9 | 8.3 | 9.6 | 7.2 | -1.341(-1.572,-1.110) | -23.45 |
| Age-standardized | Incidence | Rate | Iran (Islamic Republic of) | Both | Encephalitis | 6.9 | 7.9 | 6 | 6.4 | 7.5 | 5.6 | -0.189(-0.230,-0.147) | -6.86 |
| Age-standardized | Incidence | Rate | Iraq | Both | Encephalitis | 12.7 | 14.1 | 11.5 | 10.9 | 12.1 | 9.8 | -0.617(-0.677,-0.557) | -14.63 |
| Age-standardized | Incidence | Rate | Ireland | Both | Encephalitis | 3.3 | 3.8 | 2.8 | 3.2 | 3.7 | 2.8 | -0.018(-0.032,-0.003) | -1.28 |
| Age-standardized | Incidence | Rate | Israel | Both | Encephalitis | 3.5 | 4 | 3 | 3.6 | 4 | 3.1 | -0.060(-0.149,0.029) | 2.55 |
| Age-standardized | Incidence | Rate | Italy | Both | Encephalitis | 7.1 | 8.2 | 6.2 | 7 | 7.7 | 6.5 | -0.131(-0.553,0.292) | -0.45 |
| Age-standardized | Incidence | Rate | Jamaica | Both | Encephalitis | 15.8 | 17.8 | 14.3 | 12.9 | 14.7 | 11.4 | -0.576(-0.733,-0.419) | -18.55 |
| Age-standardized | Incidence | Rate | Japan | Both | Encephalitis | 8.1 | 9.8 | 6.8 | 7.6 | 9.1 | 6.4 | 0.188(-0.040,0.417) | -6.82 |
| Age-standardized | Incidence | Rate | Jordan | Both | Encephalitis | 7.3 | 8.5 | 6.4 | 6.4 | 7.4 | 5.5 | -0.509(-0.583,-0.435) | -12.94 |
| Age-standardized | Incidence | Rate | Kazakhstan | Both | Encephalitis | 15.4 | 17.1 | 13.7 | 12.9 | 14.5 | 11.7 | -1.039(-1.261,-0.816) | -15.92 |
| Age-standardized | Incidence | Rate | Kenya | Both | Encephalitis | 12.7 | 14.2 | 11.3 | 12.8 | 14.2 | 11.5 | 0.323(0.207,0.440) | 0.53 |
| Age-standardized | Incidence | Rate | Kiribati | Both | Encephalitis | 12.3 | 14.1 | 10.8 | 12.3 | 14 | 10.8 | 0.110(0.026,0.195) | 0.01 |
| Age-standardized | Incidence | Rate | Kuwait | Both | Encephalitis | 7 | 8.1 | 6.1 | 6.5 | 7.5 | 5.6 | -0.206(-0.249,-0.162) | -7.62 |
| Age-standardized | Incidence | Rate | Kyrgyzstan | Both | Encephalitis | 14 | 15.6 | 12.5 | 11.3 | 12.7 | 10 | -0.910(-1.038,-0.782) | -19.06 |
| Age-standardized | Incidence | Rate | Lao People's Democratic Republic | Both | Encephalitis | 12.9 | 14.4 | 11.4 | 11.2 | 12.5 | 10.1 | -0.491(-0.540,-0.441) | -12.74 |
| Age-standardized | Incidence | Rate | Latvia | Both | Encephalitis | 12.4 | 13.6 | 11.2 | 9.9 | 11.1 | 8.8 | -0.920(-0.975,-0.864) | -20.23 |
| Age-standardized | Incidence | Rate | Lebanon | Both | Encephalitis | 7.1 | 8.2 | 6.2 | 6.8 | 7.8 | 5.9 | -0.130(-0.162,-0.098) | -4.4 |
| Age-standardized | Incidence | Rate | Lesotho | Both | Encephalitis | 10 | 11.5 | 8.8 | 9.6 | 11.1 | 8.3 | -0.139(-0.189,-0.089) | -4.55 |
| Age-standardized | Incidence | Rate | Liberia | Both | Encephalitis | 14.8 | 16.5 | 13.1 | 13.2 | 14.8 | 11.9 | -0.318(-0.385,-0.251) | -10.64 |
| Age-standardized | Incidence | Rate | Libya | Both | Encephalitis | 5.5 | 6.4 | 4.8 | 5.3 | 6.2 | 4.7 | -0.091(-0.107,-0.074) | -3.33 |
| Age-standardized | Incidence | Rate | Lithuania | Both | Encephalitis | 10.1 | 11.4 | 8.9 | 9.1 | 10.4 | 8.1 | -0.363(-0.419,-0.307) | -9.53 |
| Age-standardized | Incidence | Rate | Luxembourg | Both | Encephalitis | 4.8 | 5.5 | 4.2 | 4.8 | 5.4 | 4.2 | -0.265(-0.465,-0.064) | -0.11 |
| Age-standardized | Incidence | Rate | Madagascar | Both | Encephalitis | 12 | 13.3 | 10.6 | 10.8 | 12.1 | 9.6 | -0.334(-0.354,-0.313) | -9.39 |
| Age-standardized | Incidence | Rate | Malawi | Both | Encephalitis | 11.9 | 13.2 | 10.5 | 11.1 | 12.3 | 9.9 | -0.234(-0.259,-0.209) | -6.85 |
| Age-standardized | Incidence | Rate | Malaysia | Both | Encephalitis | 16.6 | 18.4 | 15 | 16.4 | 18.1 | 14.8 | -0.015(-0.055,0.026) | -1.54 |
| Age-standardized | Incidence | Rate | Maldives | Both | Encephalitis | 13.8 | 15.6 | 12.4 | 11.6 | 13.2 | 10.4 | -0.578(-0.669,-0.488) | -15.77 |
| Age-standardized | Incidence | Rate | Mali | Both | Encephalitis | 15.9 | 17.8 | 14.5 | 15.4 | 16.9 | 14 | -0.067(-0.150,0.016) | -3.42 |
| Age-standardized | Incidence | Rate | Malta | Both | Encephalitis | 1.6 | 1.8 | 1.4 | 1.8 | 2 | 1.6 | 0.667(0.372,0.963) | 10.88 |
| Age-standardized | Incidence | Rate | Marshall Islands | Both | Encephalitis | 10.4 | 12.2 | 9 | 9.8 | 11.5 | 8.5 | -0.207(-0.239,-0.175) | -5.81 |
| Age-standardized | Incidence | Rate | Mauritania | Both | Encephalitis | 15 | 16.8 | 13.3 | 13.7 | 15.4 | 12.4 | -0.257(-0.280,-0.235) | -8.52 |
| Age-standardized | Incidence | Rate | Mauritius | Both | Encephalitis | 10.7 | 12.3 | 9.4 | 12.2 | 13.8 | 10.9 | 0.560(0.517,0.602) | 14.52 |
| Age-standardized | Incidence | Rate | Mexico | Both | Encephalitis | 15.3 | 17.8 | 13.3 | 15 | 17.3 | 13.1 | -0.650(-0.834,-0.465) | -1.94 |
| Age-standardized | Incidence | Rate | Micronesia (Federated States of) | Both | Encephalitis | 10.5 | 12.3 | 9.1 | 9.8 | 11.4 | 8.5 | -0.228(-0.246,-0.209) | -6.54 |
| Age-standardized | Incidence | Rate | Monaco | Both | Encephalitis | 3.1 | 3.6 | 2.7 | 3.1 | 3.6 | 2.7 | -0.003(-0.017,0.012) | -0.27 |
| Age-standardized | Incidence | Rate | Mongolia | Both | Encephalitis | 15.1 | 16.8 | 13.6 | 13.3 | 14.7 | 12 | -0.460(-0.483,-0.437) | -11.93 |
| Age-standardized | Incidence | Rate | Montenegro | Both | Encephalitis | 6.1 | 7.1 | 5.3 | 5.7 | 6.5 | 4.9 | -0.212(-0.247,-0.176) | -6.59 |
| Age-standardized | Incidence | Rate | Morocco | Both | Encephalitis | 7 | 8.1 | 6.1 | 6.7 | 7.8 | 5.8 | -0.147(-0.187,-0.108) | -4.72 |
| Age-standardized | Incidence | Rate | Mozambique | Both | Encephalitis | 12.5 | 13.9 | 11.2 | 12.1 | 13.4 | 11 | -0.072(-0.094,-0.050) | -2.62 |
| Age-standardized | Incidence | Rate | Myanmar | Both | Encephalitis | 11.4 | 13 | 10 | 10.1 | 11.4 | 9 | -0.403(-0.491,-0.316) | -11.37 |
| Age-standardized | Incidence | Rate | Namibia | Both | Encephalitis | 10.4 | 11.9 | 9.1 | 9.8 | 11.3 | 8.6 | -0.189(-0.226,-0.152) | -5.49 |
| Age-standardized | Incidence | Rate | Nauru | Both | Encephalitis | 10.1 | 11.8 | 8.7 | 9.8 | 11.4 | 8.5 | -0.054(-0.123,0.015) | -2.92 |
| Age-standardized | Incidence | Rate | Nepal | Both | Encephalitis | 55.4 | 61 | 51.1 | 41.2 | 44.6 | 38.3 | -1.157(-1.246,-1.069) | -25.6 |
| Age-standardized | Incidence | Rate | Netherlands | Both | Encephalitis | 2.5 | 2.9 | 2.2 | 2.5 | 2.8 | 2.2 | -0.145(-0.331,0.041) | -0.91 |
| Age-standardized | Incidence | Rate | New Zealand | Both | Encephalitis | 4.8 | 5.6 | 4.2 | 5 | 5.6 | 4.5 | 0.109(0.087,0.130) | 2.63 |
| Age-standardized | Incidence | Rate | Nicaragua | Both | Encephalitis | 16.2 | 18.8 | 14.2 | 15.1 | 17.6 | 13.2 | -0.243(-0.262,-0.223) | -7.01 |
| Age-standardized | Incidence | Rate | Niger | Both | Encephalitis | 17.1 | 19.1 | 15.3 | 13.6 | 15.1 | 12.2 | -0.764(-0.866,-0.662) | -20.73 |
| Age-standardized | Incidence | Rate | Nigeria | Both | Encephalitis | 15 | 16.9 | 13.4 | 13.8 | 15.5 | 12.4 | -0.206(-0.248,-0.164) | -7.82 |
| Age-standardized | Incidence | Rate | Niue | Both | Encephalitis | 10.6 | 12.3 | 9.1 | 10 | 11.7 | 8.6 | -0.185(-0.208,-0.162) | -5.05 |
| Age-standardized | Incidence | Rate | North Macedonia | Both | Encephalitis | 6.3 | 7.1 | 5.5 | 5.7 | 6.5 | 5 | -0.266(-0.298,-0.234) | -9.56 |
| Age-standardized | Incidence | Rate | Northern Mariana Islands | Both | Encephalitis | 11 | 12.9 | 9.5 | 10.6 | 12.4 | 9.2 | -0.157(-0.178,-0.137) | -3.54 |
| Age-standardized | Incidence | Rate | Norway | Both | Encephalitis | 3.9 | 4.4 | 3.4 | 3.6 | 4.1 | 3.1 | -0.010(-0.120,0.100) | -8.02 |
| Age-standardized | Incidence | Rate | Oman | Both | Encephalitis | 24.1 | 26.1 | 22.2 | 11.4 | 12.6 | 10.4 | -2.890(-3.243,-2.536) | -52.65 |
| Age-standardized | Incidence | Rate | Pakistan | Both | Encephalitis | 56.3 | 61.6 | 51.9 | 54.8 | 59.6 | 50.7 | -0.093(-0.165,-0.022) | -2.8 |
| Age-standardized | Incidence | Rate | Palau | Both | Encephalitis | 10.4 | 12.1 | 8.9 | 9.8 | 11.5 | 8.4 | -0.177(-0.206,-0.149) | -5.46 |
| Age-standardized | Incidence | Rate | Palestine | Both | Encephalitis | 6.9 | 8 | 5.9 | 6.6 | 7.6 | 5.7 | -0.130(-0.195,-0.065) | -4.99 |
| Age-standardized | Incidence | Rate | Panama | Both | Encephalitis | 16.8 | 19.4 | 14.7 | 13.9 | 16.2 | 12 | -0.543(-0.598,-0.488) | -17.5 |
| Age-standardized | Incidence | Rate | Papua New Guinea | Both | Encephalitis | 11.4 | 13.3 | 10 | 11.2 | 13 | 9.7 | -0.063(-0.091,-0.034) | -1.84 |
| Age-standardized | Incidence | Rate | Paraguay | Both | Encephalitis | 6.9 | 7.8 | 6 | 6.4 | 7.2 | 5.6 | -0.255(-0.269,-0.242) | -7.26 |
| Age-standardized | Incidence | Rate | Peru | Both | Encephalitis | 12.5 | 13.8 | 11.4 | 10.7 | 11.8 | 9.6 | -0.704(-0.807,-0.601) | -14.86 |
| Age-standardized | Incidence | Rate | Philippines | Both | Encephalitis | 19.4 | 21.9 | 17.2 | 17.5 | 19.6 | 15.6 | -0.331(-0.409,-0.252) | -9.63 |
| Age-standardized | Incidence | Rate | Poland | Both | Encephalitis | 4.1 | 4.9 | 3.5 | 1.7 | 1.9 | 1.5 | -2.997(-3.372,-2.621) | -58.09 |
| Age-standardized | Incidence | Rate | Portugal | Both | Encephalitis | 1.1 | 1.2 | 1 | 1.3 | 1.5 | 1.2 | 0.487(-0.328,1.309) | 22.61 |
| Age-standardized | Incidence | Rate | Puerto Rico | Both | Encephalitis | 13.6 | 15.5 | 12 | 10.7 | 12.6 | 9.3 | -0.663(-0.782,-0.545) | -20.95 |
| Age-standardized | Incidence | Rate | Qatar | Both | Encephalitis | 7.8 | 9 | 7 | 6.7 | 7.7 | 5.8 | -0.611(-0.673,-0.549) | -14.82 |
| Age-standardized | Incidence | Rate | Republic of Korea | Both | Encephalitis | 4.5 | 5.4 | 3.8 | 4.8 | 5.6 | 4.1 | 0.136(0.054,0.219) | 5.08 |
| Age-standardized | Incidence | Rate | Republic of Moldova | Both | Encephalitis | 9.4 | 10.7 | 8.3 | 9.2 | 10.4 | 8.1 | -0.098(-0.205,0.010) | -2.15 |
| Age-standardized | Incidence | Rate | Romania | Both | Encephalitis | 10.5 | 11.5 | 9.4 | 7.3 | 8.3 | 6.4 | -1.223(-1.286,-1.159) | -30.23 |
| Age-standardized | Incidence | Rate | Russian Federation | Both | Encephalitis | 11.6 | 12.9 | 10.4 | 9.8 | 10.9 | 8.8 | -0.645(-0.721,-0.570) | -15.38 |
| Age-standardized | Incidence | Rate | Rwanda | Both | Encephalitis | 11.9 | 13.4 | 10.6 | 11 | 12.3 | 9.8 | -0.356(-0.394,-0.319) | -8 |
| Age-standardized | Incidence | Rate | Saint Kitts and Nevis | Both | Encephalitis | 19.9 | 22 | 17.9 | 12.9 | 14.6 | 11.3 | -1.341(-1.607,-1.074) | -35.17 |
| Age-standardized | Incidence | Rate | Saint Lucia | Both | Encephalitis | 17.5 | 19.6 | 15.7 | 12.7 | 14.4 | 11.2 | -1.029(-1.241,-0.818) | -27.79 |
| Age-standardized | Incidence | Rate | Saint Vincent and the Grenadines | Both | Encephalitis | 14.3 | 16.2 | 12.8 | 11.3 | 12.9 | 9.9 | -0.747(-0.846,-0.647) | -21.37 |
| Age-standardized | Incidence | Rate | Samoa | Both | Encephalitis | 10.6 | 12.3 | 9.2 | 10.1 | 11.9 | 8.7 | -0.173(-0.194,-0.153) | -4.89 |
| Age-standardized | Incidence | Rate | San Marino | Both | Encephalitis | 4.6 | 5.1 | 4.1 | 5.1 | 5.7 | 4.6 | 0.489(0.385,0.593) | 12.72 |
| Age-standardized | Incidence | Rate | Sao Tome and Principe | Both | Encephalitis | 19.2 | 21.2 | 17.5 | 19.1 | 21 | 17.5 | 0.024(-0.061,0.109) | -0.42 |
| Age-standardized | Incidence | Rate | Saudi Arabia | Both | Encephalitis | 10.7 | 11.9 | 9.7 | 8.4 | 9.4 | 7.5 | -1.006(-1.103,-0.909) | -21.51 |
| Age-standardized | Incidence | Rate | Senegal | Both | Encephalitis | 14.9 | 16.8 | 13.4 | 14.4 | 16.1 | 12.9 | -0.058(-0.092,-0.024) | -3.72 |
| Age-standardized | Incidence | Rate | Serbia | Both | Encephalitis | 8 | 9 | 7.1 | 7.5 | 8.5 | 6.7 | -0.239(-0.298,-0.180) | -5.59 |
| Age-standardized | Incidence | Rate | Seychelles | Both | Encephalitis | 11.2 | 12.9 | 9.9 | 11.8 | 13.2 | 10.5 | 0.109(0.062,0.155) | 5.03 |
| Age-standardized | Incidence | Rate | Sierra Leone | Both | Encephalitis | 14.6 | 16.3 | 13 | 13.3 | 14.9 | 11.9 | -0.279(-0.318,-0.240) | -8.76 |
| Age-standardized | Incidence | Rate | Singapore | Both | Encephalitis | 2.7 | 3 | 2.4 | 2.6 | 3 | 2.3 | -0.331(-0.729,0.068) | -1.65 |
| Age-standardized | Incidence | Rate | Slovakia | Both | Encephalitis | 8.2 | 9.3 | 7 | 7.5 | 8.6 | 6.6 | -0.046(-0.339,0.247) | -7.58 |
| Age-standardized | Incidence | Rate | Slovenia | Both | Encephalitis | 6.7 | 7.6 | 5.9 | 6.5 | 7.4 | 5.7 | -0.119(-0.169,-0.070) | -2.82 |
| Age-standardized | Incidence | Rate | Solomon Islands | Both | Encephalitis | 10.1 | 11.8 | 8.7 | 9.6 | 11.2 | 8.3 | -0.159(-0.193,-0.125) | -4.46 |
| Age-standardized | Incidence | Rate | Somalia | Both | Encephalitis | 11.9 | 13.3 | 10.6 | 11.5 | 12.8 | 10.3 | -0.140(-0.162,-0.118) | -3.38 |
| Age-standardized | Incidence | Rate | South Africa | Both | Encephalitis | 10.4 | 11.9 | 9 | 9.3 | 10.7 | 8.1 | -0.401(-0.542,-0.261) | -10.7 |
| Age-standardized | Incidence | Rate | South Sudan | Both | Encephalitis | 12.2 | 13.6 | 10.9 | 11.2 | 12.5 | 10 | -0.272(-0.288,-0.255) | -8.05 |
| Age-standardized | Incidence | Rate | Spain | Both | Encephalitis | 2.9 | 3.3 | 2.5 | 4.1 | 4.6 | 3.6 | 1.538(1.137,1.942) | 40.57 |
| Age-standardized | Incidence | Rate | Sri Lanka | Both | Encephalitis | 19.5 | 21.4 | 17.7 | 16.4 | 18.1 | 15 | -0.665(-0.712,-0.617) | -15.61 |
| Age-standardized | Incidence | Rate | Sudan | Both | Encephalitis | 7.1 | 8.1 | 6.2 | 6.6 | 7.5 | 5.8 | -0.239(-0.272,-0.206) | -7.23 |
| Age-standardized | Incidence | Rate | Suriname | Both | Encephalitis | 17.9 | 19.9 | 16.1 | 13.8 | 15.5 | 12.4 | -0.767(-0.918,-0.615) | -22.73 |
| Age-standardized | Incidence | Rate | Sweden | Both | Encephalitis | 8.3 | 9.5 | 7.3 | 8.5 | 10 | 7.4 | 0.456(0.301,0.612) | 3.01 |
| Age-standardized | Incidence | Rate | Switzerland | Both | Encephalitis | 5 | 5.8 | 4.4 | 5.5 | 6.2 | 4.8 | 0.260(-0.061,0.581) | 8.1 |
| Age-standardized | Incidence | Rate | Syrian Arab Republic | Both | Encephalitis | 8.2 | 9.2 | 7.3 | 7.1 | 8.2 | 6.2 | -0.597(-0.643,-0.551) | -13.39 |
| Age-standardized | Incidence | Rate | Taiwan (Province of China) | Both | Encephalitis | 8.4 | 10.1 | 7.1 | 6.6 | 7.7 | 5.7 | -0.898(-1.138,-0.659) | -22.46 |
| Age-standardized | Incidence | Rate | Tajikistan | Both | Encephalitis | 11 | 12.7 | 9.3 | 9.9 | 11.4 | 8.5 | -0.297(-0.378,-0.217) | -9.53 |
| Age-standardized | Incidence | Rate | Thailand | Both | Encephalitis | 16.4 | 18.4 | 14.8 | 12.9 | 14.5 | 11.6 | -0.973(-1.052,-0.894) | -21.32 |
| Age-standardized | Incidence | Rate | Timor-Leste | Both | Encephalitis | 14.1 | 15.9 | 12.5 | 12.4 | 13.8 | 11.1 | -0.525(-0.570,-0.480) | -11.89 |
| Age-standardized | Incidence | Rate | Togo | Both | Encephalitis | 13.9 | 15.7 | 12.3 | 13.1 | 14.7 | 11.7 | -0.188(-0.205,-0.171) | -5.51 |
| Age-standardized | Incidence | Rate | Tokelau | Both | Encephalitis | 10.7 | 12.4 | 9.2 | 10 | 11.8 | 8.6 | -0.197(-0.222,-0.173) | -5.74 |
| Age-standardized | Incidence | Rate | Tonga | Both | Encephalitis | 12 | 13.8 | 10.5 | 11.8 | 13.5 | 10.3 | -0.060(-0.064,-0.056) | -1.59 |
| Age-standardized | Incidence | Rate | Trinidad and Tobago | Both | Encephalitis | 16.8 | 18.8 | 14.9 | 11.1 | 12.9 | 9.7 | -1.563(-1.723,-1.403) | -33.99 |
| Age-standardized | Incidence | Rate | Tunisia | Both | Encephalitis | 7.1 | 8.2 | 6.2 | 6.7 | 7.8 | 5.9 | -0.163(-0.183,-0.143) | -5.29 |
| Age-standardized | Incidence | Rate | Turkey | Both | Encephalitis | 9.4 | 10.9 | 8.2 | 8.1 | 9.2 | 7 | -0.790(-0.964,-0.616) | -13.88 |
| Age-standardized | Incidence | Rate | Turkmenistan | Both | Encephalitis | 11.2 | 12.7 | 9.7 | 14.1 | 15.5 | 12.8 | 0.823(0.646,1.001) | 25.71 |
| Age-standardized | Incidence | Rate | Tuvalu | Both | Encephalitis | 10.4 | 12.2 | 9 | 9.7 | 11.3 | 8.4 | -0.206(-0.235,-0.176) | -6.35 |
| Age-standardized | Incidence | Rate | Uganda | Both | Encephalitis | 11.7 | 13.1 | 10.4 | 11.1 | 12.4 | 9.9 | -0.207(-0.239,-0.176) | -5.68 |
| Age-standardized | Incidence | Rate | Ukraine | Both | Encephalitis | 12.9 | 14.3 | 11.5 | 13.9 | 15.3 | 12.7 | 0.278(0.238,0.317) | 8.34 |
| Age-standardized | Incidence | Rate | United Arab Emirates | Both | Encephalitis | 7 | 8.1 | 6.2 | 6.7 | 7.7 | 5.9 | -0.151(-0.170,-0.132) | -4.57 |
| Age-standardized | Incidence | Rate | United Kingdom | Both | Encephalitis | 4.8 | 5.8 | 3.9 | 6.7 | 7.8 | 5.7 | 0.810(0.594,1.025) | 39.56 |
| Age-standardized | Incidence | Rate | United Republic of Tanzania | Both | Encephalitis | 12.3 | 13.8 | 10.9 | 11.6 | 12.9 | 10.3 | -0.195(-0.222,-0.168) | -5.76 |
| Age-standardized | Incidence | Rate | United States of America | Both | Encephalitis | 1.9 | 2.3 | 1.6 | 1.8 | 2 | 1.6 | -0.471(-0.552,-0.389) | -9.1 |
| Age-standardized | Incidence | Rate | United States Virgin Islands | Both | Encephalitis | 11.8 | 13.6 | 10.2 | 10.6 | 12.4 | 9.2 | -0.318(-0.342,-0.294) | -9.83 |
| Age-standardized | Incidence | Rate | Uruguay | Both | Encephalitis | 6.5 | 7.4 | 5.7 | 7.2 | 8.1 | 6.4 | 0.129(-0.032,0.290) | 10.92 |
| Age-standardized | Incidence | Rate | Uzbekistan | Both | Encephalitis | 17.4 | 19.1 | 15.8 | 16.7 | 18.2 | 15.4 | -0.086(-0.163,-0.009) | -3.87 |
| Age-standardized | Incidence | Rate | Vanuatu | Both | Encephalitis | 10.3 | 12 | 8.9 | 9.9 | 11.6 | 8.5 | -0.121(-0.148,-0.094) | -3.56 |
| Age-standardized | Incidence | Rate | Venezuela (Bolivarian Republic of) | Both | Encephalitis | 17.3 | 20 | 15.1 | 14 | 16.4 | 12.1 | -0.625(-0.709,-0.541) | -18.93 |
| Age-standardized | Incidence | Rate | Viet Nam | Both | Encephalitis | 22.2 | 24.7 | 20.2 | 20 | 21.9 | 18.4 | -0.441(-0.512,-0.371) | -9.78 |
| Age-standardized | Incidence | Rate | Yemen | Both | Encephalitis | 7 | 8 | 6.1 | 6.6 | 7.6 | 5.8 | -0.139(-0.191,-0.087) | -5.44 |
| Age-standardized | Incidence | Rate | Zambia | Both | Encephalitis | 11.9 | 13.2 | 10.6 | 11.8 | 13.1 | 10.7 | 0.013(-0.003,0.029) | -0.47 |
| Age-standardized | Incidence | Rate | Zimbabwe | Both | Encephalitis | 11.5 | 13 | 10.3 | 13.3 | 14.8 | 12 | 0.492(0.379,0.606) | 15.51 |

*ASIR* age-standardized incidence rate, *EAPC* estimated annual percentage change, *CI* confidence interval, *UI* uncertainty interval
