# Supplementary material for: Socioeconomic drivers of encephalitis burden in the post-COVID era: a 204-country analysis from global burden of disease study 2021
Source: Front Public Health. 2025 Sep 18;13:1651734. doi: 10.3389/fpubh.2025.1651734 (PMC12488571; doi:10.3389/fpubh.2025.1651734)
Supplement: SUPPLEMENTARY FIGURE S4 — (A) Age-standardized prevalence rates of encephalitis for 21 regions by SDI from 1990–2021. The expected values based on the SDI and disease rates at all of the locations are shown as black lines. (B) Age-standardized prevalence rates for encephalitis in 204 countries and territories by SDI in 2021. Expected values based on the sociodemographic index and disease rate at all of the locations are shown as black lines. SDI, sociodemographic index. [file Data_Sheet_4.PDF]

Table S4: The ASDR of encephalitis in 204 countries and territories in 1990 and 2021 and EAPC of ASDR for encephalitis in 204 countries and territories from 1990 to 2021

age_name	measure_name	metric_nam	location_name	sex_name	cause_name	1990 ASDR	1990 ASDR 95%UI upper	1990 ASDR 95%UI lower	2021 ASDR	2021 ASDR 95%UI upper	2021 ASDR 95%UI lower	EAPC (95%CI)
Age-standardized	DALYs	Rate	Afghanistan	Both	Encephalitis	36.1	63.9	21.6	34.3	49.1	24.2	0.084(-0.058,0.227)
Age-standardized	DALYs	Rate	Albania	Both	Encephalitis	102.9	131.2	76.9	40.9	62.4	25	-4.137(-4.665,-3.606)
Age-standardized	DALYs	Rate	Algeria	Both	Encephalitis	18.2	28.8	13.2	15	20.1	11.5	-0.570(-0.658,-0.482)
Age-standardized	DALYs	Rate	American Samoa	Both	Encephalitis	4.2	5.4	3	3.7	4.8	2.7	-0.676(-0.898,-0.454)
Age-standardized	DALYs	Rate	Andorra	Both	Encephalitis	10.5	14.6	7.4	6.3	8.5	4.6	-1.222(-1.382,-1.062)
Age-standardized	DALYs	Rate	Angola	Both	Encephalitis	39.6	69.3	24.9	29.2	39.9	20.6	-1.031(-1.107,-0.954)
Age-standardized	DALYs	Rate	Antigua and Barbuda	Both	Encephalitis	13.8	16	11.9	8.5	9.7	7.3	-1.734(-3.113,-0.336)
Age-standardized	DALYs	Rate	Argentina	Both	Encephalitis	24.9	26.6	23.4	20.5	22.4	18.6	-0.500(-1.091,0.095)
Age-standardized	DALYs	Rate	Armenia	Both	Encephalitis	26	35.2	19	26.9	36	19.3	0.402(0.094,0.710)
Age-standardized	DALYs	Rate	Australia	Both	Encephalitis	3.5	3.9	3.3	6.8	7.5	6.1	2.535(2.047,3.025)
Age-standardized	DALYs	Rate	Austria	Both	Encephalitis	13.3	14.3	12.2	10.3	11.4	9.4	-0.599(-1.088,-0.108)
Age-standardized	DALYs	Rate	Azerbaijan	Both	Encephalitis	116.8	188.5	69.6	103.3	191.7	52.7	-0.642(-0.870,-0.414)
Age-standardized	DALYs	Rate	Bahamas	Both	Encephalitis	69	78.4	61	10.9	13.4	8.8	-5.795(-7.975,-3.563)
Age-standardized	DALYs	Rate	Bahrain	Both	Encephalitis	9.9	12.1	8.2	7.7	9.6	5.7	-0.419(-1.204,0.372)
Age-standardized	DALYs	Rate	Bangladesh	Both	Encephalitis	16.5	21.2	12.2	14.2	18.6	10.6	-0.325(-0.742,0.094)
Age-standardized	DALYs	Rate	Barbados	Both	Encephalitis	80.8	90.9	72.1	40	52	30.4	-2.767(-4.215,-1.297)
Age-standardized	DALYs	Rate	Belarus	Both	Encephalitis	75.2	86.3	64.1	53.1	65.1	43.6	-0.844(-1.010,-0.677)
Age-standardized	DALYs	Rate	Belgium	Both	Encephalitis	8.3	8.9	7.8	11.8	12.9	10.7	0.982(0.745,1.218)
Age-standardized	DALYs	Rate	Belize	Both	Encephalitis	118.8	136.8	105.2	11.3	13.1	9.8	-7.127(-8.036,-6.209)
Age-standardized	DALYs	Rate	Benin	Both	Encephalitis	120	173.7	81.8	83.4	116	53.7	-1.219(-1.480,-0.958)
Age-standardized	DALYs	Rate	Bermuda	Both	Encephalitis	36.9	43.2	31.4	4.4	5.4	3.5	-5.491(-8.026,-2.887)
Age-standardized	DALYs	Rate	Bhutan	Both	Encephalitis	242.7	353.8	108.9	164.8	237.7	89.5	-1.608(-1.771,-1.446)
Age-standardized	DALYs	Rate	Bolivia (Plurinational State of)	Both	Encephalitis	99.4	137	70.1	63	89.4	46.2	-1.531(-1.595,-1.467)
Age-standardized	DALYs	Rate	Bosnia and Herzegovina	Both	Encephalitis	29.8	37	23.2	12.1	18.4	8.7	-3.944(-4.427,-3.459)
Age-standardized	DALYs	Rate	Botswana	Both	Encephalitis	23.2	31.4	16.7	19.8	26.5	14.4	-0.527(-0.619,-0.435)
Age-standardized	DALYs	Rate	Brazil	Both	Encephalitis	23.4	25.5	21.4	14.2	15.7	12.8	-1.536(-1.702,-1.369)
Age-standardized	DALYs	Rate	Brunei Darussalam	Both	Encephalitis	8.4	10.6	5.6	6.1	7.6	4.5	0.424(-0.523,1.379)
Age-standardized	DALYs	Rate	Bulgaria	Both	Encephalitis	54.6	63.6	46.9	61.9	74.8	51.5	0.160(-0.263,0.586)
Age-standardized	DALYs	Rate	Burkina Faso	Both	Encephalitis	140.5	207.9	89	84.4	124.5	57.1	-1.702(-1.962,-1.442)
Age-standardized	DALYs	Rate	Burundi	Both	Encephalitis	79.6	125.3	47.2	53	81.2	30.4	-1.255(-1.414,-1.095)
Age-standardized	DALYs	Rate	Cabo Verde	Both	Encephalitis	56	75.6	40	56.5	82.9	34.5	0.140(-0.077,0.358)
Age-standardized	DALYs	Rate	Cambodia	Both	Encephalitis	76.9	124.4	49.3	70.6	97	48.6	-0.587(-0.788,-0.386)
Age-standardized	DALYs	Rate	Cameroon	Both	Encephalitis	94.1	128.8	70.5	78.6	112.8	52.7	-0.398(-0.593,-0.202)
Age-standardized	DALYs	Rate	Canada	Both	Encephalitis	4.6	4.8	4.3	8.4	9.2	7.6	2.071(1.667,2.477)
Age-standardized	DALYs	Rate	Central African Republic	Both	Encephalitis	35.5	56.7	24.3	37.6	50	26.3	0.223(0.097,0.350)
Age-standardized	DALYs	Rate	Chad	Both	Encephalitis	114	174.5	72.9	93.5	141.5	59.4	-0.462(-0.576,-0.347)
Age-standardized	DALYs	Rate	Chile	Both	Encephalitis	11.2	12.2	10.3	17.8	19.4	16.4	1.578(0.971,2.188)
Age-standardized	DALYs	Rate	China	Both	Encephalitis	98.8	121.8	69.3	29.2	38.8	23.7	-4.512(-4.850,-4.172)
Age-standardized	DALYs	Rate	Colombia	Both	Encephalitis	40.1	44.3	36.5	34.5	42.1	28	0.357(-0.364,1.083)
Age-standardized	DALYs	Rate	Comoros	Both	Encephalitis	74.9	114.2	43.5	59.8	85.8	39.7	-1.066(-1.276,-0.856)
Age-standardized	DALYs	Rate	Congo	Both	Encephalitis	30	39.7	22.5	25.5	33.9	17.9	-0.580(-0.684,-0.476)
Age-standardized	DALYs	Rate	Cook Islands	Both	Encephalitis	3.9	4.9	3	3.3	4.2	2.4	-0.703(-0.763,-0.643)
Age-standardized	DALYs	Rate	Costa Rica	Both	Encephalitis	76	81.5	70.1	27.9	31.3	24.7	-3.125(-3.995,-2.246)
Age-standardized	DALYs	Rate	Croatia	Both	Encephalitis	5.3	6.1	4.6	8.5	9.8	7.4	0.558(-0.689,1.821)
Age-standardized	DALYs	Rate	Cuba	Both	Encephalitis	75.7	81.4	70.2	23.4	26.8	20.2	-2.711(-4.159,-1.242)
Age-standardized	DALYs	Rate	Cyprus	Both	Encephalitis	3.9	5.2	2.4	2.2	3	1.2	-1.945(-2.506,-1.381)
Age-standardized	DALYs	Rate	Czechia	Both	Encephalitis	2.1	2.6	1.7	10.3	12	8.7	2.326(0.504,4.181)
Age-standardized	DALYs	Rate	Cote d'Ivoire	Both	Encephalitis	88.8	122.3	65.5	73.8	105.4	46.8	-0.417(-0.726,-0.107)
Age-standardized	DALYs	Rate	Democratic People's Republic of Korea	Both	Encephalitis	68.1	102.8	39.2	53.7	78.4	34	-0.796(-0.911,-0.680)
Age-standardized	DALYs	Rate	Democratic Republic of the Congo	Both	Encephalitis	32.3	44	23	28.2	37.9	20.9	-0.383(-0.486,-0.281)
Age-standardized	DALYs	Rate	Denmark	Both	Encephalitis	0.8	1	0.6	5.7	6.3	5.2	3.474(1.264,5.732)
Age-standardized	DALYs	Rate	Djibouti	Both	Encephalitis	58.7	82.4	37.8	51.8	75.8	33.4	-0.603(-0.837,-0.367)
Age-standardized	DALYs	Rate	Dominica	Both	Encephalitis	28.6	37.6	13.8	13.5	18.8	10	-2.948(-4.227,-1.651)
Age-standardized	DALYs	Rate	Dominican Republic	Both	Encephalitis	67.3	84.1	47	52.2	73.6	33.1	-0.845(-1.396,-0.291)
Age-standardized	DALYs	Rate	Ecuador	Both	Encephalitis	52.9	57.3	48.7	33.5	39.8	28	-2.111(-2.550,-1.670)
Age-standardized	DALYs	Rate	Egypt	Both	Encephalitis	94.1	117.4	72	49.5	64.4	39.3	-2.306(-2.472,-2.139)
Age-standardized	DALYs	Rate	El Salvador	Both	Encephalitis	65.4	78.9	45.6	19.8	27.8	15.2	-3.642(-4.399,-2.880)
Age-standardized	DALYs	Rate	Equatorial Guinea	Both	Encephalitis	32.4	46.7	23.1	22.1	34.2	12.5	-1.569(-1.701,-1.436)
Age-standardized	DALYs	Rate	Eritrea	Both	Encephalitis	97.7	152.7	47.1	75.3	108.2	47.3	-0.989(-1.098,-0.880)
Age-standardized	DALYs	Rate	Estonia	Both	Encephalitis	20.5	23.5	18	10.3	11.9	8.9	-2.463(-3.096,-1.825)
Age-standardized	DALYs	Rate	Eswatini	Both	Encephalitis	23.9	30.6	17.9	22	30.8	15.2	-0.292(-0.356,-0.228)
Age-standardized	DALYs	Rate	Ethiopia	Both	Encephalitis	79.4	111.5	41.5	51.9	70.9	33.8	-1.601(-1.727,-1.476)
Age-standardized	DALYs	Rate	Fiji	Both	Encephalitis	23.7	33.1	17.3	20.8	29.7	14.4	-0.440(-0.700,-0.179)
Age-standardized	DALYs	Rate	Finland	Both	Encephalitis	5.5	6.2	5	7	7.7	6.4	0.617(-0.350,1.594)
Age-standardized	DALYs	Rate	France	Both	Encephalitis	9.8	10.3	9.3	7.7	8.2	7.2	-0.238(-0.525,0.048)
Age-standardized	DALYs	Rate	Gabon	Both	Encephalitis	27.5	36.3	19.2	23.1	33.2	14.7	-0.548(-0.644,-0.451)
Age-standardized	DALYs	Rate	Gambia	Both	Encephalitis	90.7	121.3	66.3	80	113.5	57.3	-0.627(-0.898,-0.355)
Age-standardized	DALYs	Rate	Georgia	Both	Encephalitis	33.3	43.8	25.5	28.2	37.5	22.2	-0.429(-1.432,0.585)
Age-standardized	DALYs	Rate	Germany	Both	Encephalitis	7.1	7.5	6.6	11.9	12.8	11.2	1.553(1.223,1.884)
Age-standardized	DALYs	Rate	Ghana	Both	Encephalitis	87.6	115.2	67.4	77.8	115.3	51.7	-0.336(-0.466,-0.206)
Age-standardized	DALYs	Rate	Greece	Both	Encephalitis	5.7	6.5	5	17.2	18.7	15.7	4.754(3.353,6.174)
Age-standardized	DALYs	Rate	Greenland	Both	Encephalitis	9.1	12.1	7	14.4	19.5	7.1	2.176(1.526,2.830)
Age-standardized	DALYs	Rate	Grenada	Both	Encephalitis	30.9	34.9	27.2	14.3	16.7	12.3	-3.280(-4.708,-1.830)
Age-standardized	DALYs	Rate	Guam	Both	Encephalitis	2.6	3.4	2	2.4	3.1	1.8	0.035(-0.059,0.129)
Age-standardized	DALYs	Rate	Guatemala	Both	Encephalitis	52.1	57.3	48	37.6	45.4	31.2	-1.338(-1.973,-0.700)
Age-standardized	DALYs	Rate	Guinea	Both	Encephalitis	111.5	174.2	73	82.9	122.3	57.5	-0.832(-0.913,-0.751)
Age-standardized	DALYs	Rate	Guinea-Bissau	Both	Encephalitis	119.1	188.8	82.3	90.4	122.1	65.5	-0.610(-0.755,-0.466)
Age-standardized	DALYs	Rate	Guyana	Both	Encephalitis	89.5	104.9	75.5	56.1	70.2	43.3	-1.255(-1.954,-0.551)
Age-standardized	DALYs	Rate	Haiti	Both	Encephalitis	41.5	77	26.2	23.8	37.5	15.9	-1.841(-2.165,-1.517)
Age-standardized	DALYs	Rate	Honduras	Both	Encephalitis	55.6	69.8	41.5	45.5	67.3	28.2	-0.598(-0.814,-0.382)
Age-standardized	DALYs	Rate	Hungary	Both	Encephalitis	25.2	27.6	23	7.1	8.5	6.1	-4.766(-5.327,-4.201)
Age-standardized	DALYs	Rate	Iceland	Both	Encephalitis	1.3	1.4	1.1	0.5	0.5	0.4	-1.210(-3.853,1.506)
Age-standardized	DALYs	Rate	India	Both	Encephalitis	310.5	363.3	248.6	169.8	216.9	136.3	-2.280(-2.543,-2.016)
Age-standardized	DALYs	Rate	Indonesia	Both	Encephalitis	85.9	122.8	49.9	80.6	110.2	44.6	-0.115(-0.195,-0.036)
Age-standardized	DALYs	Rate	Iran (Islamic Republic of)	Both	Encephalitis	19.8	25.9	16.1	13.3	15.3	10.9	-0.420(-0.736,-0.103)
Age-standardized	DALYs	Rate	Iraq	Both	Encephalitis	127.1	175.5	90.5	82.4	109.9	60.7	-1.293(-1.395,-1.191)
Age-standardized	DALYs	Rate	Ireland	Both	Encephalitis	3.6	3.9	3.2	4.4	4.8	4	0.918(0.240,1.601)
Age-standardized	DALYs	Rate	Israel	Both	Encephalitis	7	7.5	6.5	7.5	8.2	6.9	-0.150(-1.058,0.765)
Age-standardized	DALYs	Rate	Italy	Both	Encephalitis	16.6	17.3	16	17.5	18.6	16.4	0.168(-0.040,0.376)
Age-standardized	DALYs	Rate	Jamaica	Both	Encephalitis	76.3	86.5	68	31.4	40.3	24.2	-2.033(-2.622,-1.439)
Age-standardized	DALYs	Rate	Japan	Both	Encephalitis	13.2	14	12.4	8.9	9.7	8.2	-2.157(-2.598,-1.714)
Age-standardized	DALYs	Rate	Jordan	Both	Encephalitis	25.7	32.3	18.7	8.1	10	6.7	-4.735(-5.441,-4.024)
Age-standardized	DALYs	Rate	Kazakhstan	Both	Encephalitis	91.9	113.7	73.2	59.9	79.9	43.8	-2.820(-3.697,-1.935)
Age-standardized	DALYs	Rate	Kenya	Both	Encephalitis	45.9	55.4	36.1	53.8	66.6	43	0.976(0.744,1.208)
Age-standardized	DALYs	Rate	Kiribati	Both	Encephalitis	11.2	14.3	8.4	11.9	15.7	8.6	0.588(0.257,0.921)
Age-standardized	DALYs	Rate	Kuwait	Both	Encephalitis	11	12.1	10.1	5.7	6.7	4.8	-1.042(-2.241,0.172)
Age-standardized	DALYs	Rate	Kyrgyzstan	Both	Encephalitis	135.1	171.8	105.2	55.9	69	45	-3.646(-4.160,-3.130)
Age-standardized	DALYs	Rate	Lao People's Democratic Republic	Both	Encephalitis	68.1	138.1	46	70.2	97.3	48.9	0.129(-0.064,0.323)
Age-standardized	DALYs	Rate	Latvia	Both	Encephalitis	83.3	94.5	74	29	33.1	24.7	-4.546(-4.934,-4.157)
Age-standardized	DALYs	Rate	Lebanon	Both	Encephalitis	14	18.7	10.7	10.9	13.6	8.8	-0.688(-0.816,-0.559)
Age-standardized	DALYs	Rate	Lesotho	Both	Encephalitis	21.8	29.3	15.1	19.5	26.3	14.6	-0.273(-0.378,-0.168)
Age-standardized	DALYs	Rate	Liberia	Both	Encephalitis	127.3	177.6	88.3	73.4	110.1	46.8	-1.704(-2.105,-1.303)
Age-standardized	DALYs	Rate	Libya	Both	Encephalitis	13.5	21	9.9	12.7	18.5	9.5	-0.057(-0.106,-0.008)
Age-standardized	DALYs	Rate	Lithuania	Both	Encephalitis	38	42.5	33.8	26	29.4	22.5	-2.067(-2.542,-1.589)
Age-standardized	DALYs	Rate	Luxembourg	Both	Encephalitis	1.8	2.1	1.5	1.7	2	1.5	-0.109(-1.532,1.335)
Age-standardized	DALYs	Rate	Madagascar	Both	Encephalitis	70	94.9	46.9	55.6	81.3	36.3	-0.820(-0.915,-0.724)
Age-standardized	DALYs	Rate	Malawi	Both	Encephalitis	61.5	78.8	40.8	49.3	70.2	32.4	-0.730(-0.827,-0.633)
Age-standardized	DALYs	Rate	Malaysia	Both	Encephalitis	75.6	93.1	58.9	63.9	75.8	50.3	-0.453(-0.551,-0.356)
Age-standardized	DALYs	Rate	Maldives	Both	Encephalitis	40.5	59.4	26.9	20.8	27.2	16.3	-2.186(-2.424,-1.947)
Age-standardized	DALYs	Rate	Mali	Both	Encephalitis	130.7	198.3	85.7	80.2	113.4	57.9	-1.474(-1.699,-1.249)
Age-standardized	DALYs	Rate	Malta	Both	Encephalitis	4.8	5.4	4.3	0.9	1.1	0.8	-6.081(-7.891,-4.235)
Age-standardized	DALYs	Rate	Marshall Islands	Both	Encephalitis	5.5	6.8	4.2	5	6.3	3.8	-0.312(-0.372,-0.253)
Age-standardized	DALYs	Rate	Mauritania	Both	Encephalitis	103.2	145.8	69.4	74.3	100.5	51.1	-1.101(-1.206,-0.996)
Age-standardized	DALYs	Rate	Mauritius	Both	Encephalitis	28.4	30.9	26	50.4	56.2	44.6	2.742(2.220,3.266)
Age-standardized	DALYs	Rate	Mexico	Both	Encephalitis	52	57	48.2	47.4	54.6	41.6	-0.660(-1.303,-0.014)
Age-standardized	DALYs	Rate	Micronesia (Federated States of)	Both	Encephalitis	6	7.5	4.6	5.3	6.8	4.1	-0.330(-0.363,-0.297)
Age-standardized	DALYs	Rate	Monaco	Both	Encephalitis	2.9	3.8	2	1.8	2.4	1.3	-1.891(-2.365,-1.415)
Age-standardized	DALYs	Rate	Mongolia	Both	Encephalitis	129.2	208.7	77.8	78.2	116.5	51.6	-1.850(-1.980,-1.719)
Age-standardized	DALYs	Rate	Montenegro	Both	Encephalitis	5.3	7.3	3.5	2.8	3.6	2.1	-2.280(-2.513,-2.046)
Age-standardized	DALYs	Rate	Morocco	Both	Encephalitis	20.7	34.3	13.2	17.2	24.2	12	-0.358(-0.490,-0.226)
Age-standardized	DALYs	Rate	Mozambique	Both	Encephalitis	62.7	87.7	46.8	53.3	76.1	35.9	-0.448(-0.546,-0.351)
Age-standardized	DALYs	Rate	Myanmar	Both	Encephalitis	68.4	110.9	41.2	60.8	90.7	36.5	-0.402(-0.596,-0.209)
Age-standardized	DALYs	Rate	Namibia	Both	Encephalitis	22.3	28.6	16.8	21.1	31.1	14.7	-0.238(-0.301,-0.174)
Age-standardized	DALYs	Rate	Nauru	Both	Encephalitis	4.1	5.5	3.1	5.4	7.1	4.1	1.137(0.670,1.607)
Age-standardized	DALYs	Rate	Nepal	Both	Encephalitis	277.4	398.9	161.3	150.2	201.5	89.5	-2.046(-2.142,-1.950)
Age-standardized	DALYs	Rate	Netherlands	Both	Encephalitis	8	8.6	7.6	6	6.5	5.5	-1.798(-2.402,-1.192)
Age-standardized	DALYs	Rate	New Zealand	Both	Encephalitis	2.4	2.7	2	9.2	9.9	8.4	5.766(4.531,7.016)
Age-standardized	DALYs	Rate	Nicaragua	Both	Encephalitis	58.8	71.8	42.5	40.3	52.7	30	-1.094(-1.254,-0.934)
Age-standardized	DALYs	Rate	Niger	Both	Encephalitis	183.4	304.8	91	97.2	146.7	58	-2.137(-2.438,-1.835)
Age-standardized	DALYs	Rate	Nigeria	Both	Encephalitis	119.8	165.1	74.4	89.8	121.9	59	-0.759(-0.987,-0.531)
Age-standardized	DALYs	Rate	Niue	Both	Encephalitis	5	6.3	3.8	4.4	5.6	3.4	-0.566(-0.637,-0.495)
Age-standardized	DALYs	Rate	North Macedonia	Both	Encephalitis	34.6	44.6	24.2	20.1	27.1	13.8	-1.744(-2.067,-1.420)
Age-standardized	DALYs	Rate	Northern Mariana Islands	Both	Encephalitis	3.5	4.5	2.5	3.3	4.3	2.3	-0.482(-0.666,-0.297)
Age-standardized	DALYs	Rate	Norway	Both	Encephalitis	4.1	4.4	3.9	4.2	4.5	3.9	0.970(0.457,1.485)
Age-standardized	DALYs	Rate	Oman	Both	Encephalitis	165.2	222.8	111.7	44	72.6	29.2	-5.135(-5.803,-4.461)
Age-standardized	DALYs	Rate	Pakistan	Both	Encephalitis	193.7	243.2	115.5	190.6	268.2	100.9	0.086(0.006,0.166)
Age-standardized	DALYs	Rate	Palau	Both	Encephalitis	3.4	4.5	2.5	3.2	4.2	2.4	-0.219(-0.303,-0.135)
Age-standardized	DALYs	Rate	Palestine	Both	Encephalitis	11.4	15.4	8.8	16.5	20.8	12.4	2.546(1.921,3.174)
Age-standardized	DALYs	Rate	Panama	Both	Encephalitis	59.3	68	51.5	20	24	16.5	-3.044(-3.660,-2.425)
Age-standardized	DALYs	Rate	Papua New Guinea	Both	Encephalitis	30.7	44	21	34.3	54.1	21.6	0.393(0.260,0.526)
Age-standardized	DALYs	Rate	Paraguay	Both	Encephalitis	33	41	24.4	25.5	34.4	18.8	-0.779(-0.987,-0.571)
Age-standardized	DALYs	Rate	Peru	Both	Encephalitis	113.9	142	88.2	62.4	85.1	44.1	-2.273(-2.764,-1.780)
Age-standardized	DALYs	Rate	Philippines	Both	Encephalitis	90.3	117	68.9	82.8	100.4	54.6	-0.148(-0.299,0.004)
Age-standardized	DALYs	Rate	Poland	Both	Encephalitis	47	50.3	44.1	13.7	15.1	12.3	-3.693(-4.180,-3.204)
Age-standardized	DALYs	Rate	Portugal	Both	Encephalitis	16.7	17.9	15.5	16	17.4	14.4	-0.392(-1.608,0.840)
Age-standardized	DALYs	Rate	Puerto Rico	Both	Encephalitis	65.7	70.5	60.9	12	13.8	10.4	-3.388(-4.720,-2.038)
Age-standardized	DALYs	Rate	Qatar	Both	Encephalitis	17.7	23.3	10.8	7.9	10.2	5.1	-2.843(-3.111,-2.574)
Age-standardized	DALYs	Rate	Republic of Korea	Both	Encephalitis	11.7	20.2	9.2	14.2	17.4	11.2	0.408(-0.162,0.982)
Age-standardized	DALYs	Rate	Republic of Moldova	Both	Encephalitis	32.6	37	28.7	35.7	42.1	29.6	-0.255(-0.932,0.426)
Age-standardized	DALYs	Rate	Romania	Both	Encephalitis	109.5	119	101.3	37.8	43.6	32.5	-4.717(-5.423,-4.005)
Age-standardized	DALYs	Rate	Russian Federation	Both	Encephalitis	63.3	65	61.5	40.1	42.7	37.9	-2.093(-2.353,-1.832)
Age-standardized	DALYs	Rate	Rwanda	Both	Encephalitis	66.9	90.9	46.2	49.9	70.1	33.2	-1.495(-1.738,-1.252)
Age-standardized	DALYs	Rate	Saint Kitts and Nevis	Both	Encephalitis	18.2	20.4	16.1	31.9	40.1	24.4	-0.032(-2.577,2.580)
Age-standardized	DALYs	Rate	Saint Lucia	Both	Encephalitis	75.4	85.3	66.4	11.9	14.5	9.8	-5.835(-6.985,-4.671)
Age-standardized	DALYs	Rate	Saint Vincent and the Grenadines	Both	Encephalitis	25.4	29.7	22.2	8.1	9.6	6.8	-5.014(-6.372,-3.636)
Age-standardized	DALYs	Rate	Samoa	Both	Encephalitis	5.6	7.1	4.2	4.9	6.2	3.7	-0.474(-0.532,-0.415)
Age-standardized	DALYs	Rate	San Marino	Both	Encephalitis	14.7	20.8	11	11.7	17.1	7.4	0.445(0.099,0.792)
Age-standardized	DALYs	Rate	Sao Tome and Principe	Both	Encephalitis	160.6	213.4	114.7	121.3	177.4	79.6	-0.676(-1.101,-0.250)
Age-standardized	DALYs	Rate	Saudi Arabia	Both	Encephalitis	65.9	89.8	47.8	34.4	45.9	25.8	-2.720(-3.024,-2.415)
Age-standardized	DALYs	Rate	Senegal	Both	Encephalitis	116.1	164.4	81.7	85.6	120.4	59.1	-0.716(-0.956,-0.477)
Age-standardized	DALYs	Rate	Serbia	Both	Encephalitis	37.5	50.5	30	27.5	33.9	21.5	-1.223(-1.494,-0.951)
Age-standardized	DALYs	Rate	Seychelles	Both	Encephalitis	11.4	21.3	8.7	23.7	29.5	13.9	2.223(1.192,3.264)
Age-standardized	DALYs	Rate	Sierra Leone	Both	Encephalitis	120.8	176.1	80.3	79	116.6	50.4	-1.557(-1.809,-1.304)
Age-standardized	DALYs	Rate	Singapore	Both	Encephalitis	21.2	22.9	19.5	16.4	18.4	14.6	-1.075(-1.941,-0.202)
Age-standardized	DALYs	Rate	Slovakia	Both	Encephalitis	10.4	12.7	8.6	9.6	12.4	7.4	-0.308(-0.515,-0.101)
Age-standardized	DALYs	Rate	Slovenia	Both	Encephalitis	7.6	8.4	6.9	5.5	6.4	4.7	-1.026(-2.086,0.044)
Age-standardized	DALYs	Rate	Solomon Islands	Both	Encephalitis	5.5	7	4.2	5.5	6.9	4.2	0.069(-0.004,0.143)
Age-standardized	DALYs	Rate	Somalia	Both	Encephalitis	71.5	98.8	49.7	64	99.4	39.5	-0.234(-0.398,-0.071)
Age-standardized	DALYs	Rate	South Africa	Both	Encephalitis	25	28.8	18.8	17	21.4	14.2	-1.398(-1.768,-1.027)
Age-standardized	DALYs	Rate	South Sudan	Both	Encephalitis	69.6	99	46.3	69.1	99	45.6	-0.095(-0.430,0.242)
Age-standardized	DALYs	Rate	Spain	Both	Encephalitis	14.2	15.1	13.3	12.4	13.5	11.5	-0.285(-0.542,-0.027)
Age-standardized	DALYs	Rate	Sri Lanka	Both	Encephalitis	79.6	112.9	66.9	51.5	77	36.6	-1.635(-1.911,-1.359)
Age-standardized	DALYs	Rate	Sudan	Both	Encephalitis	29.5	53.5	15.3	23.8	36.2	15.9	-0.394(-0.528,-0.259)
Age-standardized	DALYs	Rate	Suriname	Both	Encephalitis	59.1	70.2	46.3	50.2	67.1	37.7	-0.576(-0.844,-0.307)
Age-standardized	DALYs	Rate	Sweden	Both	Encephalitis	4.3	5	3.7	7.4	8.3	6.6	2.216(1.913,2.521)
Age-standardized	DALYs	Rate	Switzerland	Both	Encephalitis	1.4	1.8	1.1	8.4	9.2	7.6	4.064(2.029,6.139)
Age-standardized	DALYs	Rate	Syrian Arab Republic	Both	Encephalitis	35.7	45.7	27.7	33.5	43.4	24	-0.586(-1.089,-0.080)
Age-standardized	DALYs	Rate	Taiwan (Province of China)	Both	Encephalitis	19.1	20.7	17.6	17.3	19.2	15.5	0.457(0.106,0.809)
Age-standardized	DALYs	Rate	Tajikistan	Both	Encephalitis	5.9	7.9	4.2	5.9	7.7	4.2	-0.412(-0.749,-0.075)
Age-standardized	DALYs	Rate	Thailand	Both	Encephalitis	87.8	115.7	57.5	50.9	65.8	40.1	-2.344(-2.686,-2.001)
Age-standardized	DALYs	Rate	Timor-Leste	Both	Encephalitis	87.7	138.2	44.8	87.2	123.8	56.2	-0.253(-0.428,-0.077)
Age-standardized	DALYs	Rate	Togo	Both	Encephalitis	95.8	129	72.8	73.1	107	48	-0.952(-1.107,-0.797)
Age-standardized	DALYs	Rate	Tokelau	Both	Encephalitis	5.6	7.2	4.3	5.3	6.7	4.2	-0.415(-0.515,-0.315)
Age-standardized	DALYs	Rate	Tonga	Both	Encephalitis	5.5	7.2	3.9	4.9	6.3	3.5	-0.293(-0.337,-0.248)
Age-standardized	DALYs	Rate	Trinidad and Tobago	Both	Encephalitis	111.5	125.5	99.5	18.4	23.4	14.6	-6.582(-7.064,-6.098)
Age-standardized	DALYs	Rate	Tunisia	Both	Encephalitis	18.6	28	14.2	14.1	19.4	10.6	-0.737(-0.811,-0.664)
Age-standardized	DALYs	Rate	Turkey	Both	Encephalitis	32.6	44.8	24.6	16.7	20.5	13.3	-2.253(-2.526,-1.980)
Age-standardized	DALYs	Rate	Turkmenistan	Both	Encephalitis	35.1	43.6	28.1	103	135.8	77.1	3.788(2.597,4.992)
Age-standardized	DALYs	Rate	Tuvalu	Both	Encephalitis	5.7	7.4	4.4	5.1	6.5	3.9	-0.222(-0.326,-0.117)
Age-standardized	DALYs	Rate	Uganda	Both	Encephalitis	49.3	68.6	32.5	45.9	66.1	29	-0.295(-0.378,-0.211)
Age-standardized	DALYs	Rate	Ukraine	Both	Encephalitis	73.7	83.2	66	60.4	73.1	48.1	-0.636(-0.798,-0.474)
Age-standardized	DALYs	Rate	United Arab Emirates	Both	Encephalitis	10	14.3	5.9	6	7.9	3.7	-1.729(-2.058,-1.398)
Age-standardized	DALYs	Rate	United Kingdom	Both	Encephalitis	4.7	5.2	4.3	15.1	15.7	14.4	4.311(3.999,4.623)
Age-standardized	DALYs	Rate	United Republic of Tanzania	Both	Encephalitis	73.4	100.8	48	56.9	80.8	36.7	-0.704(-0.782,-0.625)
Age-standardized	DALYs	Rate	United States of America	Both	Encephalitis	5.9	6.1	5.8	8	8.4	7.6	1.458(0.910,2.010)
Age-standardized	DALYs	Rate	United States Virgin Islands	Both	Encephalitis	2.6	3.5	1.9	2.5	3.3	1.8	-0.156(-0.300,-0.013)
Age-standardized	DALYs	Rate	Uruguay	Both	Encephalitis	37	39.8	34.2	37	41.1	33.4	-0.582(-1.265,0.105)
Age-standardized	DALYs	Rate	Uzbekistan	Both	Encephalitis	134.8	172.1	108	85.6	105.8	68.1	-1.433(-1.863,-1.001)
Age-standardized	DALYs	Rate	Vanuatu	Both	Encephalitis	5.3	6.8	4	5.2	6.6	3.9	-0.094(-0.152,-0.036)
Age-standardized	DALYs	Rate	Venezuela (Bolivarian Republic of)	Both	Encephalitis	74.6	78.4	70.3	36.8	45.5	29.1	-1.909(-2.832,-0.978)
Age-standardized	DALYs	Rate	Viet Nam	Both	Encephalitis	145.9	206.8	101.2	108.4	151.9	73.4	-1.027(-1.096,-0.957)
Age-standardized	DALYs	Rate	Yemen	Both	Encephalitis	26.3	42.5	12.9	26.1	37.9	16.1	0.087(-0.054,0.227)
Age-standardized	DALYs	Rate	Zambia	Both	Encephalitis	58.4	78.8	41.4	71.3	106.8	38.1	0.974(0.768,1.181)
Age-standardized	DALYs	Rate	Zimbabwe	Both	Encephalitis	29.3	37.6	17.4	50.7	70.1	26.2	2.038(1.528,2.551)

ASDR age-standardized DALYs rate, *EAPC* estimated annual percentage change, *CI* confidence interval, *UI* uncertainty interval
