# Supplementary material for: Socioeconomic drivers of encephalitis burden in the post-COVID era: a 204-country analysis from global burden of disease study 2021
Source: Front Public Health. 2025 Sep 18;13:1651734. doi: 10.3389/fpubh.2025.1651734 (PMC12488571; doi:10.3389/fpubh.2025.1651734)
Supplement: SUPPLEMENTARY FIGURE S5 — (A) Age-standardized incidence rates of encephalitis for 21 regions by SDI from 1990–2021. The expected values based on the SDI and disease rates at all of the locations are shown as black lines. (B) Age-standardized incidence rates for encephalitis in 204 countries and territories by SDI in 2021. Expected values based on the sociodemographic index and disease rate at all of the locations are shown as black lines. SDI, sociodemographic index. [file Data_Sheet_5.PDF]

Table S5: The ASMR of encephalitis in 204 countries and territories in 1990 and 2021 and EAPC of ASMR for encephalitis in 204 countries and territories from 1990 to 2021

age_name	measure_name	metric_name	location_name	sex_name	cause_name	1990 ASMR	1990 ASMR 95%UI upper	1990 ASMR 95%UI lower	2021 ASMR	2021 ASMR 95%UI upper	2021 ASMR 95%UI lower	EAPC (95%CI)
Age-standardized	Deaths	Rate	Afghanistan	Both	Encephalitis	0.55	0.99	0.28	0.55	0.8	0.35	0.343(0.203,0.483)
Age-standardized	Deaths	Rate	Albania	Both	Encephalitis	1.53	1.94	1.2	0.67	1.05	0.41	-3.823(-4.358,-3.286)
Age-standardized	Deaths	Rate	Algeria	Both	Encephalitis	0.31	0.46	0.23	0.28	0.37	0.2	-0.095(-0.199,0.010)
Age-standardized	Deaths	Rate	American Samoa	Both	Encephalitis	0.11	0.16	0.05	0.06	0.09	0.01	-2.825(-3.439,-2.206)
Age-standardized	Deaths	Rate	Andorra	Both	Encephalitis	0.22	0.32	0.16	0.14	0.19	0.1	-1.152(-1.306,-0.999)
Age-standardized	Deaths	Rate	Angola	Both	Encephalitis	0.66	1	0.42	0.54	0.72	0.37	-0.672(-0.737,-0.608)
Age-standardized	Deaths	Rate	Antigua and Barbuda	Both	Encephalitis	0.18	0.21	0.16	0.1	0.11	0.09	-2.165(-4.329,0.049)
Age-standardized	Deaths	Rate	Argentina	Both	Encephalitis	0.46	0.49	0.43	0.41	0.45	0.38	-0.184(-0.773,0.409)
Age-standardized	Deaths	Rate	Armenia	Both	Encephalitis	0.37	0.52	0.27	0.45	0.61	0.31	0.851(0.516,1.186)
Age-standardized	Deaths	Rate	Australia	Both	Encephalitis	0.08	0.08	0.07	0.17	0.19	0.16	2.955(2.490,3.422)
Age-standardized	Deaths	Rate	Austria	Both	Encephalitis	0.23	0.25	0.22	0.21	0.23	0.2	-0.123(-0.746,0.505)
Age-standardized	Deaths	Rate	Azerbaijan	Both	Encephalitis	1.47	2.36	0.88	1.35	2.49	0.7	-0.615(-0.833,-0.397)
Age-standardized	Deaths	Rate	Bahamas	Both	Encephalitis	1.24	1.39	1.11	0.17	0.22	0.13	-6.161(-8.832,-3.413)
Age-standardized	Deaths	Rate	Bahrain	Both	Encephalitis	0.14	0.19	0.12	0.11	0.14	0.08	-0.415(-1.459,0.640)
Age-standardized	Deaths	Rate	Bangladesh	Both	Encephalitis	0.33	0.46	0.2	0.27	0.4	0.17	-0.753(-1.007,-0.498)
Age-standardized	Deaths	Rate	Barbados	Both	Encephalitis	1.35	1.48	1.23	0.67	0.86	0.52	-2.817(-4.330,-1.281)
Age-standardized	Deaths	Rate	Belarus	Both	Encephalitis	1.1	1.24	0.95	0.92	1.12	0.74	-0.328(-0.481,-0.174)
Age-standardized	Deaths	Rate	Belgium	Both	Encephalitis	0.17	0.18	0.16	0.28	0.31	0.26	1.648(1.387,1.909)
Age-standardized	Deaths	Rate	Belize	Both	Encephalitis	1.63	1.85	1.46	0.13	0.15	0.11	-7.744(-8.798,-6.678)
Age-standardized	Deaths	Rate	Benin	Both	Encephalitis	1.9	2.63	1.34	1.4	1.91	0.94	-1.078(-1.271,-0.885)
Age-standardized	Deaths	Rate	Bermuda	Both	Encephalitis	0.65	0.73	0.57	0.06	0.07	0.05	-6.209(-9.915,-2.350)
Age-standardized	Deaths	Rate	Bhutan	Both	Encephalitis	6.45	9.56	3.25	4.08	5.63	2.18	-1.741(-1.863,-1.620)
Age-standardized	Deaths	Rate	Bolivia (Plurinational State of)	Both	Encephalitis	1.38	1.89	0.99	0.95	1.35	0.69	-1.234(-1.294,-1.174)
Age-standardized	Deaths	Rate	Bosnia and Herzegovina	Both	Encephalitis	0.59	0.75	0.45	0.25	0.36	0.18	-3.841(-4.326,-3.353)
Age-standardized	Deaths	Rate	Botswana	Both	Encephalitis	0.42	0.58	0.3	0.37	0.49	0.26	-0.464(-0.518,-0.410)
Age-standardized	Deaths	Rate	Brazil	Both	Encephalitis	0.32	0.34	0.3	0.26	0.28	0.24	-0.526(-0.735,-0.316)
Age-standardized	Deaths	Rate	Brunei Darussalam	Both	Encephalitis	0.15	0.2	0.1	0.13	0.18	0.08	1.003(-0.025,2.041)
Age-standardized	Deaths	Rate	Bulgaria	Both	Encephalitis	0.78	0.88	0.68	1.1	1.32	0.91	0.912(0.450,1.376)
Age-standardized	Deaths	Rate	Burkina Faso	Both	Encephalitis	2.24	3.14	1.51	1.43	2.02	0.97	-1.581(-1.808,-1.353)
Age-standardized	Deaths	Rate	Burundi	Both	Encephalitis	1.15	1.69	0.71	0.84	1.18	0.51	-1.077(-1.192,-0.961)
Age-standardized	Deaths	Rate	Cabo Verde	Both	Encephalitis	0.85	1.14	0.59	0.89	1.27	0.52	0.198(0.006,0.390)
Age-standardized	Deaths	Rate	Cambodia	Both	Encephalitis	1.09	1.76	0.71	1.06	1.44	0.75	-0.371(-0.568,-0.173)
Age-standardized	Deaths	Rate	Cameroon	Both	Encephalitis	1.65	2.12	1.27	1.38	1.93	0.91	-0.517(-0.664,-0.371)
Age-standardized	Deaths	Rate	Canada	Both	Encephalitis	0.1	0.11	0.1	0.2	0.22	0.18	2.245(1.823,2.669)
Age-standardized	Deaths	Rate	Central African Republic	Both	Encephalitis	0.62	0.9	0.42	0.65	0.88	0.44	0.151(0.061,0.240)
Age-standardized	Deaths	Rate	Chad	Both	Encephalitis	1.85	2.62	1.23	1.6	2.29	1.03	-0.371(-0.419,-0.322)
Age-standardized	Deaths	Rate	Chile	Both	Encephalitis	0.17	0.18	0.16	0.32	0.34	0.3	2.303(1.603,3.008)
Age-standardized	Deaths	Rate	China	Both	Encephalitis	1.11	1.36	0.77	0.33	0.44	0.26	-4.580(-4.978,-4.180)
Age-standardized	Deaths	Rate	Colombia	Both	Encephalitis	0.57	0.61	0.52	0.56	0.69	0.45	0.834(0.037,1.638)
Age-standardized	Deaths	Rate	Comoros	Both	Encephalitis	1.11	1.62	0.66	0.91	1.25	0.62	-0.975(-1.182,-0.768)
Age-standardized	Deaths	Rate	Congo	Both	Encephalitis	0.54	0.69	0.41	0.47	0.62	0.34	-0.498(-0.593,-0.404)
Age-standardized	Deaths	Rate	Cook Islands	Both	Encephalitis	0.06	0.08	0.04	0.05	0.08	0.03	-0.902(-1.035,-0.768)
Age-standardized	Deaths	Rate	Costa Rica	Both	Encephalitis	1.15	1.22	1.08	0.42	0.47	0.37	-3.129(-4.102,-2.145)
Age-standardized	Deaths	Rate	Croatia	Both	Encephalitis	0.08	0.09	0.07	0.19	0.23	0.17	1.603(-0.224,3.464)
Age-standardized	Deaths	Rate	Cuba	Both	Encephalitis	1.06	1.12	1	0.33	0.38	0.28	-2.505(-4.232,-0.747)
Age-standardized	Deaths	Rate	Cyprus	Both	Encephalitis	0.17	0.24	0.09	0.08	0.11	0.03	-2.951(-3.513,-2.386)
Age-standardized	Deaths	Rate	Czechia	Both	Encephalitis	0.01	0.01	0.01	0.22	0.26	0.18	4.683(1.933,7.507)
Age-standardized	Deaths	Rate	Cote d'Ivoire	Both	Encephalitis	1.51	2.03	1.12	1.29	1.85	0.82	-0.392(-0.604,-0.180)
Age-standardized	Deaths	Rate	Democratic People's Republic of Korea	Both	Encephalitis	0.78	1.18	0.44	0.61	0.92	0.38	-0.845(-0.965,-0.726)
Age-standardized	Deaths	Rate	Democratic Republic of the Congo	Both	Encephalitis	0.56	0.74	0.4	0.51	0.71	0.36	-0.255(-0.330,-0.180)
Age-standardized	Deaths	Rate	Denmark	Both	Encephalitis	0.01	0.01	0	0.14	0.15	0.13	6.246(2.919,9.682)
Age-standardized	Deaths	Rate	Djibouti	Both	Encephalitis	0.91	1.24	0.59	0.83	1.19	0.54	-0.476(-0.686,-0.265)
Age-standardized	Deaths	Rate	Dominica	Both	Encephalitis	0.45	0.59	0.17	0.18	0.25	0.12	-3.722(-5.564,-1.845)
Age-standardized	Deaths	Rate	Dominican Republic	Both	Encephalitis	0.95	1.14	0.7	0.75	1.01	0.5	-0.763(-1.236,-0.289)
Age-standardized	Deaths	Rate	Ecuador	Both	Encephalitis	0.8	0.85	0.75	0.61	0.74	0.5	-1.605(-2.030,-1.178)
Age-standardized	Deaths	Rate	Egypt	Both	Encephalitis	1.55	1.88	1.29	1.13	1.39	0.9	-0.890(-0.995,-0.786)
Age-standardized	Deaths	Rate	El Salvador	Both	Encephalitis	0.92	1.1	0.61	0.28	0.39	0.21	-3.667(-4.511,-2.816)
Age-standardized	Deaths	Rate	Equatorial Guinea	Both	Encephalitis	0.58	0.79	0.41	0.43	0.67	0.24	-1.268(-1.398,-1.137)
Age-standardized	Deaths	Rate	Eritrea	Both	Encephalitis	1.37	2.08	0.73	1.17	1.68	0.74	-0.632(-0.721,-0.543)
Age-standardized	Deaths	Rate	Estonia	Both	Encephalitis	0.35	0.4	0.31	0.23	0.27	0.19	-1.656(-2.323,-0.984)
Age-standardized	Deaths	Rate	Eswatini	Both	Encephalitis	0.44	0.59	0.32	0.39	0.55	0.26	-0.368(-0.497,-0.240)
Age-standardized	Deaths	Rate	Ethiopia	Both	Encephalitis	1.21	1.64	0.7	0.85	1.16	0.55	-1.358(-1.460,-1.257)
Age-standardized	Deaths	Rate	Fiji	Both	Encephalitis	0.49	1.16	0.29	0.41	0.89	0.24	-0.596(-0.875,-0.315)
Age-standardized	Deaths	Rate	Finland	Both	Encephalitis	0.09	0.1	0.09	0.15	0.16	0.13	1.264(0.079,2.463)
Age-standardized	Deaths	Rate	France	Both	Encephalitis	0.23	0.24	0.21	0.2	0.22	0.18	0.220(-0.056,0.497)
Age-standardized	Deaths	Rate	Gabon	Both	Encephalitis	0.54	0.7	0.39	0.44	0.62	0.29	-0.703(-0.795,-0.611)
Age-standardized	Deaths	Rate	Gambia	Both	Encephalitis	1.55	2.01	1.15	1.42	1.99	1.02	-0.506(-0.734,-0.277)
Age-standardized	Deaths	Rate	Georgia	Both	Encephalitis	0.49	0.64	0.37	0.55	0.71	0.43	0.686(-0.506,1.893)
Age-standardized	Deaths	Rate	Germany	Both	Encephalitis	0.13	0.14	0.12	0.29	0.32	0.27	2.453(2.028,2.879)
Age-standardized	Deaths	Rate	Ghana	Both	Encephalitis	1.67	2.21	1.26	1.52	2.28	0.97	-0.261(-0.346,-0.176)
Age-standardized	Deaths	Rate	Greece	Both	Encephalitis	0.08	0.09	0.08	0.38	0.41	0.35	6.901(4.578,9.275)
Age-standardized	Deaths	Rate	Greenland	Both	Encephalitis	0.17	0.23	0.14	0.26	0.34	0.14	2.425(1.690,3.166)
Age-standardized	Deaths	Rate	Grenada	Both	Encephalitis	0.48	0.54	0.43	0.21	0.25	0.18	-3.595(-5.335,-1.824)
Age-standardized	Deaths	Rate	Guam	Both	Encephalitis	0.03	0.04	0.02	0.03	0.04	0.01	1.714(1.158,2.274)
Age-standardized	Deaths	Rate	Guatemala	Both	Encephalitis	0.8	0.86	0.74	0.57	0.67	0.47	-1.399(-2.058,-0.736)
Age-standardized	Deaths	Rate	Guinea	Both	Encephalitis	1.8	2.6	1.23	1.46	2	1.03	-0.644(-0.700,-0.587)
Age-standardized	Deaths	Rate	Guinea-Bissau	Both	Encephalitis	2	2.92	1.43	1.65	2.21	1.2	-0.474(-0.561,-0.386)
Age-standardized	Deaths	Rate	Guyana	Both	Encephalitis	1.32	1.51	1.14	0.87	1.11	0.66	-1.165(-1.880,-0.445)
Age-standardized	Deaths	Rate	Haiti	Both	Encephalitis	0.59	0.98	0.37	0.32	0.49	0.19	-1.957(-2.203,-1.709)
Age-standardized	Deaths	Rate	Honduras	Both	Encephalitis	0.72	0.89	0.55	0.58	0.86	0.37	-0.643(-0.845,-0.441)
Age-standardized	Deaths	Rate	Hungary	Both	Encephalitis	0.41	0.45	0.38	0.12	0.15	0.1	-4.797(-5.468,-4.121)
Age-standardized	Deaths	Rate	Iceland	Both	Encephalitis	0.03	0.04	0.03	0.02	0.02	0.01	0.110(-2.989,3.308)
Age-standardized	Deaths	Rate	India	Both	Encephalitis	6.5	7.53	5.36	4.19	5.26	3.49	-1.668(-1.904,-1.432)
Age-standardized	Deaths	Rate	Indonesia	Both	Encephalitis	1.18	1.57	0.77	1.28	1.66	0.81	0.408(0.327,0.490)
Age-standardized	Deaths	Rate	Iran (Islamic Republic of)	Both	Encephalitis	0.33	0.43	0.27	0.26	0.3	0.2	-0.000(-0.322,0.323)
Age-standardized	Deaths	Rate	Iraq	Both	Encephalitis	2.03	2.83	1.46	1.37	1.78	1.02	-1.230(-1.322,-1.138)
Age-standardized	Deaths	Rate	Ireland	Both	Encephalitis	0.06	0.06	0.06	0.09	0.1	0.08	1.583(0.774,2.398)
Age-standardized	Deaths	Rate	Israel	Both	Encephalitis	0.16	0.17	0.15	0.22	0.24	0.19	0.435(-0.610,1.492)
Age-standardized	Deaths	Rate	Italy	Both	Encephalitis	0.29	0.3	0.28	0.4	0.42	0.38	1.081(0.840,1.323)
Age-standardized	Deaths	Rate	Jamaica	Both	Encephalitis	1.13	1.25	1.03	0.55	0.71	0.41	-1.500(-2.074,-0.923)
Age-standardized	Deaths	Rate	Japan	Both	Encephalitis	0.23	0.24	0.23	0.17	0.18	0.16	-2.077(-2.609,-1.542)
Age-standardized	Deaths	Rate	Jordan	Both	Encephalitis	0.38	0.48	0.27	0.1	0.13	0.08	-5.485(-6.326,-4.637)
Age-standardized	Deaths	Rate	Kazakhstan	Both	Encephalitis	1.2	1.5	0.95	0.9	1.2	0.66	-2.410(-3.284,-1.529)
Age-standardized	Deaths	Rate	Kenya	Both	Encephalitis	0.66	0.79	0.54	0.9	1.13	0.7	1.424(1.176,1.673)
Age-standardized	Deaths	Rate	Kiribati	Both	Encephalitis	0.43	0.63	0.28	0.49	0.73	0.28	1.361(0.770,1.956)
Age-standardized	Deaths	Rate	Kuwait	Both	Encephalitis	0.18	0.19	0.16	0.08	0.1	0.07	-1.177(-2.597,0.264)
Age-standardized	Deaths	Rate	Kyrgyzstan	Both	Encephalitis	1.9	2.4	1.49	0.83	1.05	0.66	-3.360(-3.829,-2.888)
Age-standardized	Deaths	Rate	Lao People's Democratic Republic	Both	Encephalitis	0.99	1.84	0.65	1.04	1.45	0.73	0.197(-0.003,0.398)
Age-standardized	Deaths	Rate	Latvia	Both	Encephalitis	1.5	1.69	1.34	0.68	0.79	0.58	-3.706(-4.105,-3.306)
Age-standardized	Deaths	Rate	Lebanon	Both	Encephalitis	0.22	0.31	0.16	0.2	0.25	0.14	-0.084(-0.232,0.065)
Age-standardized	Deaths	Rate	Lesotho	Both	Encephalitis	0.37	0.53	0.23	0.35	0.49	0.25	-0.155(-0.324,0.015)
Age-standardized	Deaths	Rate	Liberia	Both	Encephalitis	1.97	2.67	1.46	1.27	1.88	0.79	-1.403(-1.659,-1.146)
Age-standardized	Deaths	Rate	Libya	Both	Encephalitis	0.26	0.41	0.19	0.24	0.34	0.17	-0.148(-0.214,-0.082)
Age-standardized	Deaths	Rate	Lithuania	Both	Encephalitis	0.72	0.8	0.64	0.63	0.72	0.54	-1.338(-1.850,-0.823)
Age-standardized	Deaths	Rate	Luxembourg	Both	Encephalitis	0.03	0.04	0.03	0.04	0.05	0.04	1.089(-1.064,3.290)
Age-standardized	Deaths	Rate	Madagascar	Both	Encephalitis	1.03	1.38	0.69	0.82	1.21	0.54	-0.771(-0.856,-0.686)
Age-standardized	Deaths	Rate	Malawi	Both	Encephalitis	0.94	1.23	0.64	0.76	1.07	0.51	-0.760(-0.851,-0.670)
Age-standardized	Deaths	Rate	Malaysia	Both	Encephalitis	1.58	1.93	1.2	1.47	1.79	1.09	-0.090(-0.212,0.032)
Age-standardized	Deaths	Rate	Maldives	Both	Encephalitis	0.54	0.82	0.38	0.28	0.39	0.21	-2.202(-2.474,-1.930)
Age-standardized	Deaths	Rate	Mali	Both	Encephalitis	2.11	2.97	1.45	1.47	2.05	1.05	-1.093(-1.265,-0.921)
Age-standardized	Deaths	Rate	Malta	Both	Encephalitis	0.08	0.09	0.07	0.01	0.01	0.01	-7.426(-9.505,-5.299)
Age-standardized	Deaths	Rate	Marshall Islands	Both	Encephalitis	0.09	0.12	0.06	0.08	0.11	0.05	-0.900(-1.052,-0.749)
Age-standardized	Deaths	Rate	Mauritania	Both	Encephalitis	1.74	2.36	1.22	1.31	1.75	0.91	-0.982(-1.068,-0.896)
Age-standardized	Deaths	Rate	Mauritius	Both	Encephalitis	0.42	0.46	0.39	0.82	0.9	0.74	3.145(2.567,3.727)
Age-standardized	Deaths	Rate	Mexico	Both	Encephalitis	0.68	0.73	0.64	0.78	0.88	0.69	0.173(-0.518,0.868)
Age-standardized	Deaths	Rate	Micronesia (Federated States of)	Both	Encephalitis	0.14	0.21	0.09	0.1	0.15	0.07	-1.209(-1.329,-1.088)
Age-standardized	Deaths	Rate	Monaco	Both	Encephalitis	0.08	0.1	0.05	0.03	0.05	0.02	-2.981(-3.660,-2.296)
Age-standardized	Deaths	Rate	Mongolia	Both	Encephalitis	1.76	2.85	1.06	1.36	1.98	0.92	-0.904(-0.978,-0.830)
Age-standardized	Deaths	Rate	Montenegro	Both	Encephalitis	0.06	0.08	0.03	0.03	0.04	0.02	-2.669(-3.002,-2.335)
Age-standardized	Deaths	Rate	Morocco	Both	Encephalitis	0.35	0.56	0.21	0.32	0.45	0.22	-0.101(-0.184,-0.018)
Age-standardized	Deaths	Rate	Mozambique	Both	Encephalitis	0.91	1.25	0.69	0.79	1.17	0.53	-0.350(-0.478,-0.223)
Age-standardized	Deaths	Rate	Myanmar	Both	Encephalitis	0.87	1.35	0.57	0.8	1.15	0.53	-0.290(-0.480,-0.100)
Age-standardized	Deaths	Rate	Namibia	Both	Encephalitis	0.41	0.54	0.3	0.39	0.56	0.27	-0.226(-0.287,-0.165)
Age-standardized	Deaths	Rate	Nauru	Both	Encephalitis	0.11	0.16	0.06	0.2	0.3	0.12	2.893(2.277,3.513)
Age-standardized	Deaths	Rate	Nepal	Both	Encephalitis	6.45	9.31	3.71	3.66	5	2.08	-1.949(-2.141,-1.756)
Age-standardized	Deaths	Rate	Netherlands	Both	Encephalitis	0.17	0.18	0.16	0.15	0.17	0.14	-1.341(-2.005,-0.673)
Age-standardized	Deaths	Rate	New Zealand	Both	Encephalitis	0.03	0.03	0.02	0.2	0.21	0.18	8.232(6.437,10.058)
Age-standardized	Deaths	Rate	Nicaragua	Both	Encephalitis	0.7	0.86	0.52	0.52	0.7	0.37	-0.717(-0.892,-0.543)
Age-standardized	Deaths	Rate	Niger	Both	Encephalitis	2.64	4.01	1.48	1.65	2.45	1.03	-1.607(-1.837,-1.376)
Age-standardized	Deaths	Rate	Nigeria	Both	Encephalitis	1.84	2.45	1.28	1.49	2.04	0.99	-0.554(-0.749,-0.359)
Age-standardized	Deaths	Rate	Niue	Both	Encephalitis	0.1	0.15	0.06	0.08	0.12	0.05	-0.842(-0.897,-0.788)
Age-standardized	Deaths	Rate	North Macedonia	Both	Encephalitis	0.49	0.64	0.36	0.36	0.49	0.25	-1.186(-1.575,-0.796)
Age-standardized	Deaths	Rate	Northern Mariana Islands	Both	Encephalitis	0.1	0.16	0.05	0.07	0.12	0.02	-2.892(-3.392,-2.390)
Age-standardized	Deaths	Rate	Norway	Both	Encephalitis	0.11	0.11	0.1	0.14	0.15	0.12	1.936(1.324,2.552)
Age-standardized	Deaths	Rate	Oman	Both	Encephalitis	4.01	5.63	2.38	1.12	1.68	0.74	-5.089(-5.832,-4.339)
Age-standardized	Deaths	Rate	Pakistan	Both	Encephalitis	4.36	5.72	2.38	4.6	6.48	2.25	0.132(-0.029,0.293)
Age-standardized	Deaths	Rate	Palau	Both	Encephalitis	0.01	0.02	0.01	0.01	0.02	0.01	-0.104(-0.239,0.031)
Age-standardized	Deaths	Rate	Palestine	Both	Encephalitis	0.15	0.23	0.11	0.24	0.3	0.18	2.737(2.091,3.386)
Age-standardized	Deaths	Rate	Panama	Both	Encephalitis	0.8	0.9	0.71	0.26	0.31	0.2	-3.123(-3.843,-2.398)
Age-standardized	Deaths	Rate	Papua New Guinea	Both	Encephalitis	0.45	0.96	0.27	0.52	1.18	0.28	0.500(0.408,0.591)
Age-standardized	Deaths	Rate	Paraguay	Both	Encephalitis	0.46	0.57	0.34	0.38	0.52	0.28	-0.499(-0.678,-0.320)
Age-standardized	Deaths	Rate	Peru	Both	Encephalitis	1.54	1.91	1.23	0.95	1.31	0.67	-1.857(-2.319,-1.393)
Age-standardized	Deaths	Rate	Philippines	Both	Encephalitis	1.18	1.49	0.95	1.13	1.35	0.79	-0.014(-0.146,0.118)
Age-standardized	Deaths	Rate	Poland	Both	Encephalitis	0.69	0.74	0.65	0.28	0.3	0.25	-2.757(-3.260,-2.251)
Age-standardized	Deaths	Rate	Portugal	Both	Encephalitis	0.3	0.31	0.28	0.36	0.39	0.33	0.459(-0.743,1.675)
Age-standardized	Deaths	Rate	Puerto Rico	Both	Encephalitis	1.09	1.16	1.02	0.18	0.2	0.15	-3.646(-5.139,-2.129)
Age-standardized	Deaths	Rate	Qatar	Both	Encephalitis	0.39	0.52	0.23	0.18	0.24	0.1	-2.677(-2.981,-2.371)
Age-standardized	Deaths	Rate	Republic of Korea	Both	Encephalitis	0.2	0.36	0.16	0.29	0.34	0.22	0.870(0.237,1.507)
Age-standardized	Deaths	Rate	Republic of Moldova	Both	Encephalitis	0.49	0.55	0.44	0.64	0.76	0.54	0.273(-0.498,1.050)
Age-standardized	Deaths	Rate	Romania	Both	Encephalitis	1.41	1.52	1.32	0.63	0.72	0.54	-4.024(-4.760,-3.282)
Age-standardized	Deaths	Rate	Russian Federation	Both	Encephalitis	1.03	1.06	1.01	0.84	0.9	0.79	-1.367(-1.765,-0.967)
Age-standardized	Deaths	Rate	Rwanda	Both	Encephalitis	1.04	1.38	0.73	0.78	1.1	0.53	-1.500(-1.749,-1.251)
Age-standardized	Deaths	Rate	Saint Kitts and Nevis	Both	Encephalitis	0.25	0.28	0.23	0.46	0.58	0.35	-0.376(-3.122,2.448)
Age-standardized	Deaths	Rate	Saint Lucia	Both	Encephalitis	1.27	1.4	1.14	0.15	0.19	0.12	-6.806(-8.159,-5.434)
Age-standardized	Deaths	Rate	Saint Vincent and the Grenadines	Both	Encephalitis	0.36	0.41	0.32	0.09	0.11	0.08	-5.784(-7.492,-4.045)
Age-standardized	Deaths	Rate	Samoa	Both	Encephalitis	0.1	0.16	0.06	0.08	0.13	0.05	-0.686(-0.752,-0.620)
Age-standardized	Deaths	Rate	San Marino	Both	Encephalitis	0.33	0.48	0.25	0.26	0.37	0.16	0.546(0.099,0.996)
Age-standardized	Deaths	Rate	Sao Tome and Principe	Both	Encephalitis	2.53	3.27	1.9	2.02	2.97	1.3	-0.583(-0.869,-0.296)
Age-standardized	Deaths	Rate	Saudi Arabia	Both	Encephalitis	1.65	2.26	1.14	0.91	1.22	0.64	-2.500(-2.786,-2.213)
Age-standardized	Deaths	Rate	Senegal	Both	Encephalitis	1.86	2.49	1.37	1.48	2.04	1.02	-0.547(-0.730,-0.364)
Age-standardized	Deaths	Rate	Serbia	Both	Encephalitis	0.81	0.98	0.61	0.67	0.82	0.49	-0.655(-0.818,-0.491)
Age-standardized	Deaths	Rate	Seychelles	Both	Encephalitis	0.15	0.34	0.11	0.42	0.52	0.24	2.751(1.533,3.984)
Age-standardized	Deaths	Rate	Sierra Leone	Both	Encephalitis	1.87	2.57	1.35	1.32	1.88	0.84	-1.297(-1.463,-1.131)
Age-standardized	Deaths	Rate	Singapore	Both	Encephalitis	0.36	0.38	0.33	0.29	0.32	0.27	-0.917(-1.799,-0.027)
Age-standardized	Deaths	Rate	Slovakia	Both	Encephalitis	0.14	0.18	0.12	0.15	0.19	0.11	-0.021(-0.211,0.169)
Age-standardized	Deaths	Rate	Slovenia	Both	Encephalitis	0.16	0.17	0.14	0.13	0.15	0.11	-0.623(-2.068,0.844)
Age-standardized	Deaths	Rate	Solomon Islands	Both	Encephalitis	0.12	0.17	0.07	0.13	0.19	0.08	0.154(0.090,0.219)
Age-standardized	Deaths	Rate	Somalia	Both	Encephalitis	1.09	1.56	0.73	1.01	1.62	0.59	-0.137(-0.262,-0.012)
Age-standardized	Deaths	Rate	South Africa	Both	Encephalitis	0.44	0.5	0.33	0.32	0.4	0.26	-1.137(-1.546,-0.726)
Age-standardized	Deaths	Rate	South Sudan	Both	Encephalitis	1.1	1.57	0.73	1.09	1.58	0.69	-0.165(-0.429,0.100)
Age-standardized	Deaths	Rate	Spain	Both	Encephalitis	0.26	0.27	0.24	0.26	0.29	0.24	0.289(0.038,0.542)
Age-standardized	Deaths	Rate	Sri Lanka	Both	Encephalitis	2.03	2.4	1.67	1.41	1.93	0.98	-1.400(-1.705,-1.093)
Age-standardized	Deaths	Rate	Sudan	Both	Encephalitis	0.44	0.75	0.24	0.38	0.58	0.25	-0.218(-0.339,-0.096)
Age-standardized	Deaths	Rate	Suriname	Both	Encephalitis	0.97	1.14	0.72	0.87	1.15	0.63	-0.356(-0.672,-0.039)
Age-standardized	Deaths	Rate	Sweden	Both	Encephalitis	0.07	0.08	0.07	0.18	0.21	0.16	3.748(3.189,4.311)
Age-standardized	Deaths	Rate	Switzerland	Both	Encephalitis	0.01	0.01	0.01	0.26	0.28	0.22	7.552(4.323,10.881)
Age-standardized	Deaths	Rate	Syrian Arab Republic	Both	Encephalitis	0.59	0.75	0.45	0.53	0.71	0.38	-0.808(-1.287,-0.326)
Age-standardized	Deaths	Rate	Taiwan (Province of China)	Both	Encephalitis	0.29	0.31	0.27	0.32	0.34	0.29	1.158(0.752,1.567)
Age-standardized	Deaths	Rate	Tajikistan	Both	Encephalitis	0.03	0.05	0.01	0.03	0.05	0.01	-0.267(-0.441,-0.092)
Age-standardized	Deaths	Rate	Thailand	Both	Encephalitis	1.36	1.78	0.97	1.05	1.34	0.81	-1.056(-1.286,-0.827)
Age-standardized	Deaths	Rate	Timor-Leste	Both	Encephalitis	1.17	1.87	0.64	1.23	1.75	0.82	-0.006(-0.163,0.150)
Age-standardized	Deaths	Rate	Togo	Both	Encephalitis	1.63	2.09	1.24	1.35	1.99	0.88	-0.707(-0.809,-0.605)
Age-standardized	Deaths	Rate	Tokelau	Both	Encephalitis	0.09	0.13	0.06	0.11	0.17	0.07	0.483(0.304,0.662)
Age-standardized	Deaths	Rate	Tonga	Both	Encephalitis	0.06	0.1	0.03	0.05	0.09	0.03	-0.372(-0.508,-0.235)
Age-standardized	Deaths	Rate	Trinidad and Tobago	Both	Encephalitis	1.74	1.91	1.59	0.27	0.34	0.2	-6.861(-7.362,-6.357)
Age-standardized	Deaths	Rate	Tunisia	Both	Encephalitis	0.3	0.45	0.22	0.27	0.37	0.19	-0.223(-0.326,-0.120)
Age-standardized	Deaths	Rate	Turkey	Both	Encephalitis	0.56	0.74	0.42	0.29	0.37	0.23	-2.225(-2.450,-2.000)
Age-standardized	Deaths	Rate	Turkmenistan	Both	Encephalitis	0.41	0.51	0.33	1.38	1.8	1.04	4.290(3.011,5.585)
Age-standardized	Deaths	Rate	Tuvalu	Both	Encephalitis	0.11	0.15	0.07	0.09	0.13	0.06	-0.373(-0.415,-0.331)
Age-standardized	Deaths	Rate	Uganda	Both	Encephalitis	0.79	1.11	0.51	0.72	1.03	0.49	-0.387(-0.463,-0.311)
Age-standardized	Deaths	Rate	Ukraine	Both	Encephalitis	1.19	1.32	1.08	0.99	1.23	0.76	-0.623(-0.788,-0.457)
Age-standardized	Deaths	Rate	United Arab Emirates	Both	Encephalitis	0.14	0.21	0.08	0.08	0.11	0.05	-1.544(-1.853,-1.233)
Age-standardized	Deaths	Rate	United Kingdom	Both	Encephalitis	0.08	0.08	0.07	0.35	0.37	0.33	5.771(5.392,6.152)
Age-standardized	Deaths	Rate	United Republic of Tanzania	Both	Encephalitis	1.1	1.45	0.74	0.85	1.2	0.56	-0.759(-0.819,-0.699)
Age-standardized	Deaths	Rate	United States of America	Both	Encephalitis	0.12	0.12	0.11	0.19	0.2	0.18	1.899(1.294,2.507)
Age-standardized	Deaths	Rate	United States Virgin Islands	Both	Encephalitis	0	0.01	0	0.01	0.01	0	2.790(1.715,3.877)
Age-standardized	Deaths	Rate	Uruguay	Both	Encephalitis	0.67	0.71	0.62	0.78	0.86	0.71	-0.023(-0.706,0.664)
Age-standardized	Deaths	Rate	Uzbekistan	Both	Encephalitis	1.96	2.49	1.58	1.24	1.53	1	-1.447(-1.871,-1.020)
Age-standardized	Deaths	Rate	Vanuatu	Both	Encephalitis	0.08	0.11	0.05	0.08	0.13	0.05	0.073(-0.101,0.248)
Age-standardized	Deaths	Rate	Venezuela (Bolivarian Republic of)	Both	Encephalitis	1.1	1.16	1.04	0.56	0.71	0.43	-1.746(-2.722,-0.760)
Age-standardized	Deaths	Rate	Viet Nam	Both	Encephalitis	2.24	3.11	1.6	1.82	2.5	1.27	-0.744(-0.797,-0.691)
Age-standardized	Deaths	Rate	Yemen	Both	Encephalitis	0.41	0.65	0.22	0.41	0.59	0.26	0.119(-0.024,0.263)
Age-standardized	Deaths	Rate	Zambia	Both	Encephalitis	0.88	1.14	0.62	1.22	1.8	0.64	1.528(1.232,1.825)
Age-standardized	Deaths	Rate	Zimbabwe	Both	Encephalitis	0.57	0.78	0.31	0.95	1.36	0.44	1.811(1.263,2.361)

ASMR age-standardized mortality rate, *EAPC* estimated annual percentage change, *CI* confidence interval, *UI* uncertainty interval
