# Supplementary material for: Socioeconomic drivers of encephalitis burden in the post-COVID era: a 204-country analysis from global burden of disease study 2021
Source: Front Public Health. 2025 Sep 18;13:1651734. doi: 10.3389/fpubh.2025.1651734 (PMC12488571; doi:10.3389/fpubh.2025.1651734)
Supplement: SUPPLEMENTARY FIGURE S6 — (A) Age-standardized mortality rates of encephalitis for 21 regions by SDI from 1990–2021. The expected values based on the SDI and disease rates at all of the locations are shown as black lines. (B) Age-standardized mortality rates for encephalitis in 204 countries and territories by SDI in 2021. Expected values based on the sociodemographic index and disease rate at all of the locations are shown as black lines. SDI, sociodemographic index. [file Data_Sheet_6.ZIP › supplementary/Figure S24.pdf]

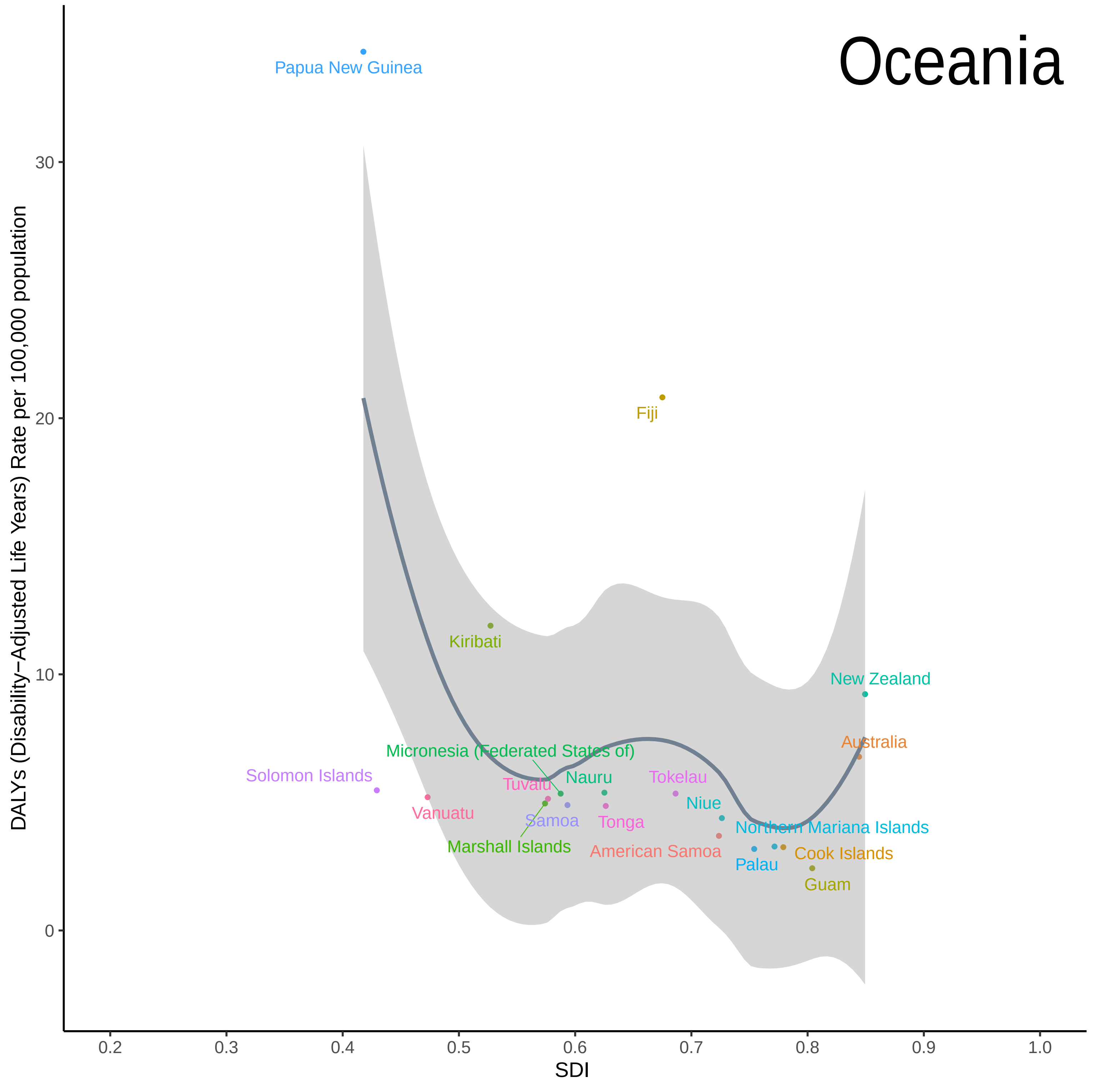

Oceania
Papua New Guinea
Fiji
Kiribati
New Zealand
Australia
Micronesia (Federated States of)
Solomon Islands
Tuvalu
Nauru
Tokelau
Vanuatu
Niue
Samoa
Tonga
Northern Mariana Islands
Marshall Islands
American Samoa
Cook Islands
Palau
Guam
30
20
10
0
0.2
0.3
0.4
0.5
0.6
0.7
0.8
0.9
1.0
DALYs (Disability−Adjusted Life Years) Rate per 100,000 population
SDI
