# Supplementary figures and images for: Socioeconomic drivers of encephalitis burden in the post-COVID era: a 204-country analysis from global burden of disease study 2021
Source: Front Public Health. 2025 Sep 18;13:1651734. doi: 10.3389/fpubh.2025.1651734 (PMC12488571; doi:10.3389/fpubh.2025.1651734)

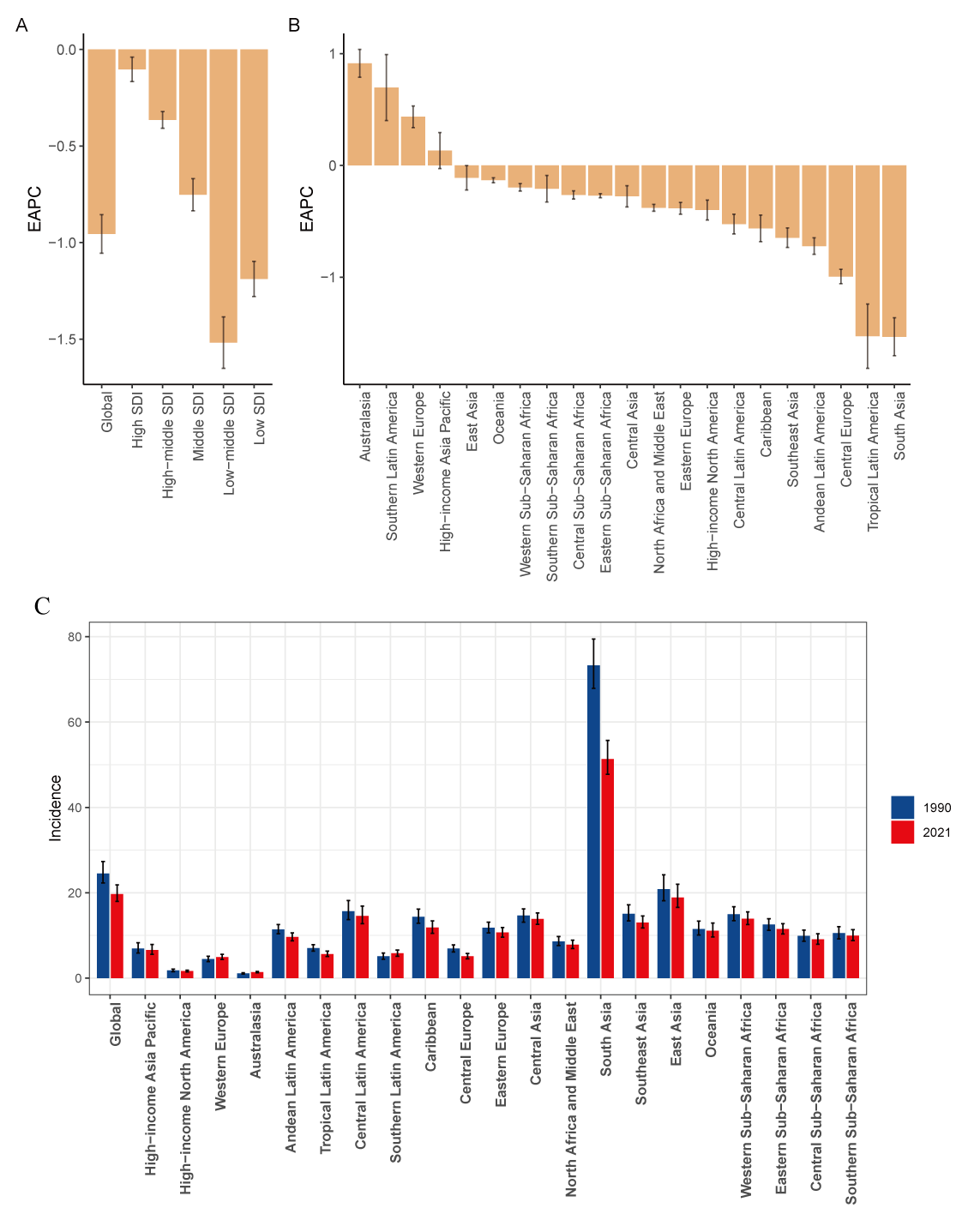

Supplement: SUPPLEMENTARY FIGURE S6 — (A) Age-standardized mortality rates of encephalitis for 21 regions by SDI from 1990–2021. The expected values based on the SDI and disease rates at all of the locations are shown as black lines. (B) Age-standardized mortality rates for encephalitis in 204 countries and territories by SDI in 2021. Expected values based on the sociodemographic index and disease rate at all of the locations are shown as black lines. SDI, sociodemographic index. [file Data_Sheet_6.ZIP › supplementary/Figure S1.tif]

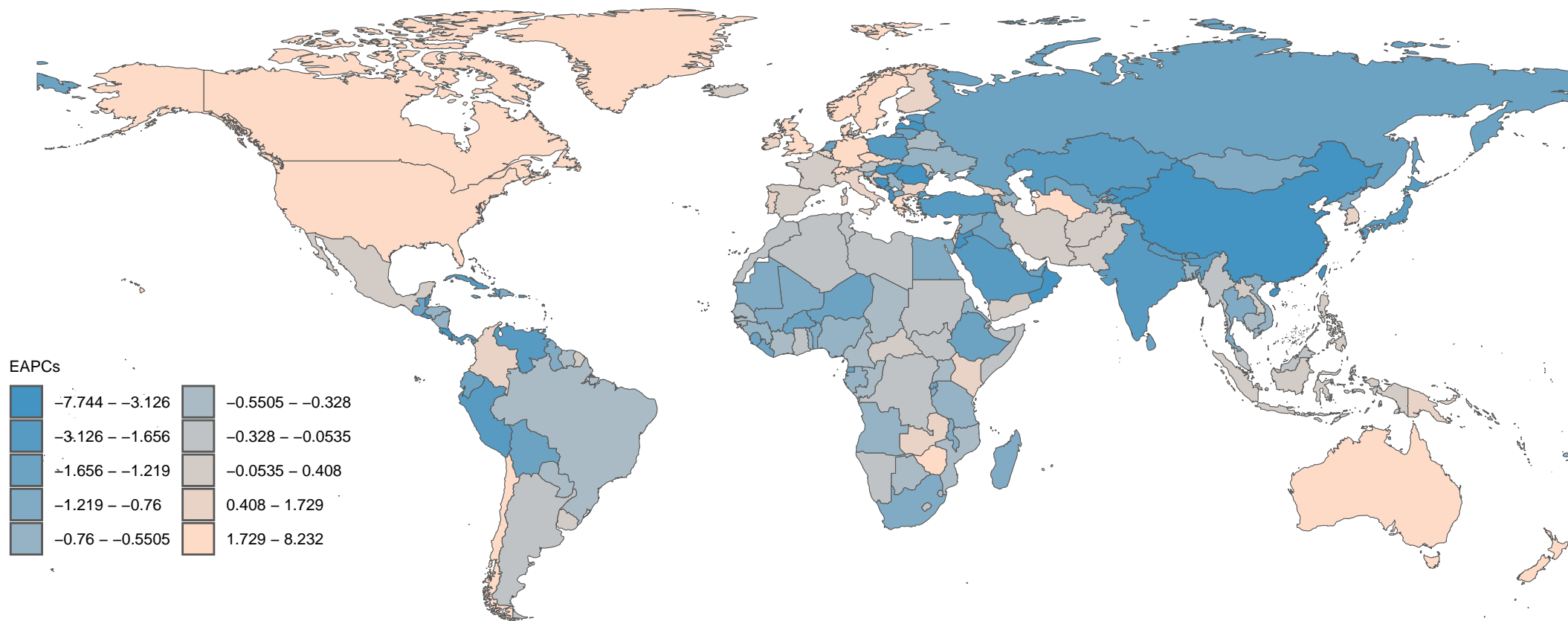

Supplement: SUPPLEMENTARY FIGURE S6 — (A) Age-standardized mortality rates of encephalitis for 21 regions by SDI from 1990–2021. The expected values based on the SDI and disease rates at all of the locations are shown as black lines. (B) Age-standardized mortality rates for encephalitis in 204 countries and territories by SDI in 2021. Expected values based on the sociodemographic index and disease rate at all of the locations are shown as black lines. SDI, sociodemographic index. [file Data_Sheet_6.ZIP › supplementary/Figure s10.pdf]

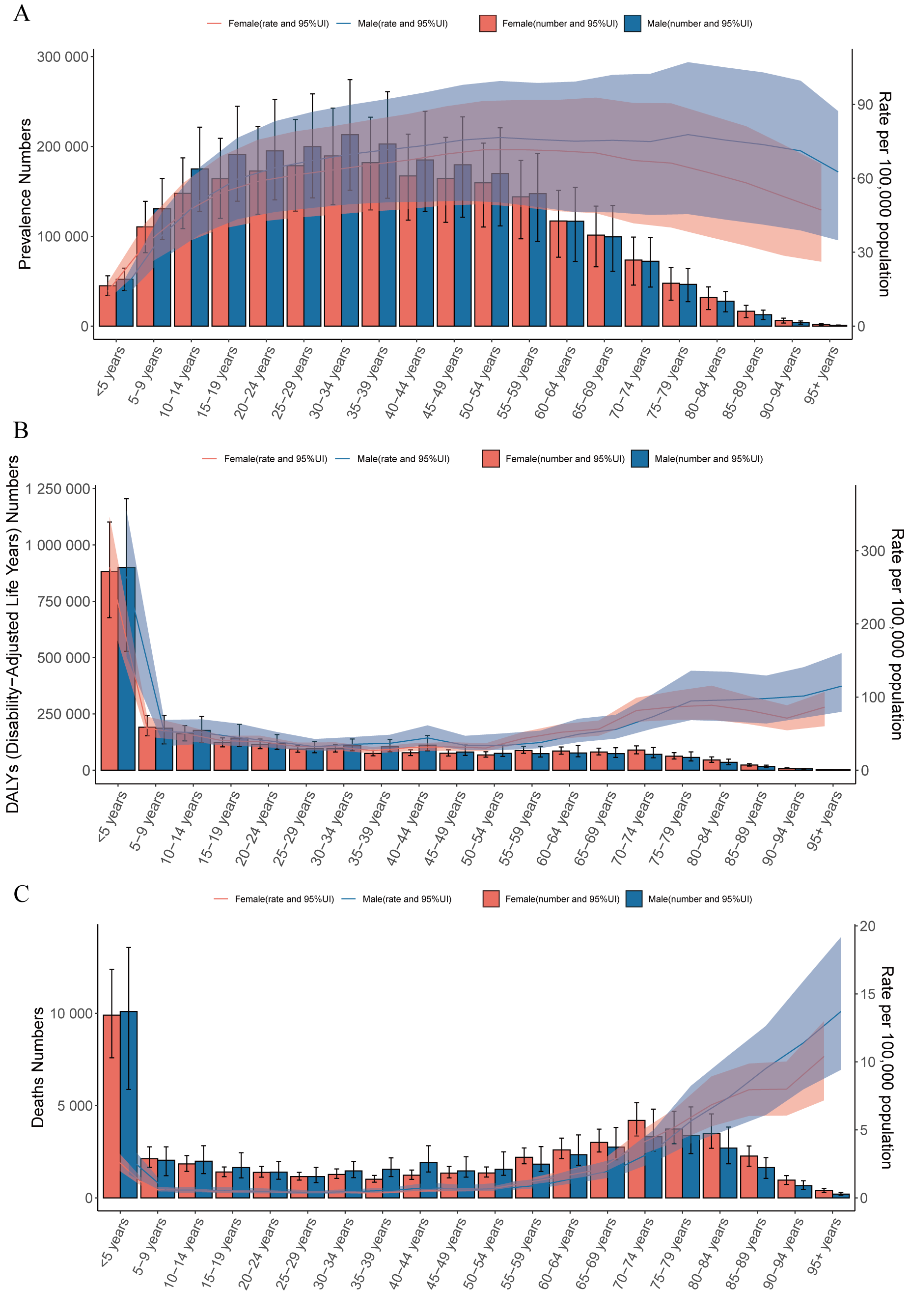

Supplement: SUPPLEMENTARY FIGURE S6 — (A) Age-standardized mortality rates of encephalitis for 21 regions by SDI from 1990–2021. The expected values based on the SDI and disease rates at all of the locations are shown as black lines. (B) Age-standardized mortality rates for encephalitis in 204 countries and territories by SDI in 2021. Expected values based on the sociodemographic index and disease rate at all of the locations are shown as black lines. SDI, sociodemographic index. [file Data_Sheet_6.ZIP › supplementary/Figure S11.tif]

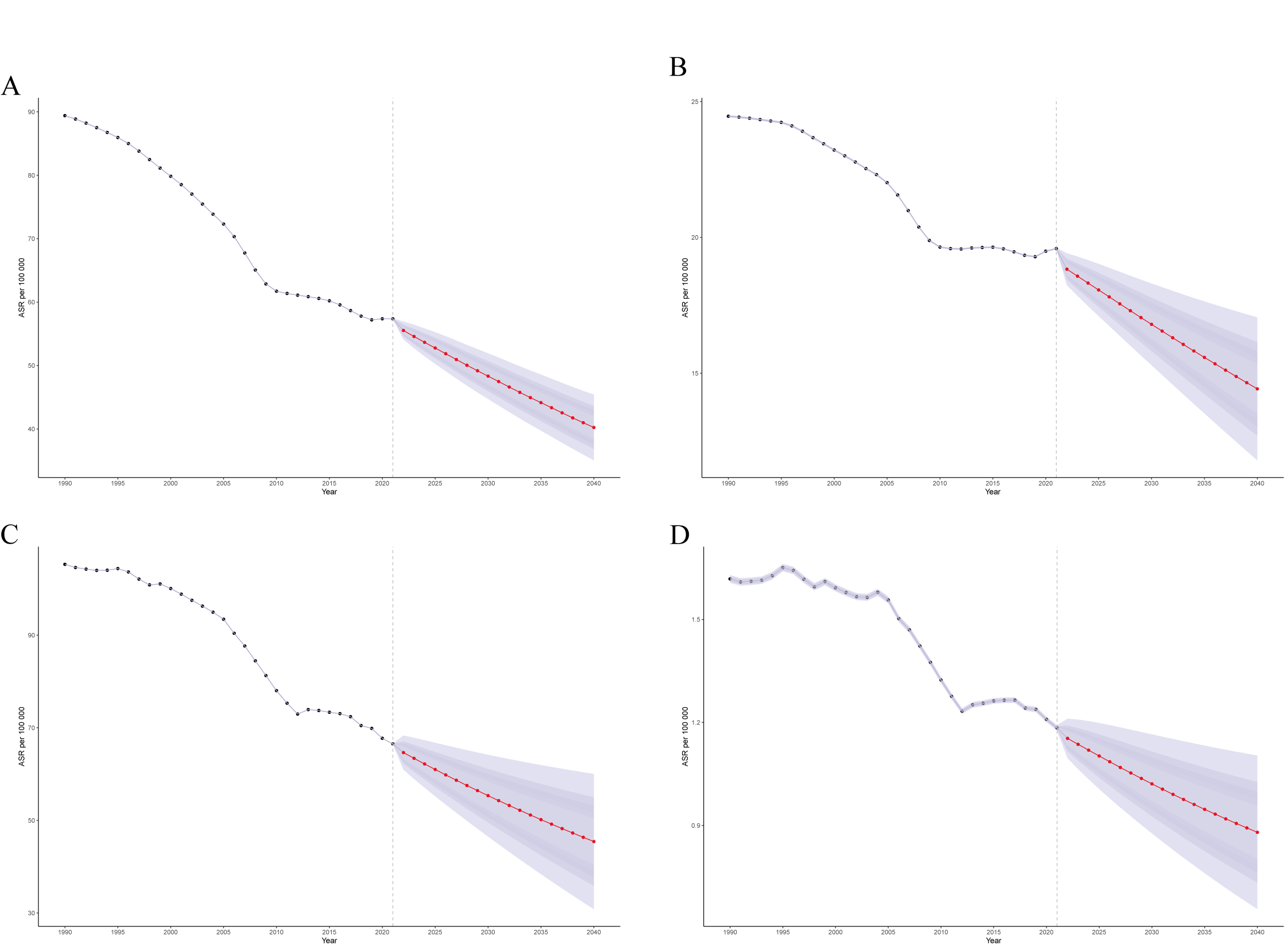

Supplement: SUPPLEMENTARY FIGURE S6 — (A) Age-standardized mortality rates of encephalitis for 21 regions by SDI from 1990–2021. The expected values based on the SDI and disease rates at all of the locations are shown as black lines. (B) Age-standardized mortality rates for encephalitis in 204 countries and territories by SDI in 2021. Expected values based on the sociodemographic index and disease rate at all of the locations are shown as black lines. SDI, sociodemographic index. [file Data_Sheet_6.ZIP › supplementary/Figure S12.tif]

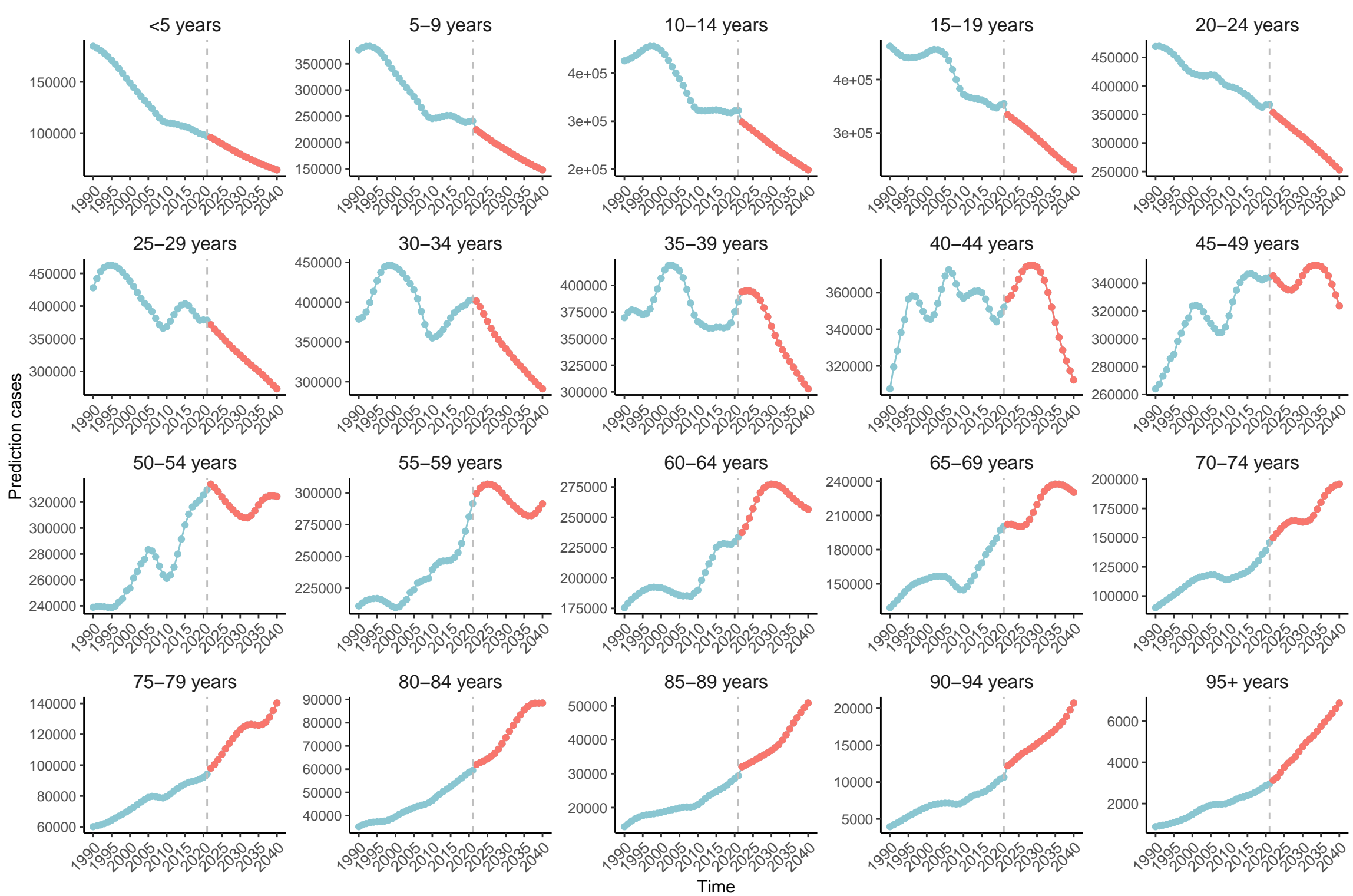

Supplement: SUPPLEMENTARY FIGURE S6 — (A) Age-standardized mortality rates of encephalitis for 21 regions by SDI from 1990–2021. The expected values based on the SDI and disease rates at all of the locations are shown as black lines. (B) Age-standardized mortality rates for encephalitis in 204 countries and territories by SDI in 2021. Expected values based on the sociodemographic index and disease rate at all of the locations are shown as black lines. SDI, sociodemographic index. [file Data_Sheet_6.ZIP › supplementary/Figure S13.pdf]

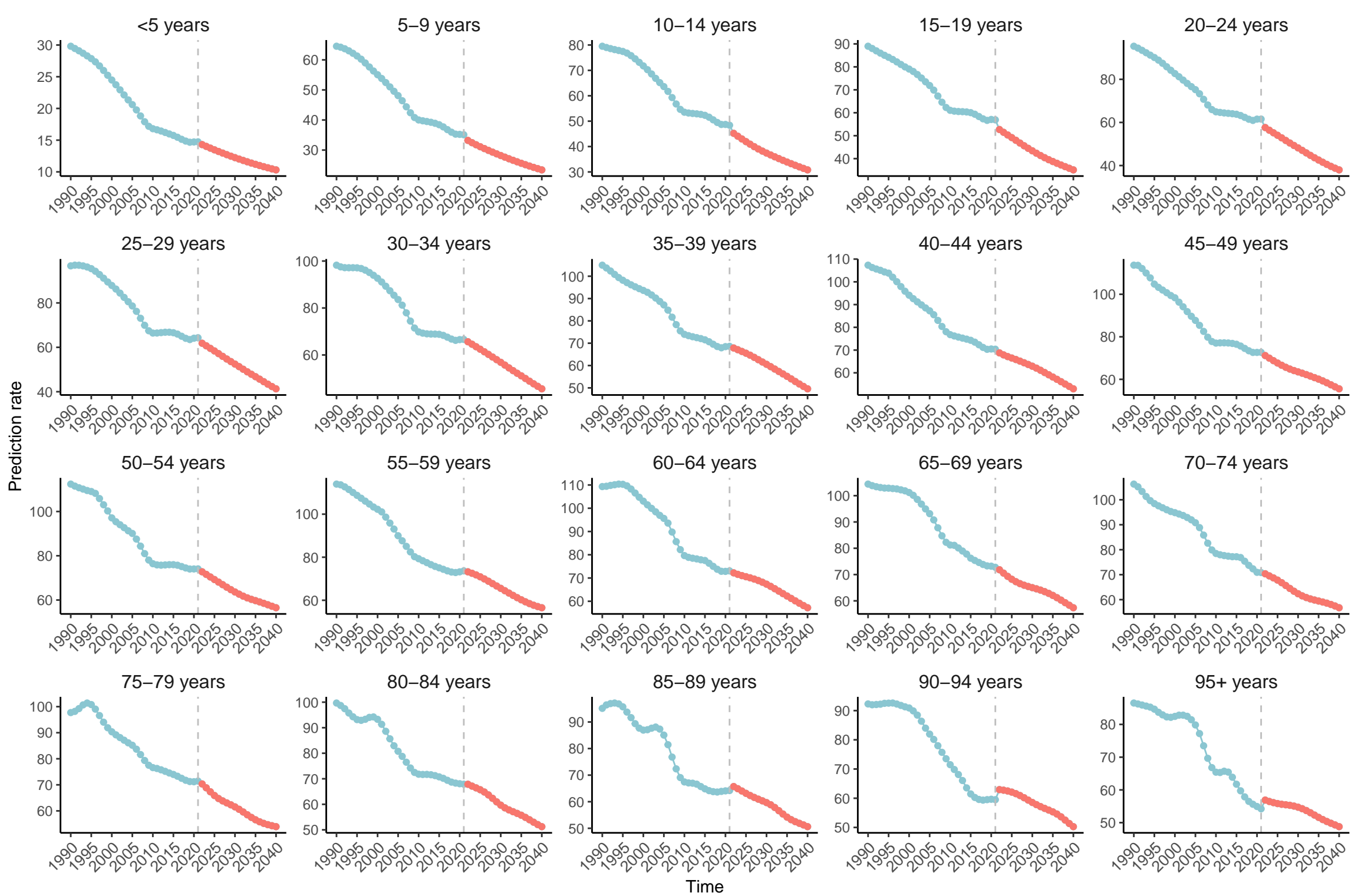

Supplement: SUPPLEMENTARY FIGURE S6 — (A) Age-standardized mortality rates of encephalitis for 21 regions by SDI from 1990–2021. The expected values based on the SDI and disease rates at all of the locations are shown as black lines. (B) Age-standardized mortality rates for encephalitis in 204 countries and territories by SDI in 2021. Expected values based on the sociodemographic index and disease rate at all of the locations are shown as black lines. SDI, sociodemographic index. [file Data_Sheet_6.ZIP › supplementary/Figure S14.pdf]

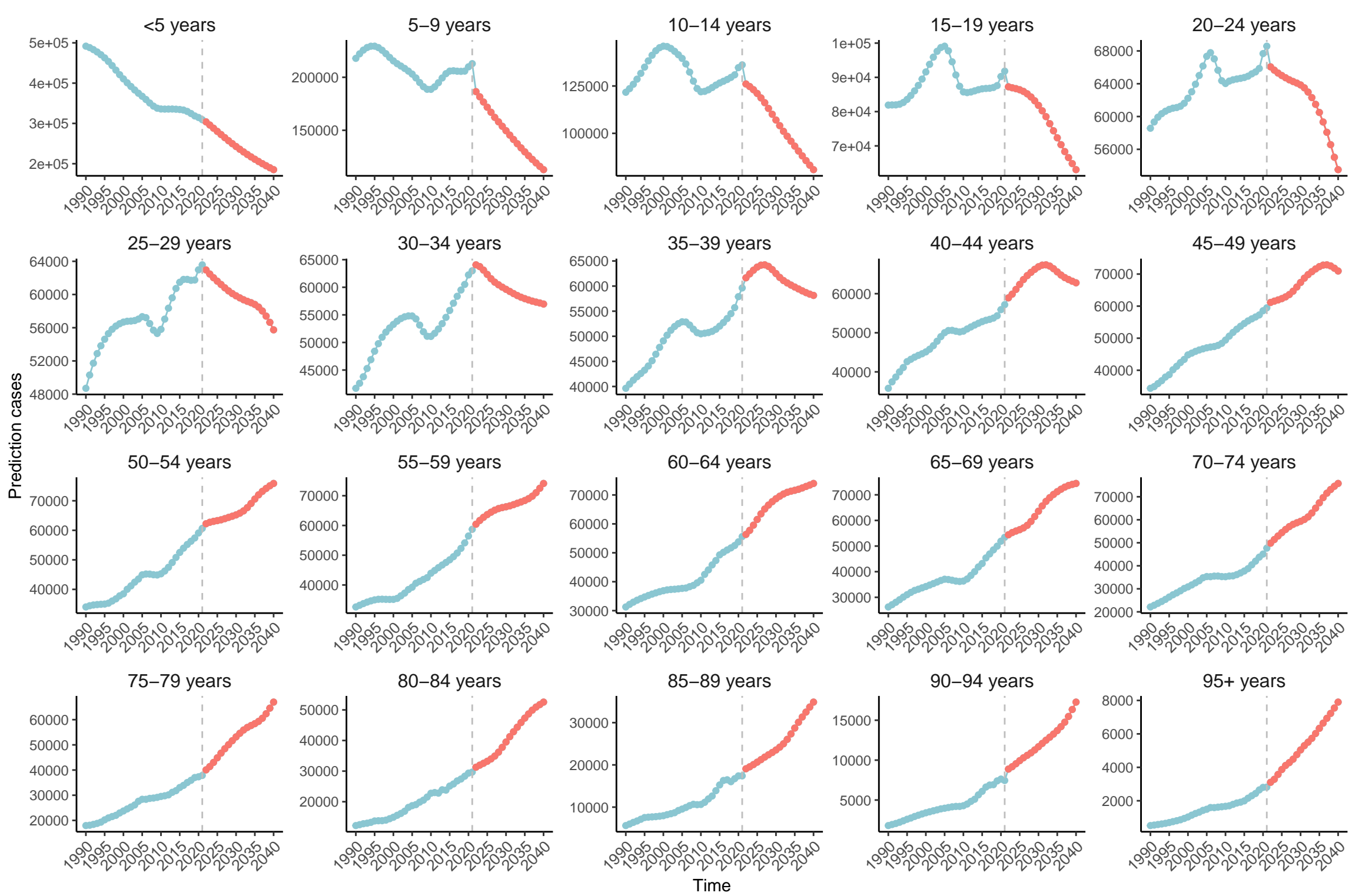

Supplement: SUPPLEMENTARY FIGURE S6 — (A) Age-standardized mortality rates of encephalitis for 21 regions by SDI from 1990–2021. The expected values based on the SDI and disease rates at all of the locations are shown as black lines. (B) Age-standardized mortality rates for encephalitis in 204 countries and territories by SDI in 2021. Expected values based on the sociodemographic index and disease rate at all of the locations are shown as black lines. SDI, sociodemographic index. [file Data_Sheet_6.ZIP › supplementary/Figure S15.pdf]

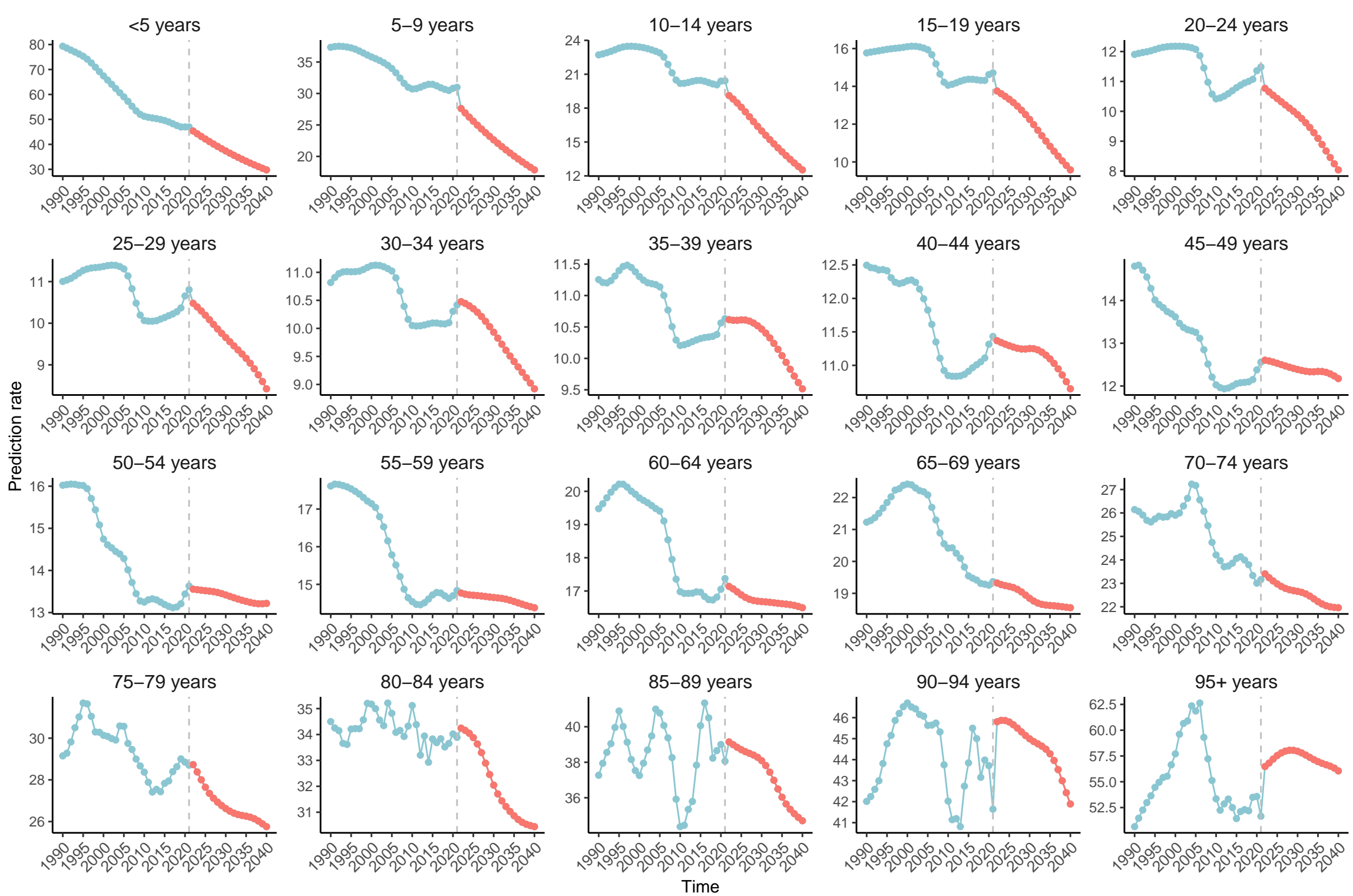

Supplement: SUPPLEMENTARY FIGURE S6 — (A) Age-standardized mortality rates of encephalitis for 21 regions by SDI from 1990–2021. The expected values based on the SDI and disease rates at all of the locations are shown as black lines. (B) Age-standardized mortality rates for encephalitis in 204 countries and territories by SDI in 2021. Expected values based on the sociodemographic index and disease rate at all of the locations are shown as black lines. SDI, sociodemographic index. [file Data_Sheet_6.ZIP › supplementary/Figure S16.pdf]

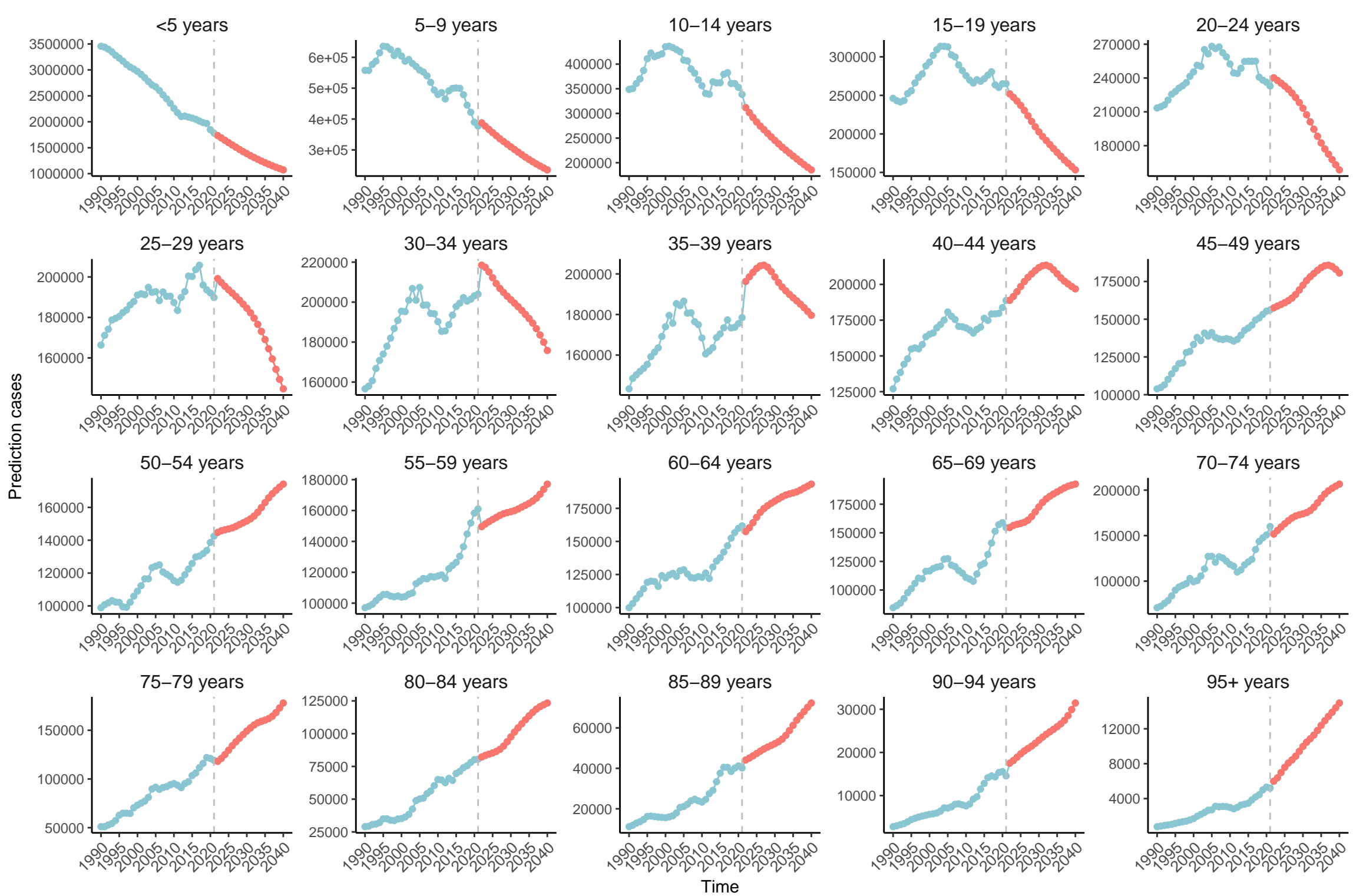

Supplement: SUPPLEMENTARY FIGURE S6 — (A) Age-standardized mortality rates of encephalitis for 21 regions by SDI from 1990–2021. The expected values based on the SDI and disease rates at all of the locations are shown as black lines. (B) Age-standardized mortality rates for encephalitis in 204 countries and territories by SDI in 2021. Expected values based on the sociodemographic index and disease rate at all of the locations are shown as black lines. SDI, sociodemographic index. [file Data_Sheet_6.ZIP › supplementary/Figure S17.pdf]

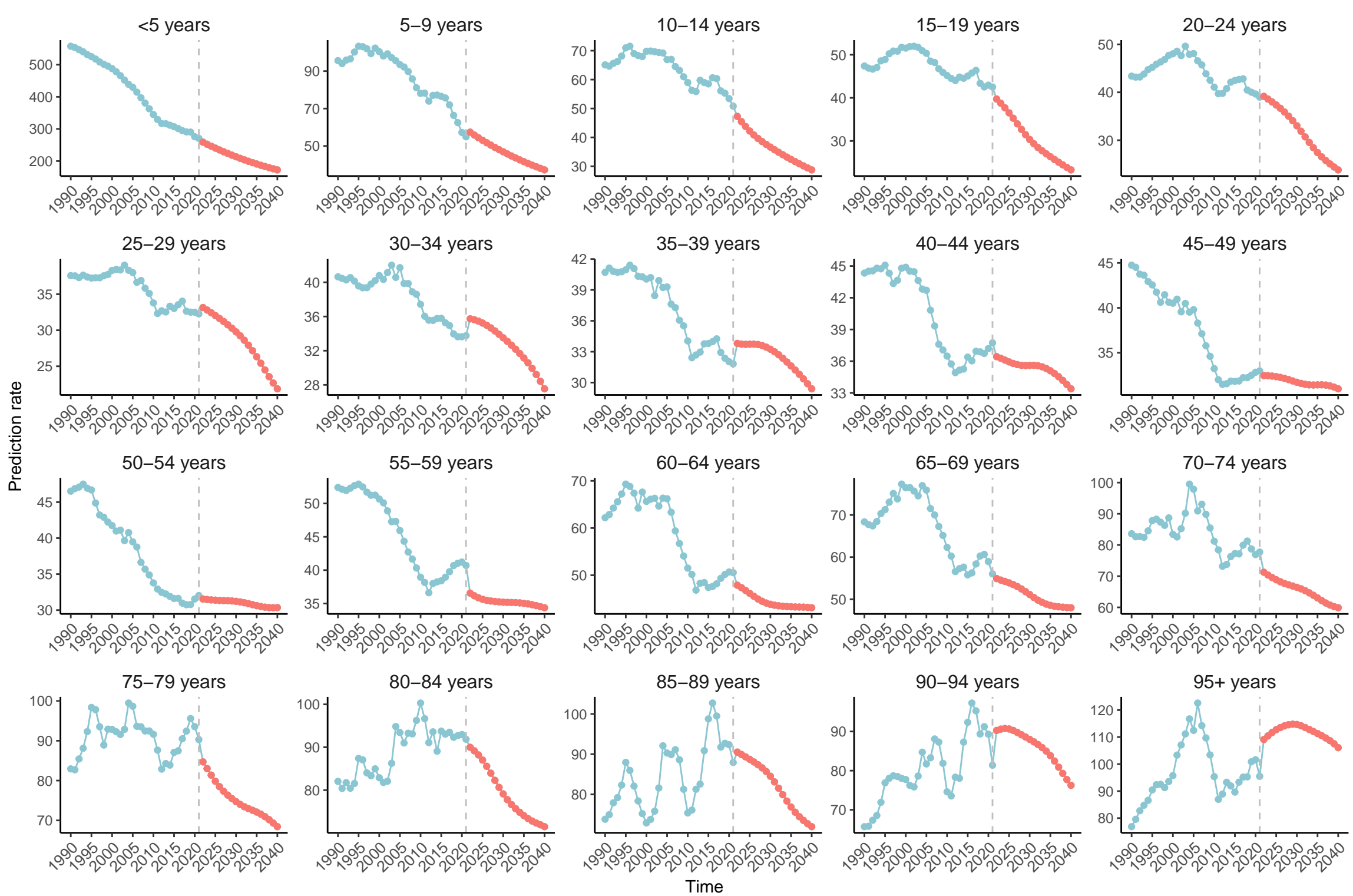

Supplement: SUPPLEMENTARY FIGURE S6 — (A) Age-standardized mortality rates of encephalitis for 21 regions by SDI from 1990–2021. The expected values based on the SDI and disease rates at all of the locations are shown as black lines. (B) Age-standardized mortality rates for encephalitis in 204 countries and territories by SDI in 2021. Expected values based on the sociodemographic index and disease rate at all of the locations are shown as black lines. SDI, sociodemographic index. [file Data_Sheet_6.ZIP › supplementary/Figure S18.pdf]

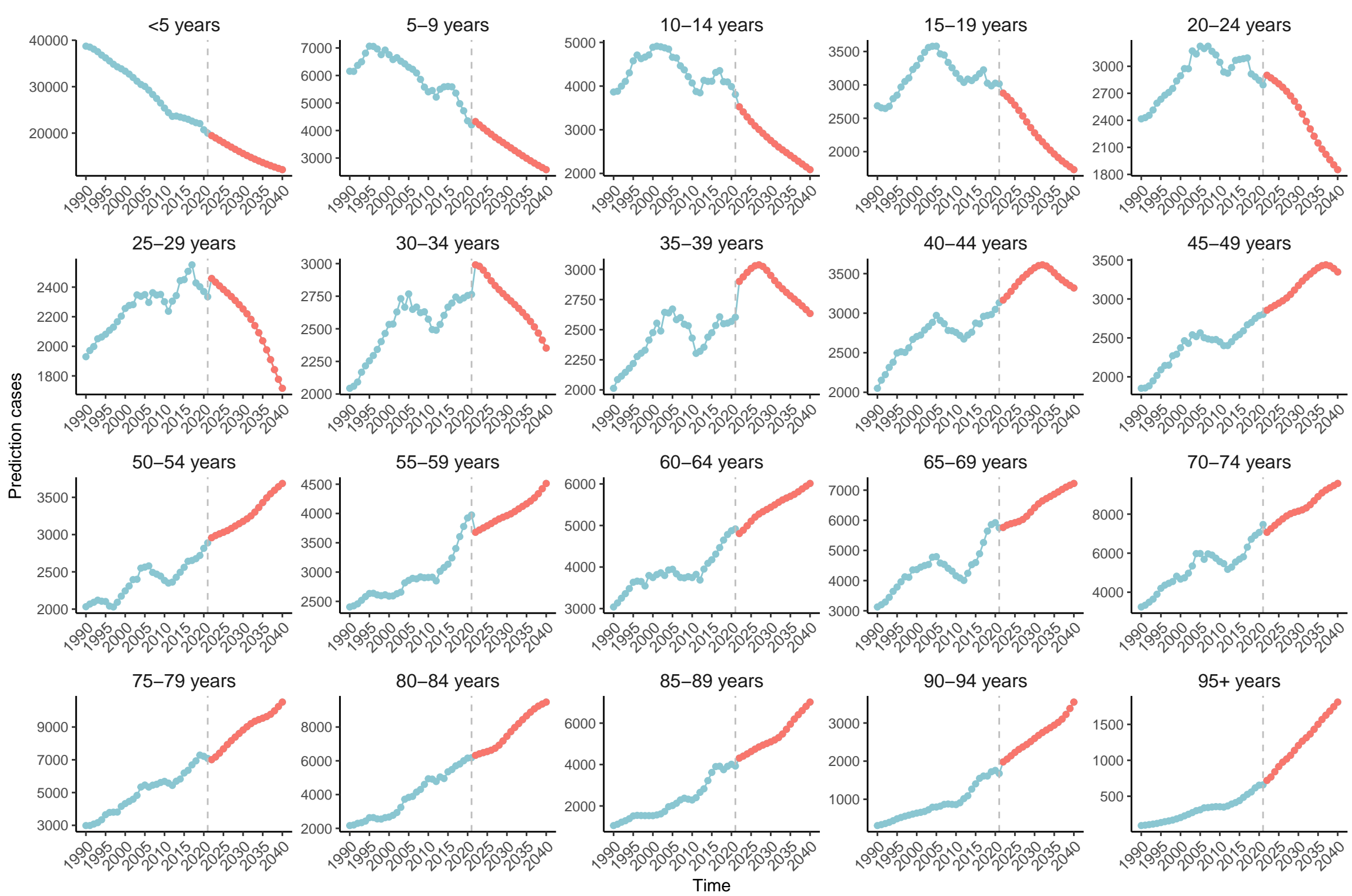

Supplement: SUPPLEMENTARY FIGURE S6 — (A) Age-standardized mortality rates of encephalitis for 21 regions by SDI from 1990–2021. The expected values based on the SDI and disease rates at all of the locations are shown as black lines. (B) Age-standardized mortality rates for encephalitis in 204 countries and territories by SDI in 2021. Expected values based on the sociodemographic index and disease rate at all of the locations are shown as black lines. SDI, sociodemographic index. [file Data_Sheet_6.ZIP › supplementary/Figure S19.pdf]

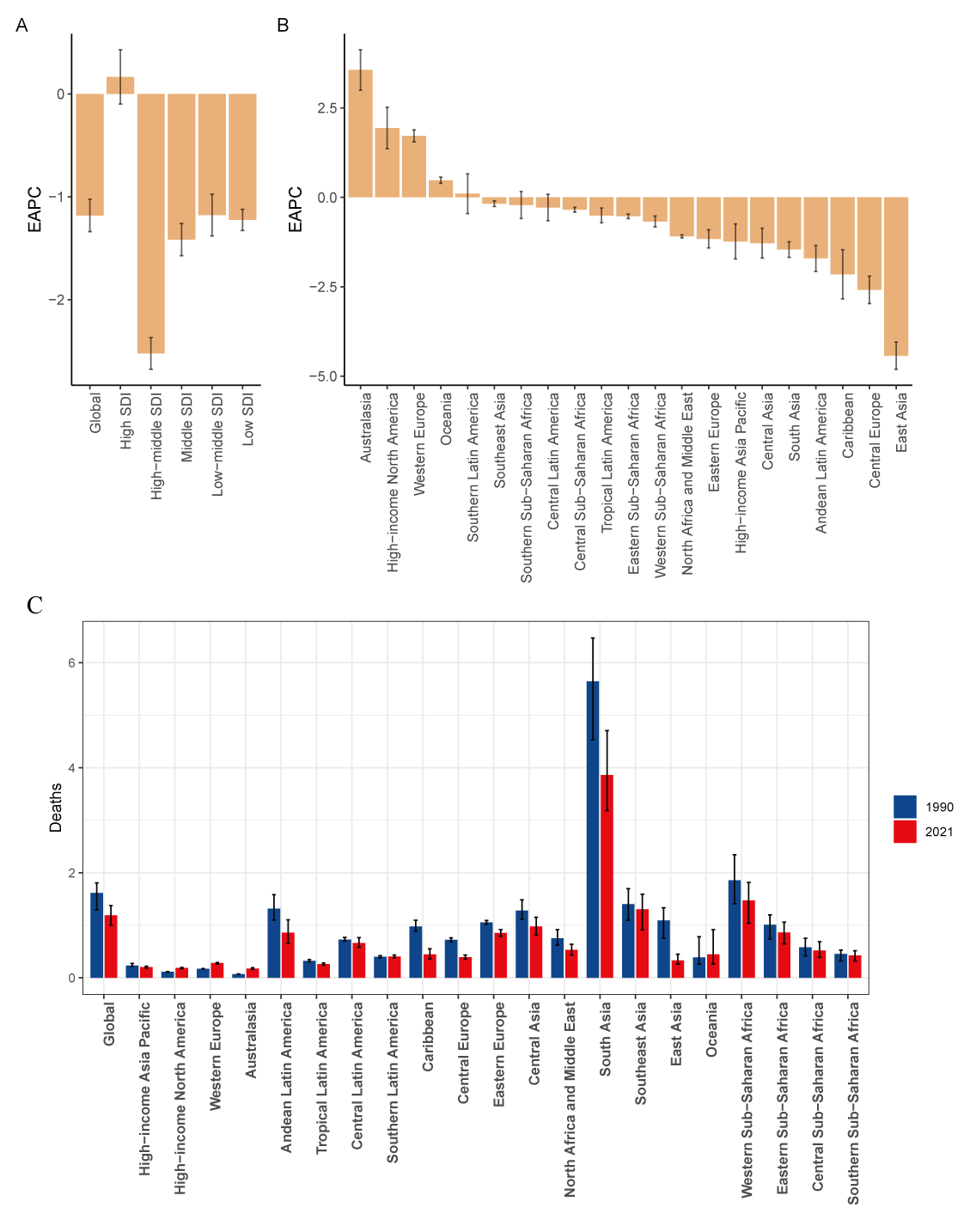

Supplement: SUPPLEMENTARY FIGURE S6 — (A) Age-standardized mortality rates of encephalitis for 21 regions by SDI from 1990–2021. The expected values based on the SDI and disease rates at all of the locations are shown as black lines. (B) Age-standardized mortality rates for encephalitis in 204 countries and territories by SDI in 2021. Expected values based on the sociodemographic index and disease rate at all of the locations are shown as black lines. SDI, sociodemographic index. [file Data_Sheet_6.ZIP › supplementary/Figure S2.tif]

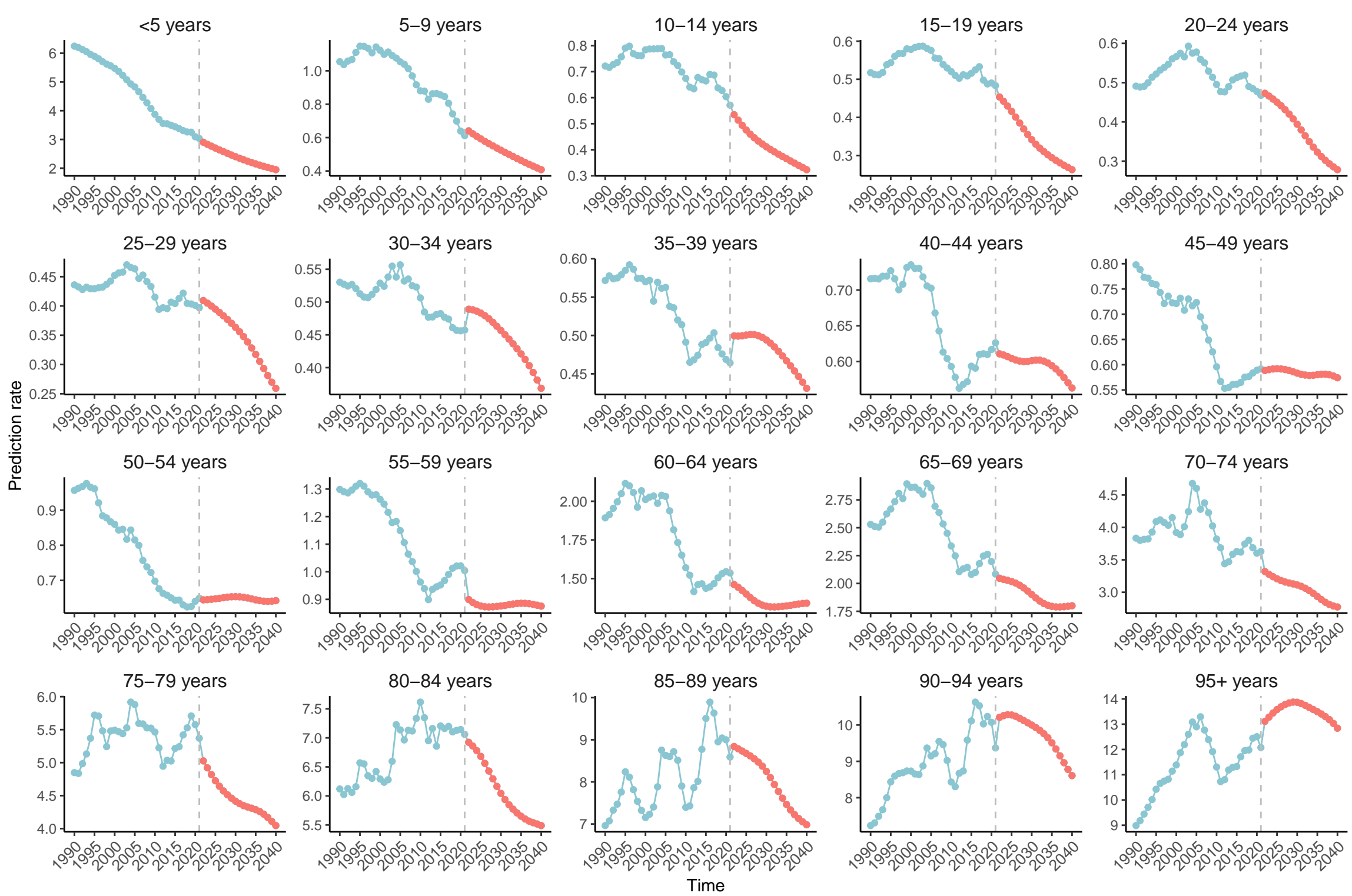

Supplement: SUPPLEMENTARY FIGURE S6 — (A) Age-standardized mortality rates of encephalitis for 21 regions by SDI from 1990–2021. The expected values based on the SDI and disease rates at all of the locations are shown as black lines. (B) Age-standardized mortality rates for encephalitis in 204 countries and territories by SDI in 2021. Expected values based on the sociodemographic index and disease rate at all of the locations are shown as black lines. SDI, sociodemographic index. [file Data_Sheet_6.ZIP › supplementary/Figure S20.pdf]

# Asia

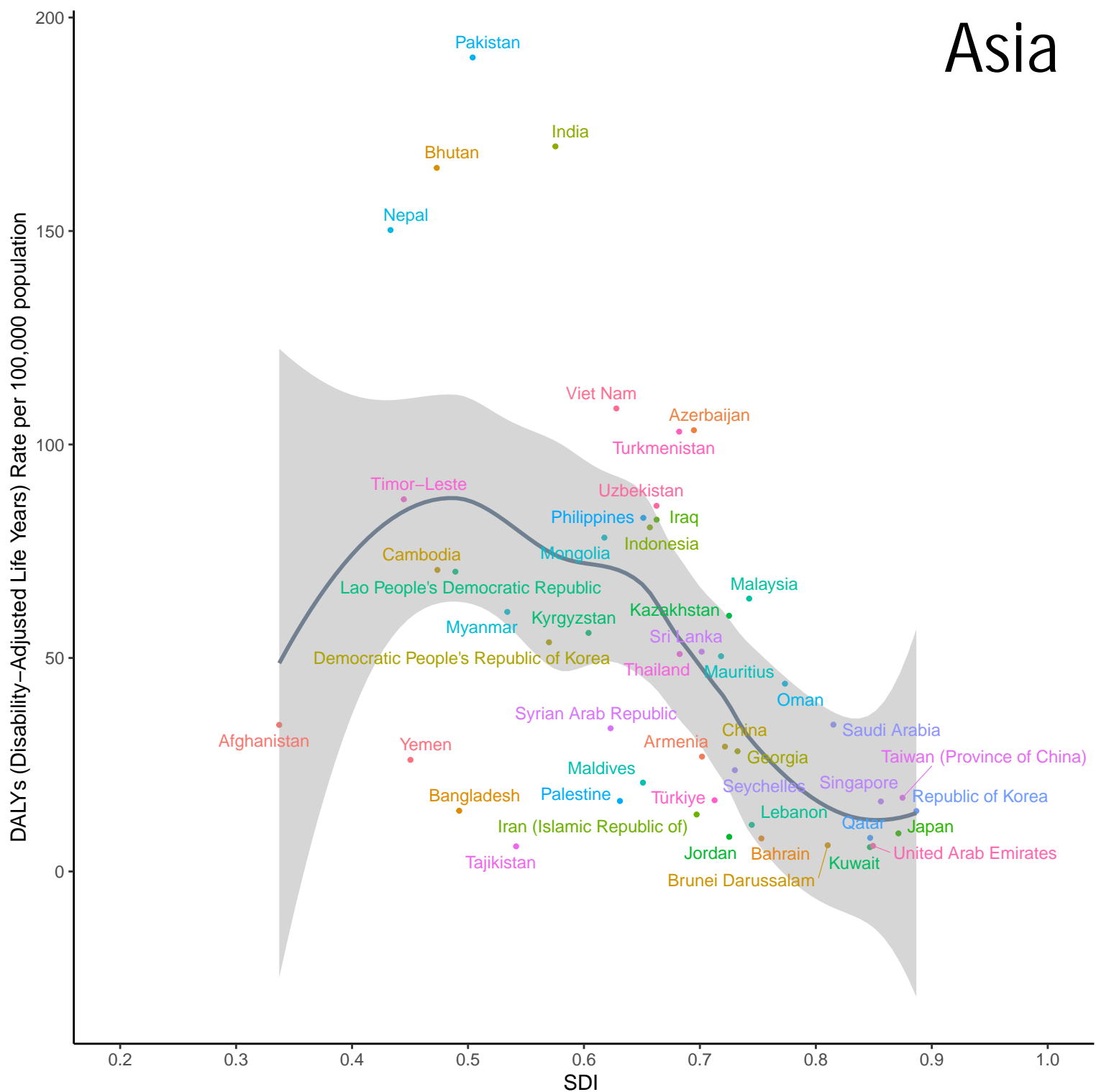

Supplement: SUPPLEMENTARY FIGURE S6 — (A) Age-standardized mortality rates of encephalitis for 21 regions by SDI from 1990–2021. The expected values based on the SDI and disease rates at all of the locations are shown as black lines. (B) Age-standardized mortality rates for encephalitis in 204 countries and territories by SDI in 2021. Expected values based on the sociodemographic index and disease rate at all of the locations are shown as black lines. SDI, sociodemographic index. [file Data_Sheet_6.ZIP › supplementary/Figure S21.pdf]

# Europe

DALYs (Disability-Adjusted Life Years) Rate per 100,000 population

60

40

20

0

0.2

0.3

0.4

0.5

SDI

0.7

0.8

0.9

1.0

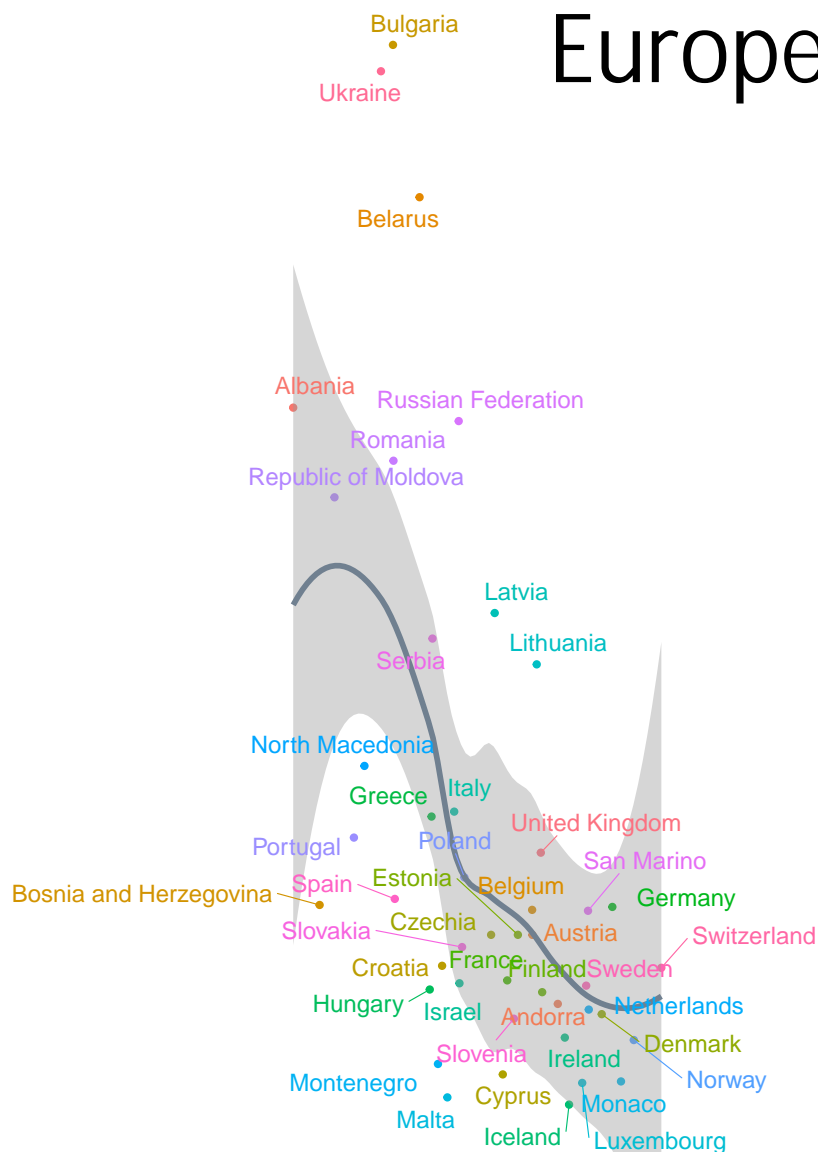

Supplement: SUPPLEMENTARY FIGURE S6 — (A) Age-standardized mortality rates of encephalitis for 21 regions by SDI from 1990–2021. The expected values based on the SDI and disease rates at all of the locations are shown as black lines. (B) Age-standardized mortality rates for encephalitis in 204 countries and territories by SDI in 2021. Expected values based on the sociodemographic index and disease rate at all of the locations are shown as black lines. SDI, sociodemographic index. [file Data_Sheet_6.ZIP › supplementary/Figure S22.pdf]

# Africa

DALYs (Disability-Adjusted Life Years) Rate per 100,000 population

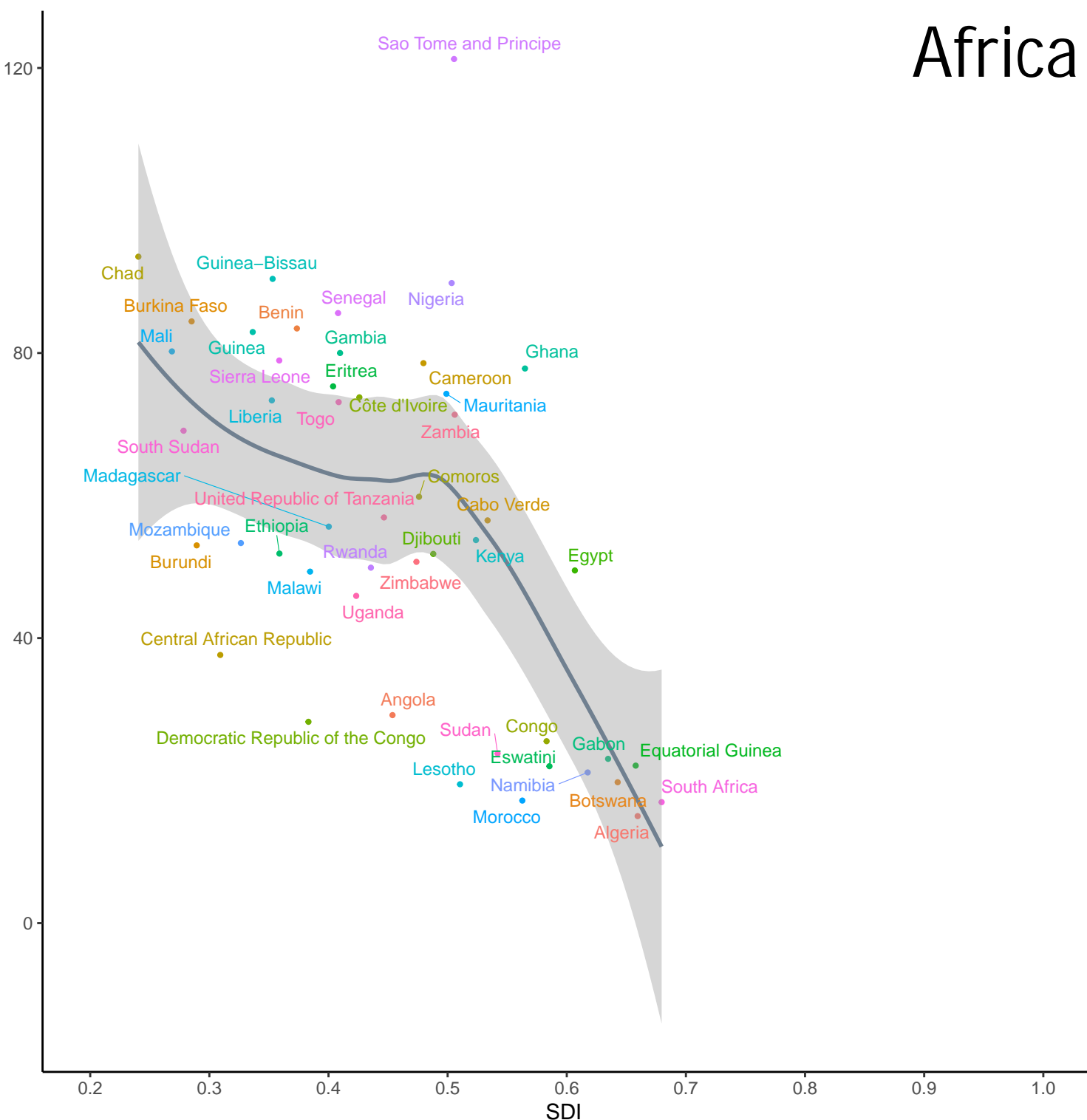

Supplement: SUPPLEMENTARY FIGURE S6 — (A) Age-standardized mortality rates of encephalitis for 21 regions by SDI from 1990–2021. The expected values based on the SDI and disease rates at all of the locations are shown as black lines. (B) Age-standardized mortality rates for encephalitis in 204 countries and territories by SDI in 2021. Expected values based on the sociodemographic index and disease rate at all of the locations are shown as black lines. SDI, sociodemographic index. [file Data_Sheet_6.ZIP › supplementary/Figure S23.pdf]

# North America

DALYs (Disability-Adjusted Life Years) Rate per 100,000 population

0.2

0.3

0.4

0.5

SDI

0.6

0.7

0.8

0.9

1.0

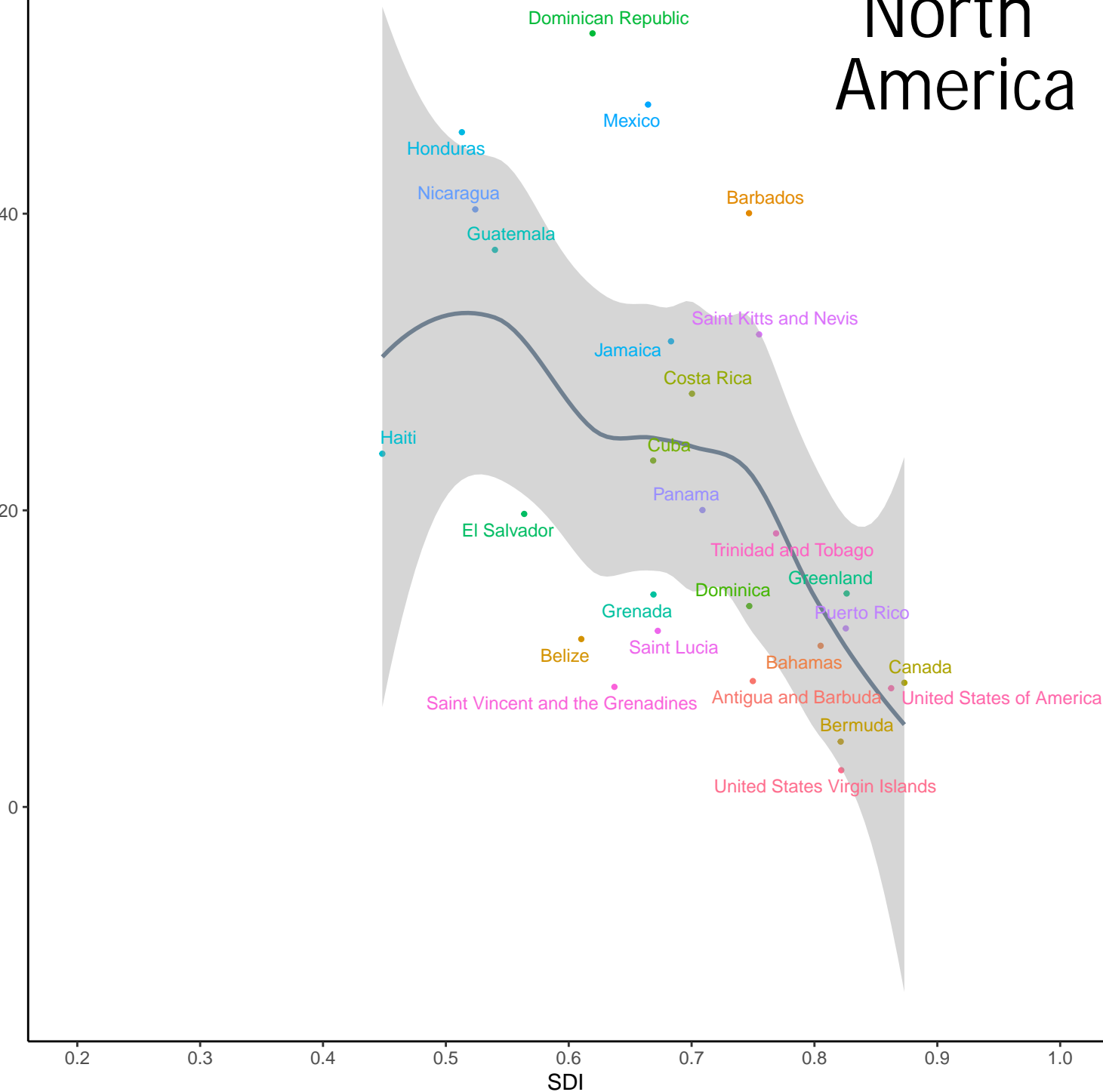

Supplement: SUPPLEMENTARY FIGURE S6 — (A) Age-standardized mortality rates of encephalitis for 21 regions by SDI from 1990–2021. The expected values based on the SDI and disease rates at all of the locations are shown as black lines. (B) Age-standardized mortality rates for encephalitis in 204 countries and territories by SDI in 2021. Expected values based on the sociodemographic index and disease rate at all of the locations are shown as black lines. SDI, sociodemographic index. [file Data_Sheet_6.ZIP › supplementary/Figure S25.pdf]

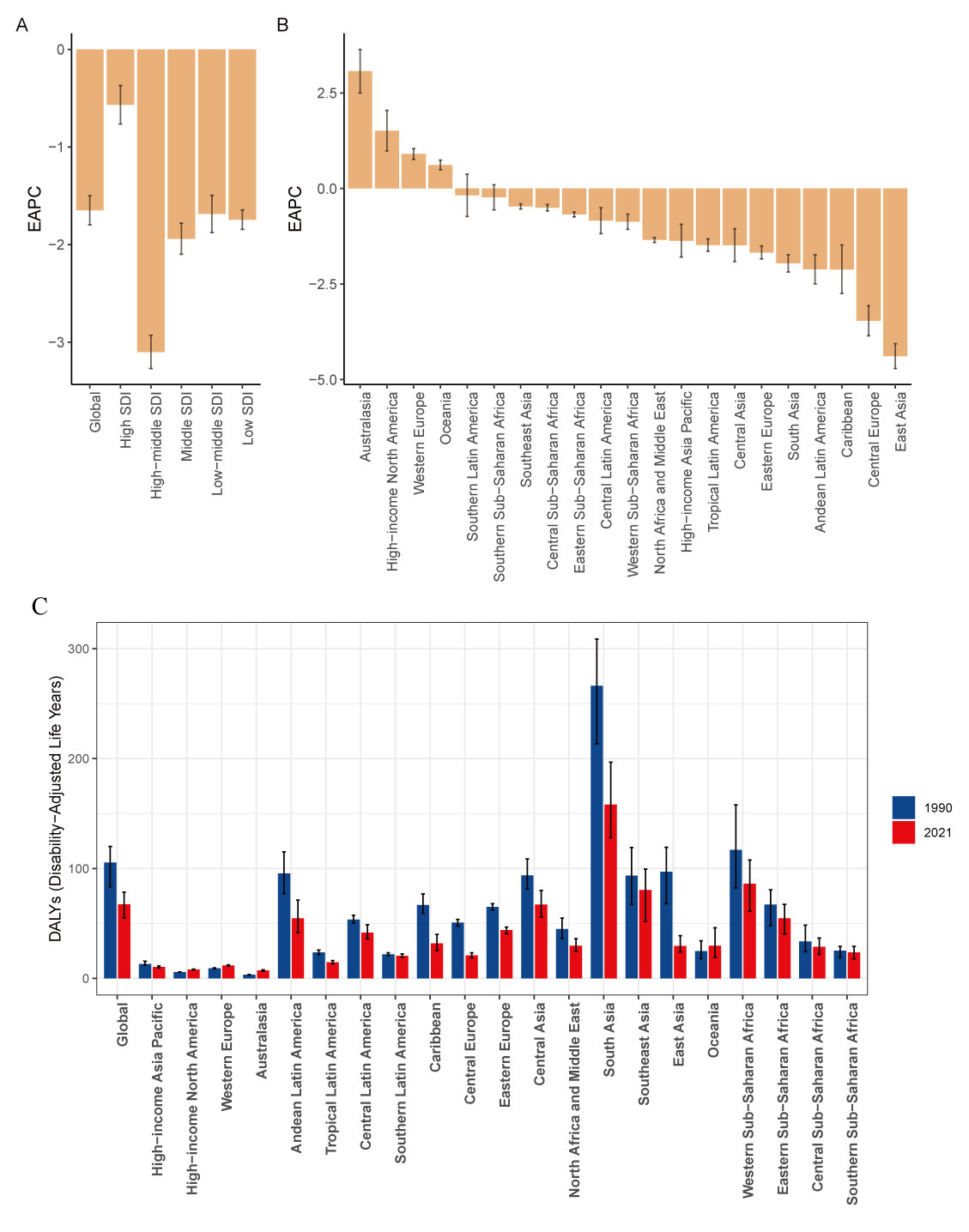

Supplement: SUPPLEMENTARY FIGURE S6 — (A) Age-standardized mortality rates of encephalitis for 21 regions by SDI from 1990–2021. The expected values based on the SDI and disease rates at all of the locations are shown as black lines. (B) Age-standardized mortality rates for encephalitis in 204 countries and territories by SDI in 2021. Expected values based on the sociodemographic index and disease rate at all of the locations are shown as black lines. SDI, sociodemographic index. [file Data_Sheet_6.ZIP › supplementary/Figure S3.tif]

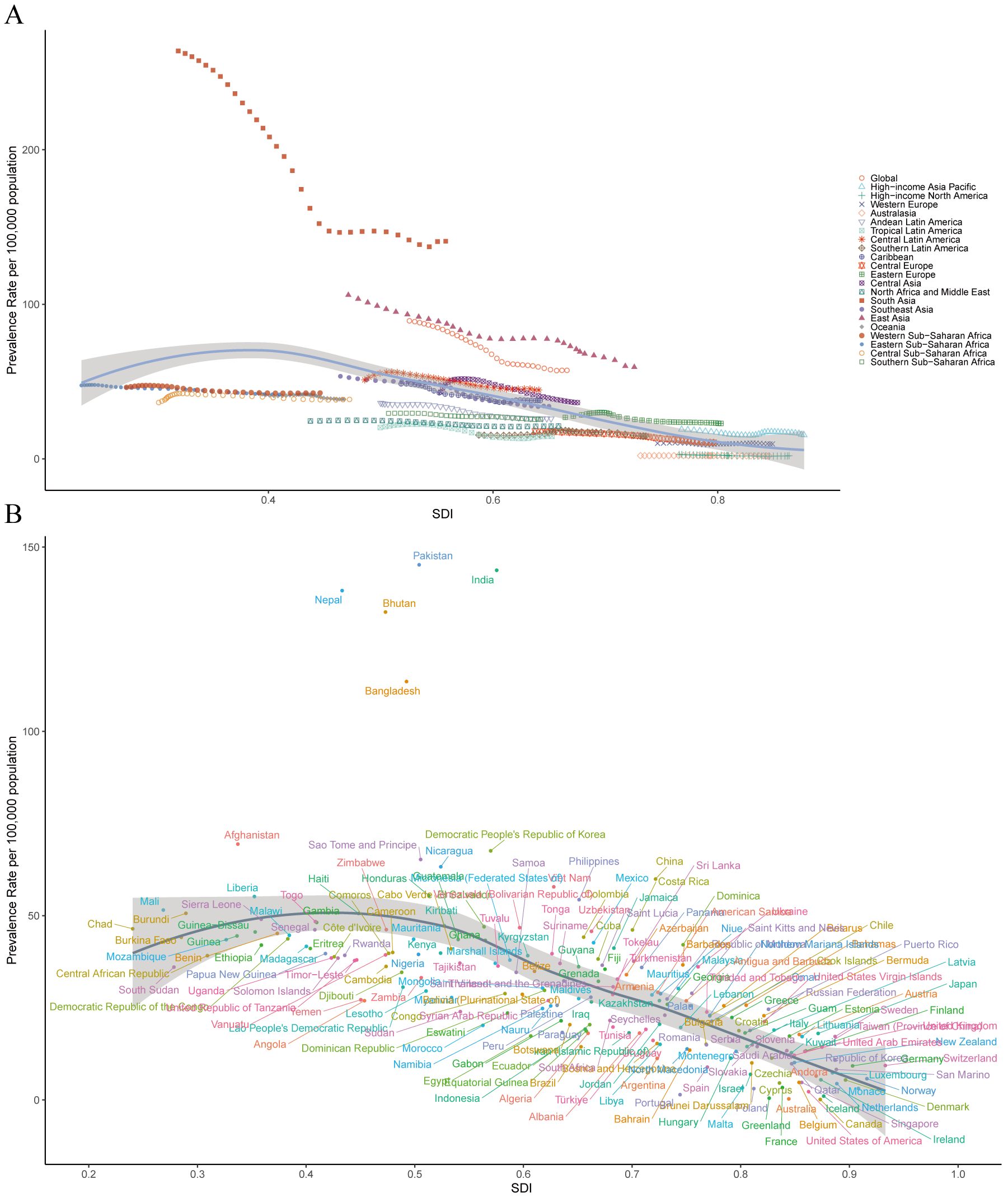

Supplement: SUPPLEMENTARY FIGURE S6 — (A) Age-standardized mortality rates of encephalitis for 21 regions by SDI from 1990–2021. The expected values based on the SDI and disease rates at all of the locations are shown as black lines. (B) Age-standardized mortality rates for encephalitis in 204 countries and territories by SDI in 2021. Expected values based on the sociodemographic index and disease rate at all of the locations are shown as black lines. SDI, sociodemographic index. [file Data_Sheet_6.ZIP › supplementary/Figure s4.tif]

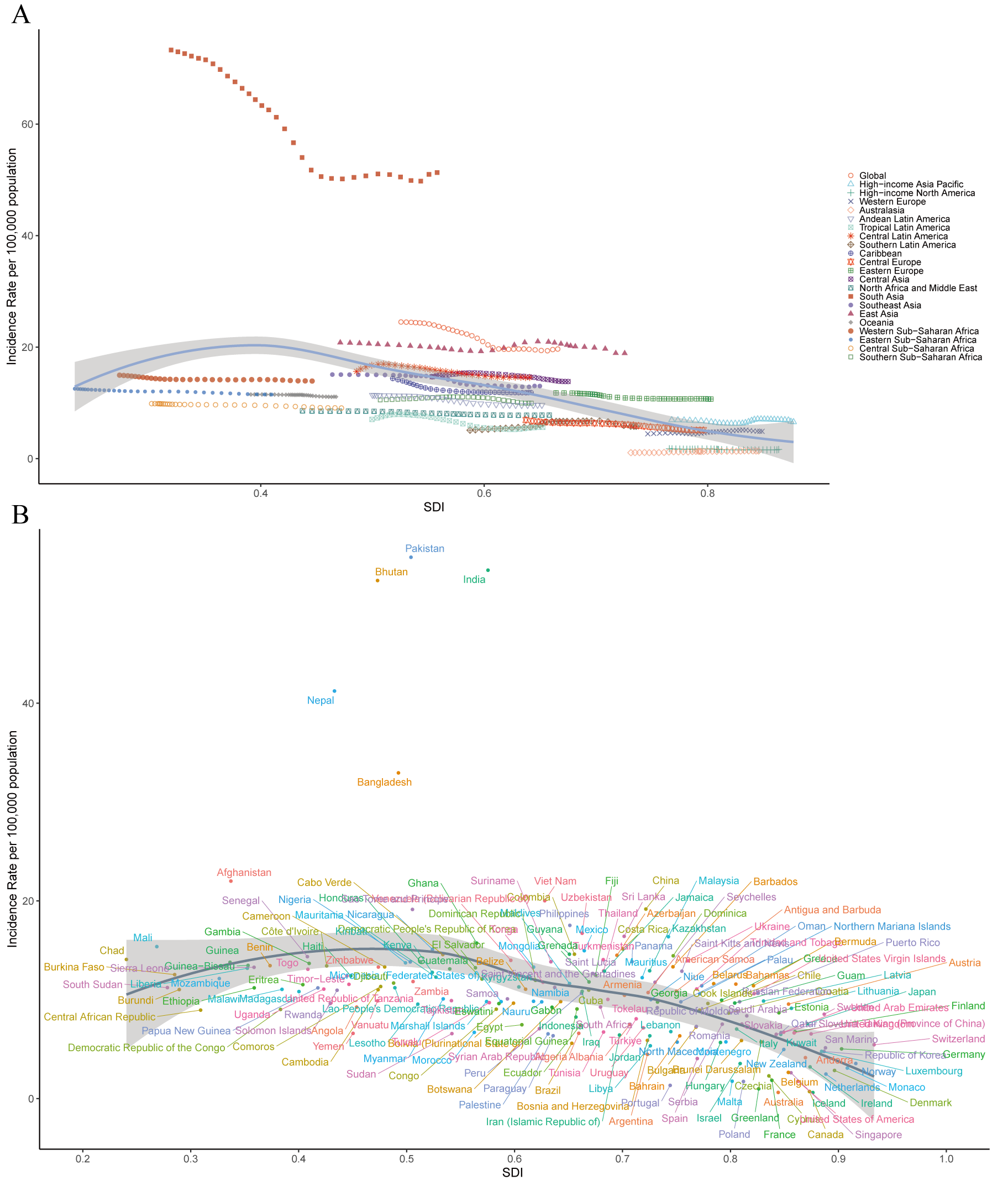

Supplement: SUPPLEMENTARY FIGURE S6 — (A) Age-standardized mortality rates of encephalitis for 21 regions by SDI from 1990–2021. The expected values based on the SDI and disease rates at all of the locations are shown as black lines. (B) Age-standardized mortality rates for encephalitis in 204 countries and territories by SDI in 2021. Expected values based on the sociodemographic index and disease rate at all of the locations are shown as black lines. SDI, sociodemographic index. [file Data_Sheet_6.ZIP › supplementary/Figure S5.tif]

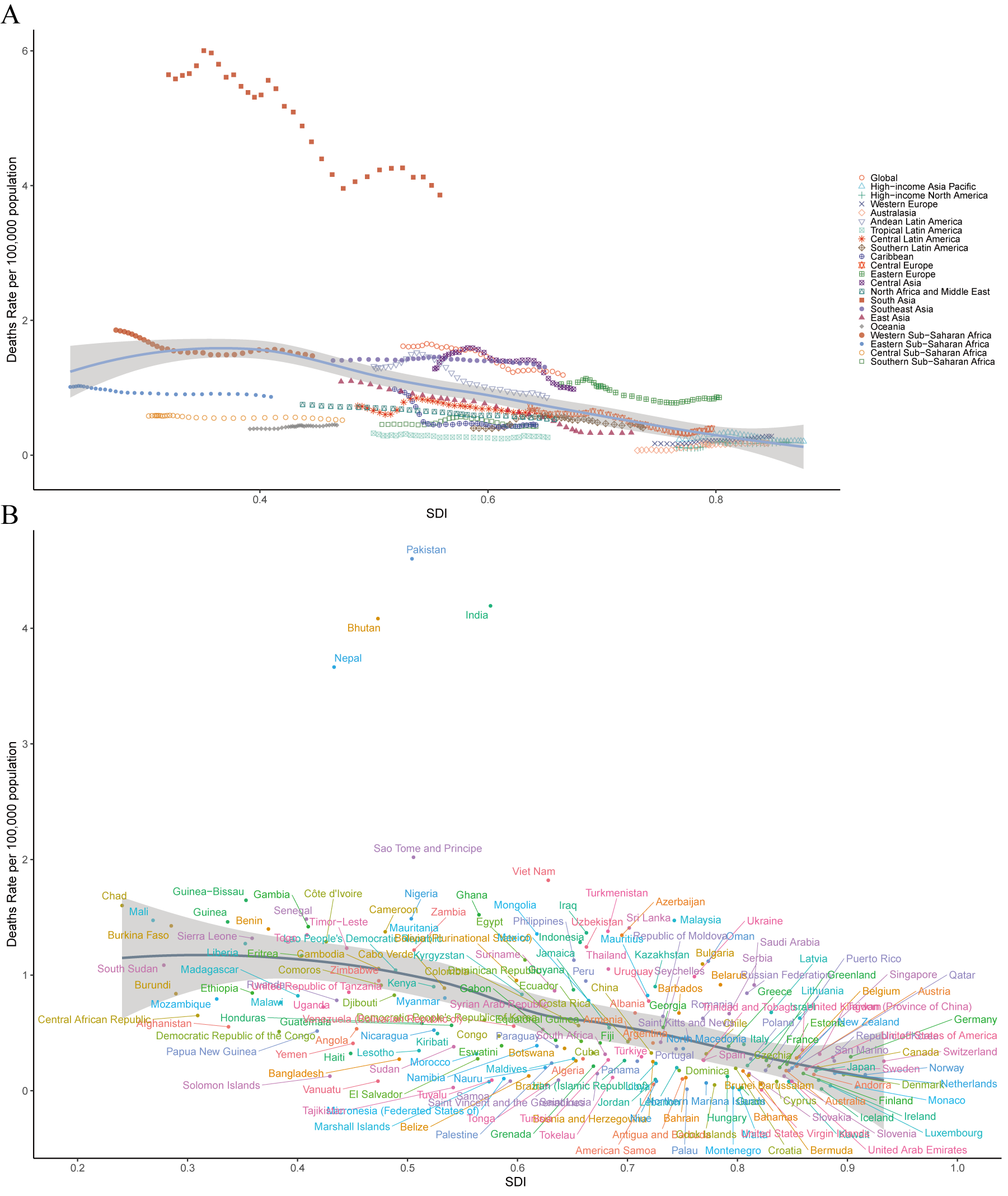

Supplement: SUPPLEMENTARY FIGURE S6 — (A) Age-standardized mortality rates of encephalitis for 21 regions by SDI from 1990–2021. The expected values based on the SDI and disease rates at all of the locations are shown as black lines. (B) Age-standardized mortality rates for encephalitis in 204 countries and territories by SDI in 2021. Expected values based on the sociodemographic index and disease rate at all of the locations are shown as black lines. SDI, sociodemographic index. [file Data_Sheet_6.ZIP › supplementary/Figure S6.tif]

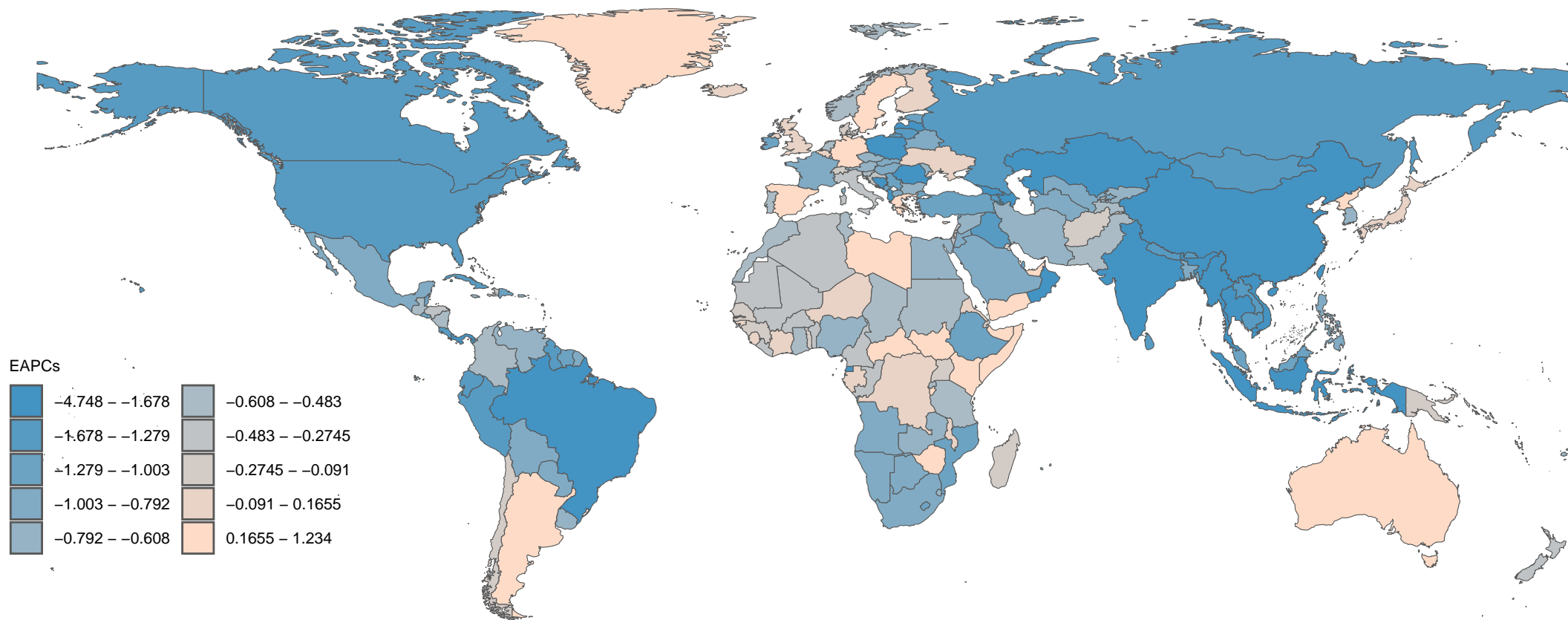

Supplement: SUPPLEMENTARY FIGURE S6 — (A) Age-standardized mortality rates of encephalitis for 21 regions by SDI from 1990–2021. The expected values based on the SDI and disease rates at all of the locations are shown as black lines. (B) Age-standardized mortality rates for encephalitis in 204 countries and territories by SDI in 2021. Expected values based on the sociodemographic index and disease rate at all of the locations are shown as black lines. SDI, sociodemographic index. [file Data_Sheet_6.ZIP › supplementary/Figure S7.pdf]

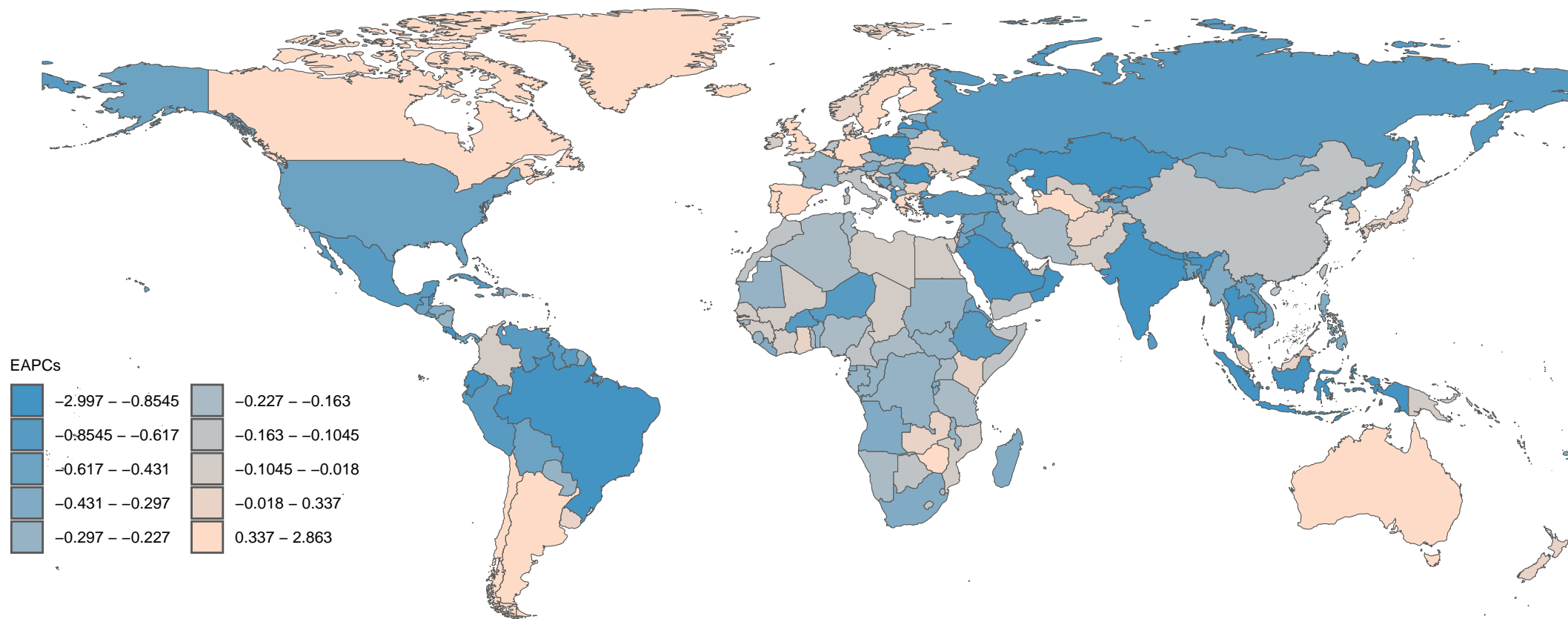

Supplement: SUPPLEMENTARY FIGURE S6 — (A) Age-standardized mortality rates of encephalitis for 21 regions by SDI from 1990–2021. The expected values based on the SDI and disease rates at all of the locations are shown as black lines. (B) Age-standardized mortality rates for encephalitis in 204 countries and territories by SDI in 2021. Expected values based on the sociodemographic index and disease rate at all of the locations are shown as black lines. SDI, sociodemographic index. [file Data_Sheet_6.ZIP › supplementary/Figure S8.pdf]

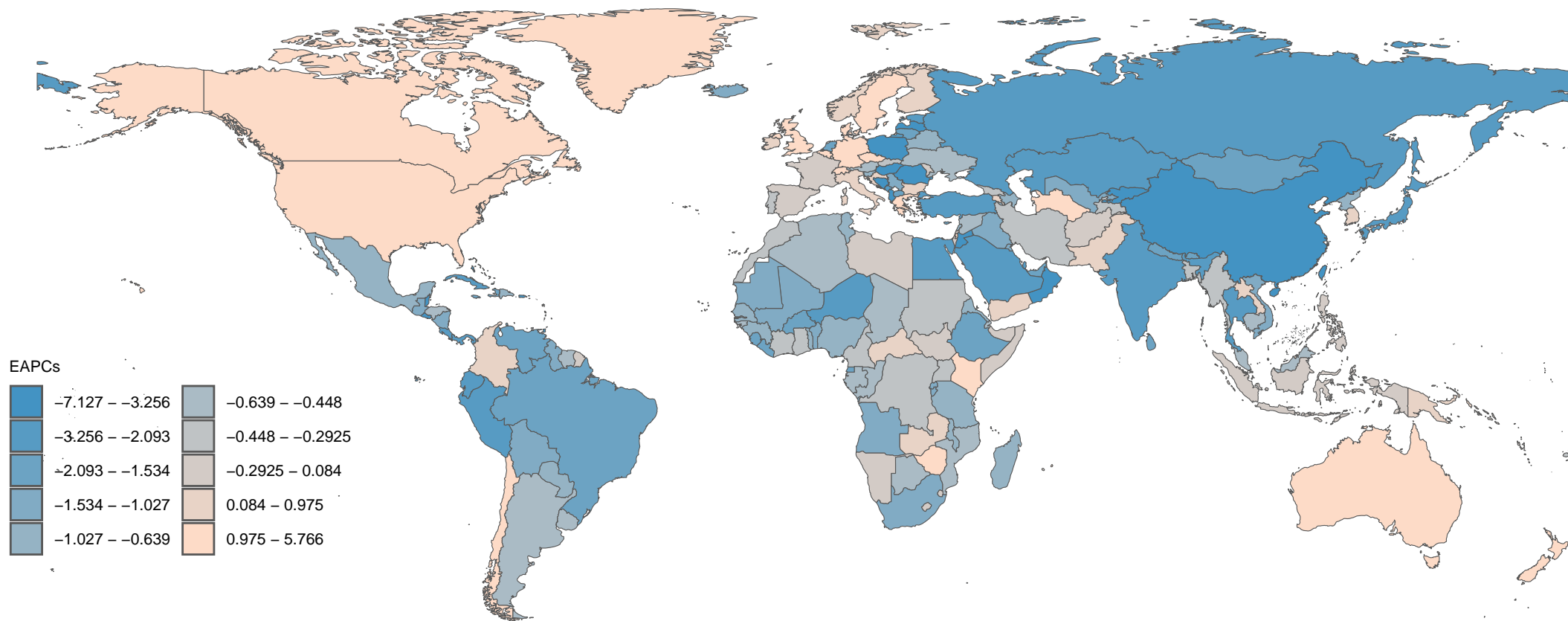

Supplement: SUPPLEMENTARY FIGURE S6 — (A) Age-standardized mortality rates of encephalitis for 21 regions by SDI from 1990–2021. The expected values based on the SDI and disease rates at all of the locations are shown as black lines. (B) Age-standardized mortality rates for encephalitis in 204 countries and territories by SDI in 2021. Expected values based on the sociodemographic index and disease rate at all of the locations are shown as black lines. SDI, sociodemographic index. [file Data_Sheet_6.ZIP › supplementary/Figure S9.pdf]
